# The health benefits of the great outdoors: A systematic review and meta-analysis of greenspace exposure and health outcomes

**DOI:** 10.1016/j.envres.2018.06.030

**Published:** 2018-10

**Authors:** Caoimhe Twohig-Bennett, Andy Jones

**Affiliations:** Norwich Medical School, University of East Anglia, Room 1.23 Queen’s Building, Norwich Research Park, Norwich NR4 7TJ, United Kingdom

**Keywords:** Greenspace, Greenness, Built environment, Natural capital, Health, Non-communicable disease

## Abstract

**Background:**

The health benefits of greenspaces have demanded the attention of policymakers since the 1800s. Although much evidence suggests greenspace exposure is beneficial for health, there exists no systematic review and meta-analysis to synthesise and quantify the impact of greenspace on a wide range of health outcomes.

**Objective:**

To quantify evidence of the impact of greenspace on a wide range of health outcomes.

**Methods:**

We searched five online databases and reference lists up to January 2017. Studies satisfying *a priori* eligibility criteria were evaluated independently by two authors.

**Results:**

We included 103 observational and 40 interventional studies investigating ~100 health outcomes. Meta-analysis results showed increased greenspace exposure was associated with decreased salivary cortisol −0.05 (95% CI −0.07, −0.04), heart rate −2.57 (95% CI −4.30, −0.83), diastolic blood pressure −1.97 (95% CI −3.45, −0.19), HDL cholesterol −0.03 (95% CI −0.05, <-0.01), low frequency heart rate variability (HRV) −0.06 (95% CI −0.08, −0.03) and increased high frequency HRV 91.87 (95% CI 50.92, 132.82), as well as decreased risk of preterm birth 0.87 (95% CI 0.80, 0.94), type II diabetes 0.72 (95% CI 0.61, 0.85), all-cause mortality 0.69 (95% CI 0.55, 0.87), small size for gestational age 0.81 (95% CI 0.76, 0.86), cardiovascular mortality 0.84 (95% CI 0.76, 0.93), and an increased incidence of good self-reported health 1.12 (95% CI 1.05, 1.19). Incidence of stroke, hypertension, dyslipidaemia, asthma, and coronary heart disease were reduced. For several non-pooled health outcomes, between 66.7% and 100% of studies showed health-denoting associations with increased greenspace exposure including neurological and cancer-related outcomes, and respiratory mortality.

**Conclusions:**

Greenspace exposure is associated with numerous health benefits in intervention and observational studies. These results are indicative of a beneficial influence of greenspace on a wide range of health outcomes. However several meta-analyses results are limited by poor study quality and high levels of heterogeneity. Green prescriptions involving greenspace use may have substantial benefits. Our findings should encourage practitioners and policymakers to give due regard to how they can create, maintain, and improve existing accessible greenspaces in deprived areas. Furthermore the development of strategies and interventions for the utilisation of such greenspaces by those who stand to benefit the most.

## Highlights

• Greenspace exposure is associated with wide ranging health benefits across 143 included studies.
• Meta-analysis showed statistically significant reductions in diastolic blood pressure, salivary cortisol and heart rate.
• Meta-analysis found statistically significant decreases in incidence of diabetes, all-cause and cardiovascular mortality.
• A number of meta-analyses were limited by poor study quality and high levels of heterogeneity.
• Greenspace and street greenery may form part of a multi-faceted approach to improve a wide range of health outcomes.

## 1 Introduction

The idea that greenspaces are beneficial for the health of the population became a generally accepted principle as early as the 1800s, when various London-based organisations including the Commons Preservation Society and the National Health Society called for the preservation, creation, and accessibility of open spaces and parks within crowded residential areas, referring to them as the “lungs” of the town or city (Hickman, 2013). More recent Healthy City guidelines from the WHO support this view, defining a healthy city as “one that continually creates and improves its physical and social environments and expands the community resources that enable people to mutually support each other in performing all the functions of life and developing to their maximum potential” (World Health Organisation, 2016a). However, increasing urbanicity and modern lifestyles can mean that opportunities for human contact with nature become less frequent.

The term greenspace is typically defined as open, undeveloped land with natural vegetation (Centres for Disease Control, 2013), although it also exists in many other forms such as urban parks and public open spaces as well as street trees and greenery. Recognition of the health benefits of greenspace exposure was one of the motivations of Oxford General Practitioner William Bird MBE in establishing the UK’s first health walk scheme at his practice in 1995, leading to the foundation of the English Walking for Health programme (WfH) (Walking for Health, 2016). Collaborations between health care providers and local nature partnerships are becoming increasingly common across the UK (Bloomfield, 2014, Kent Nature Partnership, 2014, Naturally Healthy Cambridgeshire, 2016, West of England Nature Partnership, 2016) and further afield (New Zealand Ministry of Health, 2016), and aim to better capitalise on ways the health of the natural environment is intrinsically linked to human health, striving for “healthy communities in healthy environments” (Naturally Healthy Cambridgeshire, 2016). Yet a challenge is to ensure those who might benefit the most have sufficient opportunities for exposure to greenspace.

Socioeconomic health inequalities have consistently commanded the attention of researchers and policymakers, with evidence that inequalities are currently increasing (Townsend et al., 1982). Environmental factors form one of the many potential explanations as to their cause (World Health Organisation, 2016b). Research has shown that low income neighbourhoods have reduced greenspace availability (Thomas Astell-Burt et al., 2014a, Astell-Burt et al., 2014b), and residents of more deprived neighbourhoods are less likely to use those greenspaces that exist (Jones et al., 2009). Park quality and frequency of park use have both been found to be higher amongst high-socioeconomic status (SES) residents (Leslie et al., 2010). It should also be noted that living in a greener neighbourhood has been linked with stronger greenspace-health associations (Fuertes et al., 2014, McEachan et al., 2015, Mitchell and Popham, 2007) and that income-related health inequalities have been shown to be lower in greener neighbourhoods (Mitchell and Popham, 2008). Greenspace may currently be overlooked as a resource for health and as part of a multi-component approach to decrease health inequalities.

Several hypotheses have been suggested to explain the relationship between nature and health and well-being. The first, is that natural and green areas promote health due to the opportunities for physical activity that they present. The health benefits of physical activity are well understood, with literature suggesting that exercising in a green environment may be more salutogenic than exercising in an indoor gym environment (Thompson Coon JB et al., 2011). Secondly, public greenspaces have been associated with social interaction, which can contribute towards improved well-being (Maas et al., 2009). Thirdly, exposure to sunlight, which is thought to counteract seasonal affective disorder (Rosenthal et al., 1984) and a source of vitamin D (van der Wielen RdG et al., 1995) has been suggested as a causative pathway for this relationship. A fourth is the “Old friends” hypothesis, which proposes that use of greenspace increases exposure to a range of micro-organisms, including bacteria, protozoa and helminths, which are abundant in nature and may be important for the development of the immune system and for regulation of inflammatory responses (Rook, 2013). Further potential mechanisms include the cooling influence of bodies of greenspace on surface radiating temperature (SRT), which has been documented as beneficial for health (Shin and Lee, 2005), as well as the mitigation of greenspace against environmental hazards such as air (Dadvand et al., 2012a, Yang et al., 2005) and noise pollution (De Ridder et al., 2004, Wolch et al., 2014).

Whilst there is a growing body of literature attempting to quantify the links between nature and improved health and well-being, systematic reviews in this area have largely focused on the association between greenspace and a specific health outcome or behaviour such as mortality (Gascon et al., 2016, van den Berg et al., 2015), obesity (Lachowycz and Jones, 2011), birth weight (Dzhambov et al., 2014), physical wellbeing (Thompson Coon JB et al., 2011) as well as the acute health benefits of short term exposure to greenspace (Bowler et al., 2010). Associations have been reported with improved perceived general health, perceived mental health, as well as linking quality of neighbourhood greenness with improved general health (van den Berg et al., 2015). Physical activity in a natural outdoor environment has been associated with reduced negative emotions and fatigue, increased energy (Bowler et al., 2010, Thompson Coon JB et al., 2011), improved attention, as well as greater satisfaction, enjoyment and a greater intent to repeat the activity (Bowler et al., 2010). Additionally, meta-analyses have shown increased residential greenspace to be significantly associated with reduced cardiovascular and all-cause mortality (Gascon et al., 2016), and increased birth weight (Dzhambov et al., 2014). Yet no systematic review has attempted to determine the impact of greenspace on a wide range of health outcomes.

With this systematic review, we aim to address a major gap in the evidence by identifying a set of health outcomes that have been investigated as being potentially associated with exposure to greenspace. Health outcome terms were taken from the 10th revision of the International Statistical Classification of Diseases and Related Health Problems (ICD-10), a medical classification list produced by the World Health Organisation (World Health Organisation, 2015), with greenspace terms taken from a previous systematic review (Lachowycz and Jones, 2011). The clarification of the magnitude of associations facilitates the investigation of potential underlying mechanisms in the relationship between nature and health. Furthermore, clinicians may use these findings to make recommendations to patients, which may convey health benefits or assist in tackling socio-economic health inequalities.

## 2 Methods

This systematic review followed Cochrane systematic review guidelines (Deeks et al., 2011), requirements of the NHS National Institute of Health Research Centre for Reviews and Dissemination (PROSPERO, 2015) and the PRISMA statement for reporting studies that evaluate healthcare interventions (Liberati et al., 2009, Moher et al., 2009). Methods of the analysis and inclusion criteria were specified in advance and documented in a protocol registered as CRD42015025193 (PROSPERO, 2015) available on the PROSPERO database http://www.crd.york.ac.uk/prospero/.

### 2.1 Data sources

We searched electronic databases including MEDLINE (US National Library of Medicine, Bethesda, Maryland, U.S.), EMBASE (Reed Elsevier PLC, Amsterdam, Netherlands), AMED (Wolters Kluwer, Leicestershire, UK), CINAHL (EBSCO Publishing, Massachusetts, U.S.) and PsycINFO (American Psychological Association, Washington D.C., U.S.) from inception to the end of September 2015, using specific search terms. The search was then updated to include studies published until mid-January 2017. Databases were selected to best represent source material in health, allied health and human science. Additionally, reference lists from included studies and previous systematic reviews on greenspace and health were hand searched.

### 2.2 Search strategy

Search terms associated with greenspace were developed with reference to a previous systematic review on greenspace and obesity (Lachowycz and Jones, 2011). For this review, we defined ‘greenspace’ as open, undeveloped land with natural vegetation as well as urban greenspaces, which included urban parks and street greenery. Health outcomes were taken from ICD-10 and then expanded to include the relevant metrics, for example “diabetes” was expanded to include “blood glucose” and glycated haemoglobin, commonly referred to as “HbA1c.” To limit the scope of work, mental health and communicable diseases were excluded from this review due to the volume of literature after including them in initial scoping searches. Outcomes associated with weight status and birth weight were also excluded, as systematic reviews investigating them have recently been published (Dzhambov et al., 2014, Lachowycz and Jones, 2011, Thompson Coon JB et al., 2011).

The search strategy identified studies that contained at least one keyword or Medical Subject Heading (MeSH) from each list of search terms. The search was piloted to ensure known studies were identified and search syntax terms were adapted to suit each database. The electronic database search terms are detailed in the online supplementary table S2 (Appendix A). The search strategy also incorporated limits to studies conducted on humans and studies written in English.

### 2.3 Study selection

All empirical studies where the outcome could be directly attributable to greenspace were included, including both intervention and observational studies. Titles and abstracts were examined by the primary reviewer (CB) to assess eligibility for the review using PICO criteria:

• **Participants:** Male and female, no age restrictions
• **Intervention:** Exposure to greenspace
• **Comparators:**There is no comparator restriction
• **Outcomes:** Any health outcome

Further details of the inclusion and exclusion criteria can be found in Table 1, below.

**Table 1:** Inclusion and exclusion criteria.

*Inclusion criteria for this review are:*	*Exclusion criteria*
Empirical studies testing the relationships between greenspace and physical health outcomes	Studies that do not look at empirical evidence.
Studies that use human participants.	Studies that do not use human participants.
The study reports a physical health outcome other than BMI/physical activity/mental health/communicable disease/birth weight.	Studies where BMI/mental health/communicable disease/birth weight are the only outcome(s) or the study does not report a health outcome.
Papers and documents written in English.	Papers and documents not written in English.

Reviewer (CB) initially screened titles and abstracts to remove obviously irrelevant articles, and then two reviewers screened all full text articles independently (CB & AJ) to identify studies for inclusion in the systematic review. Discrepancies were resolved by discussion. Frequently abstracts used terms such as “neighbourhood environment”, “built environment” or “neighbourhood facilities” and did not specify the definition of these terms or if greenspace was investigated. These studies were retrieved as full texts and screened for greenspace as an outcome to ensure that none were excluded erroneously.

### 2.4 Data extraction

A data extraction sheet was developed by both authors to record the study type, population, type of greenspace under investigation, greenspace measurement tool used, health outcome under investigation and the outcomes. This was piloted on four manuscripts and refined accordingly. Data was extracted into a coding frame using Microsoft Excel, synthesised and tabulated. All studies underwent methodological critical appraisal using one of two checklists. For intervention studies, we used a risk of bias tool employed by Hanson and Jones (Hanson and Jones, 2015) and Ogilvie et al. (Ogilvie et al., 2007), (Table 3) which was adapted for purpose. For observational studies the Lachowycz and Jones (Lachowycz and Jones, 2011) quality checklist (Table 2) was adapted and used. Publication bias across studies within the meta-analysis was tested with funnel plots using SE as the measure of study size on the vertical axis and mean difference on the horizontal.

**Table 2:** Adapted Lachowycz and Jones quality appraisal checklist for observational studies.

**Item**	**Description**	**Scale**
**Methodological quality**		
1. Population - Selection bias	Are the individuals selected to participate in the study likely to be representative of the target population?	1: Likely to be representative
		0: Unlikely to be representative
		N: Insufficiently described
2. Population –Inclusion bias	Is there evidence of bias in the percentage of selected individuals who provided data for inclusion in the analysis?	1: No evidence of bias
		0: Evidence of bias
		N: Insufficiently described
3. Outcome measure	Was the outcome objectively measured or self- reported?	1: Objectively measured outcome
		0: Self reported
		N: Insufficiently described
4. Green space measure - derivation	Was derivation of the green space variable well described?	1: Derivation of green space measure well described
		0: Derivation of green space measure not well described
5. Green space measure - type	Did the green space measure include information on type of green space?	1: Green space measure included information on type of green space
		0: Green space measure did not include information on type of green space
		N: Insufficiently described
6. Use of green space	Use of green space was measured and included in analysis	1: Measured use of green space
		0: Did not measure use of green space
		N: Insufficiently described
7. Statistical methodology	Was an appropriate statistical methodology used?	1: Evidence of appropriate methodology
		0: No evidence of appropriate methodology
		N: Insufficiently described
8. Effect size	Was an effect size reported for green space variable?	1: Effect size reported for green space
		0: Effect size not reported for green space
		N: Insufficiently described
9. Multiplicity	Was green space the main exposure being measured or one of many variables being tested?	1: Green space variable main exposure
		0: Green space variable one of many variables being tested
		N: Insufficiently described
10. Level of analysis	Was analysis of green space in relation to outcome carried out at individual level or at ecological (area) level	1: Individual level
		0: Ecological level
		N: Insufficiently described
11. Green space measure	Was greenspace exposure objectively measured or self-reported?	1: Objectively measured
		0: Self-reported
		N: Insufficiently described

### 2.5 Narrative synthesis and meta-synthesis

Following critical review of each study, a narrative synthesis was compiled. In order to be considered for meta-analysis, authors needed to present either 1) mean difference, standard deviation (SD) and sample size for both the highest and lowest greenspace categories, or 2) number of cases of the reported condition/disease as well as sample size for both highest and lowest greenspace categories. If the required data was not reported in the paper, authors were contacted for this information. In total, 92 authors were contacted of which 32 responded with the data required for meta-analysis. In order for a specific health outcome to be considered for meta-analysis data from a minimum of two studies was required. Where data was given for different subgroups, each was input separately and combined in meta-analyses using the RevMan software package. All results are presented as forest plots with 95% confidence intervals. The I^2^ statistic was calculated to quantify the degree of heterogeneity between studies (Higgins et al., 2003). A rough guide to interpreting heterogeneity is provided in the Cochrane handbook and gives I^2^ values of 30–60% to represent moderate heterogeneity and values of 50–90% to represent substantial heterogeneity (Deeks et al., 2011). In cases of high heterogeneity, the known heterogeneity was assessed (i.e. populations, study design, exposure etc) to ensure that a meta-analysis was appropriate. A random effects model was employed for all meta-analyses as it is considered to represent a more conservative approach, suitable for cases of high heterogeneity (Higgins and Green, 2011).

Sensitivity analysis was then undertaken, which included studies which only scored 9 or above (out of a total of 11) in either the risk of bias tool or quality appraisal checklist, meaning that all but 2 risk of bias/quality checklist criteria had been met.

## 3 Results

**Fig. 1:**
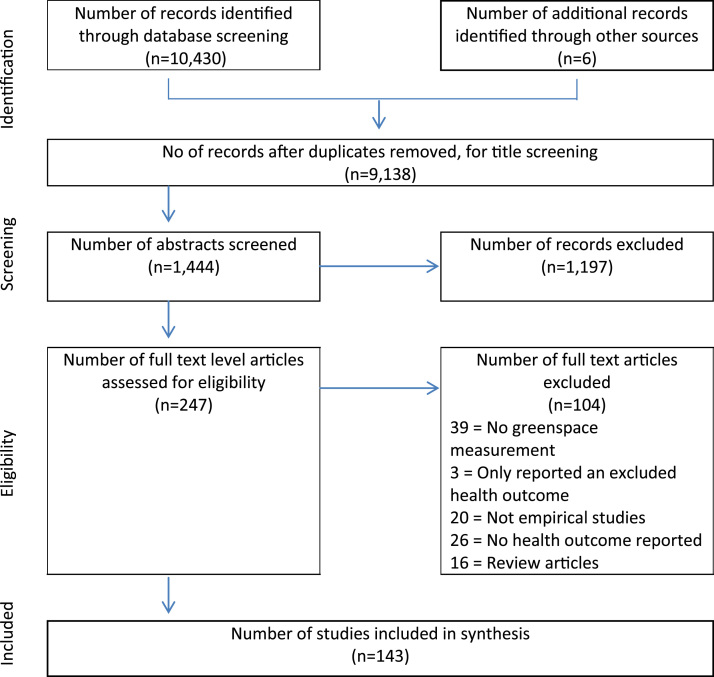
Flow chart of studies.

The initial database search yielded 10,430 studies, of which 8986 were removed as duplicates or as clearly irrelevant after reviewing titles. A further 6 studies were retrieved from reference lists of review articles. The abstracts of 1444 studies were screened and any that did not provide enough information were retrieved for full text examination. A total of 247 papers were read as full texts to be assessed for eligibility. After independent assessment by the second reviewer (AJ), 143 studies met the inclusion criteria and were eligible to be included in the synthesis. The review flow chart is detailed in Fig. 1. The characteristics and synthesised results for all 143 papers are detailed in supplementary table S1 (Appendix A).

### 3.1 Study characteristics

Although there was no date restriction on the search, 96% of the articles were studies from the past 10 years, illustrating recent growth in interest in greenspace and health, with no papers prior to 1984 meeting the inclusion criteria. Studies were in 20 different countries. Although 50% of studies were in Europe, the country with the highest frequency of included studies was Japan with 24. The populations under investigation varied greatly in size, with the smallest an intervention study of 9 participants (Ochiai et al., 2015), the largest study using primary data collection presented results for 2593 primary schoolchildren (Dadvand et al., 2015), and the largest study using routinely collected data used 2011 UK census data with a population of >63 million (Wheeler et al., 2015). In some papers, the number of participants was not reported.

Eleven different types of greenspace exposure were measured, the most common of which was neighbourhood greenspace (including residential greenspace, street greenery and tree canopy) measured by 56 studies, followed by greenspace-based interventions and proximity to a large greenspace. Several randomised studies compared a known green environment (i.e. a park or forest) with an urban or indoor environment. One study examined whether viewing trees through a hospital window had any association with post-operative recovery time when compared with a window view of a wall with no trees (Ulrich, 1984). One included study investigated both green and blue (water) space (Burkart et al., 2016). Studies investigating blue space alone with no investigation of greenspace exposure were excluded at the full text screening stage. A variety of greenspace measurement tools were used, including Normalised Difference Vegetation Index (NDVI), the Centre for Ecology and Hydrology (CeH) land cover map, and tree canopy and street tree data, as well as subjective measures of greenness such as self-reported quality of neighbourhood greenspace and self-reported frequency of walking in a green area.

Within the 143 studies, 40 were interventional and the remainder observational. Out of the 40 interventional studies, 27 were investigating the association between shinrin-yoku and various health outcomes. Shinrin yoku, or “forest bathing” is a popular practice in Japan and neighbouring countries, and is defined as “taking in the atmosphere of the forest” (Park et al., 2010). It is said to have health-promoting properties and to reduce stress (Park et al., 2010). Participants of shinrin-yoku spend time in the forest either sitting or lying down, or walking through the forest. In studies investigating forest bathing, a control group carried out the same activity in an urban environment. These studies typically had small numbers of participants (between 9 and 280 participants).

Of the 103 observational studies, 35 were cohort studies and 69 cross-sectional, including 18 large scale ecological studies investigating environmental influences on health amongst the population using census data. Almost 100 health outcomes were investigated, with most manuscripts investigating more than one outcome. The most frequently investigated health outcomes were cardiovascular, including cardiovascular mortality, blood pressure, heart rate and incidence of angina and myocardial infarction. Other commonly reported health outcomes included pregnancy outcomes, self-reported health, mortality (all-cause, respiratory and intentional self-harm), and diabetes, as well as various blood biomarkers. The individual health outcomes investigated by each study are detailed in the table of study characteristics, supplementary table S1 (Appendix A).

### 3.2 Study quality

All 143 articles were assessed for quality using adapted versions of the Lachowycz and Jones checklist (Lachowycz and Jones, 2011) for observational studies (Table 2) and the Hanson and Jones and Ogilvie et al. risk of bias tool (Hanson and Jones, 2015, Ogilvie et al., 2007) for interventional studies (Table 3). No study was excluded due to a low quality score. Assessments of quality were initially made by the first reviewer (CB) and then all studies were cross-checked by one other (AJ, SH or EC) for discrepancies.

**Table 3:** Adapted Hanson and Jones and Ogilvie et al. risk of bias tool for intervention studies.

**Item**	**Description**	**Scale**
**Methodological quality**		
1. Reporting: hypothesis	Is the hypothesis/aim/objective of the study clearly described?	1: Yes – clearly described
		0: No
2. Reporting: outcome(s)	Are the main outcomes to be measured clearly described in the introduction or methods section? (if the main outcomes are first mentioned in the results section, this question should be answered no)	1: Yes – clearly described in introduction/methods
		0: No – not clearly described/first mentioned in results
3. Reporting: intervention	Are the interventions of interest (greenspace and control or otherwise) clearly described?	1: Yes – clearly described
		0: No
4. Randomisation	Was there sufficient description of a randomisation process or statistical test to show that comparability between the two groups has been adjusted for (no explanation scores zero)?	1: Yes – description of a randomisation process
		0: No – no explanation
5. Exposure	Did the authors show that there was no evidence of a concurrent intervention which could have influenced the results (no explanation scores zero)?	1: Yes
		0: No – no explanation
		N: Insufficiently described
6. Representativeness	Were the study samples shown to be representative of the study population?	1: Yes – shown to be representative
		0: No – shown not to be representative
		N: Insufficiently described
7. Comparability	Were baseline characteristics of the intervention comparable with the control or were potential confounders at baseline approximately adjusted for in analysis?	1: Yes
		0: No
		N: Insufficiently described
8. Attrition	Were numbers of participants at follow-up identifiable as at least 80% of the baseline?	1: Yes
		0: No
		N: Insufficiently described
9. Outcome assessment: tools	Were valid and reliable tools used to assess participant outcomes?	1: Yes
		0: No
		N: Insufficiently described
10. Follow-up time scale	Was the length of time to follow up assessment appropriate for the intervention?	1: Yes
		0: No
11. Precision of the results	Were confidence intervals or p-values given?	1: Yes
		0: No

An inter-rater reliability analysis using the κ statistic was performed and found κ 0.937, p < 0.001 representing substantial agreement. Full consensus was reached after discussion. In the case that a checklist item consistently brought up discrepancies, clarification of the definition of the item was discussed. Individual quality analysis scores can be found in the supplementary tables S5 (observational studies) and S6 (intervention studies) (Appendix B).

For the 103 observational studies assessed using the Lachowycz and Jones checklist (Lachowycz and Jones, 2011) detailed in Table 2, scores ranged from 4 (one study) to 11 (one study), out of a total of 11 criteria. Only 12.6% of studies scored ≤ 7, with 39.8% of studies scoring 9 out of 11. The two checklist criteria which were the most recurrently missing from were “*5. Did the green space measure include information on type of greenspace?”* and “*6. Use of greenspace was measured and included in the analysis”.*

For the 40 interventional studies assessed using the Hanson and Jones and Ogilvie et al. risk of bias tool (Hanson and Jones, 2015, Ogilvie et al., 2007) detailed in Table 3, scores ranged from 5 (one study) to 11 (one study) out of a total of 11 criteria. Only 7.7% of studies scored ≤ 7, with 66.7% of studies scoring 9 out of 11. The two checklist criteria which were the most recurrently missing from studies were “*5. Did the authors show that there was no evidence of a concurrent intervention which could have influenced the results?”* and “*6. Were the study samples shown to be representative of the study population?”*

### 3.3 Meta-analysis

**Fig. 2:**
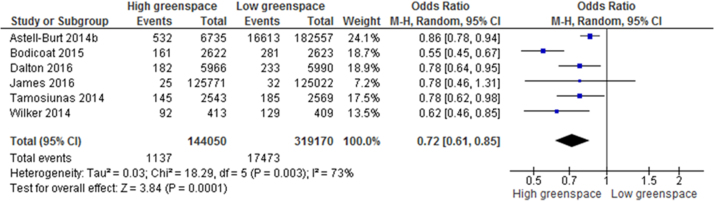
Meta-analysis of the effects of greenspace exposure on incidence of type II diabetes.

**Fig. 3:**
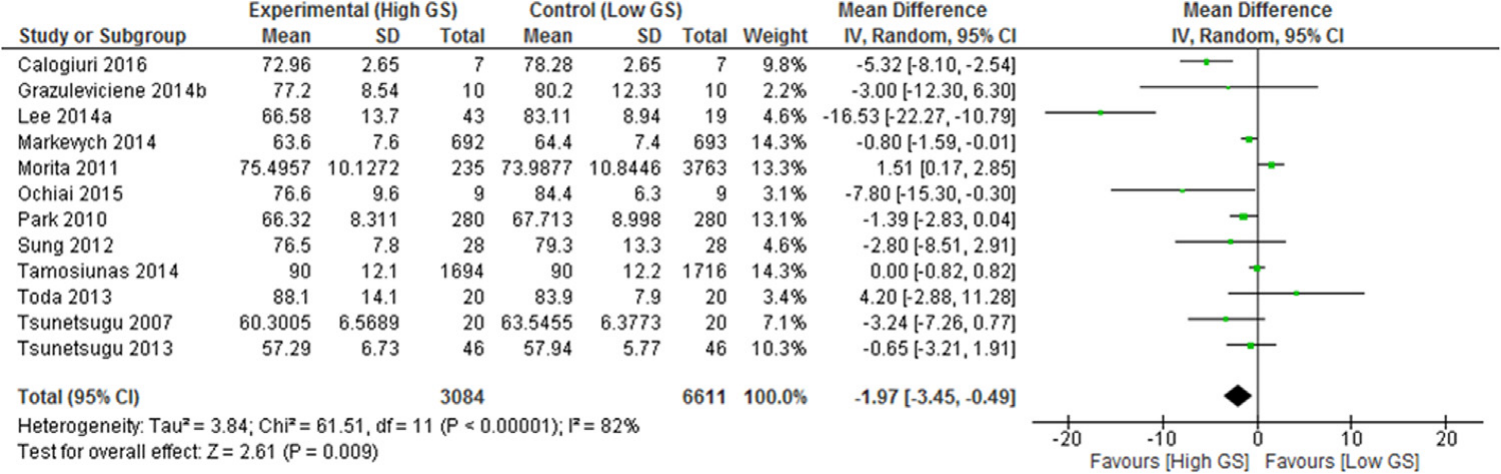
Meta-analysis of the effects of greenspace exposure on diastolic blood pressure.

When extracting information from papers for meta-analysis, ‘high’ and ‘low’ greenspace exposure was defined based on the highest and lowest exposure categories provided in each paper. These were typically the highest or lowest quartile or quintile of exposure.” Commonly reported outcome measures enabled meta-analysis of 24 health outcomes, summarised in Table 4 and presented in full in supplementary Figs. S2-S25 (Appendix B). Statistically significant health denoting associations between high versus low greenspace exposure groups were identified for self-reported health, type II diabetes (Fig. 2), all-cause and cardiovascular mortality, diastolic blood pressure (Fig. 3), salivary cortisol, heart rate, heart rate variability (HRV), and HDL cholesterol as well as preterm birth and small size for gestational age births. Reductions were also found for incidence of stroke, hypertension, dyslipidaemia, asthma, and coronary heart disease, as well as improvements in systolic blood pressure, fasting blood glucose, and gestational age. However these results were not statistically significant.

**Table 4:** Summary meta-analysis results table: mean difference (MD) between highest and lowest greenspace exposure groups.

**Outcome**	**N (participants)**	**Effect MD (95% CI)**	**Heterogeneity I**^**2**^	**P-value**
*Salivary cortisol*	*7 (954)*	*− 0.05 (−0.07, −0.04)*	*0%*	*P < 0.001*
*Heart rate*	*10 (1058)*	*− 2.57 (−4.30, −0.83)*	*78%*	*P0.004*
*HDL cholesterol*	*2 (3474)*	*− 0.03 (−0.05,* <−0.01*)*	*0%*	*p = 0.02*
*Diastolic blood pressure*	*12 (9695)*	*− 1.97 (−3.45, −0.49)*	*82%*	*p = 0.009*
Systolic blood pressure	13 (9791)	− 1.50 (−3.43, 0.44)	78%	p = 0.13
*Change in HF power of HRV*	*7 (826)*	*91.87 (50.92, 132.82))*	*49%*	*p < 0.001*
*LF/(LF+HF)*	*6 (266)*	*− 0.06 (−0.08, −0.03)*	*0%*	*p < 0.001*
HbA1c	2 (174)	− 0.77 (−1.86, 0.32)	54%	P = 0.16
Fasting blood glucose	2 (3474)	− 0.01 (−0.08, 0.07)	0%	p = 0.84
Total cholesterol	2 (3474)	0.03 (−0,05, 0.10)	0%	p = 0.48
LDL cholesterol	2 (3474)	0.04 (−0.03, 0.11)	0%	p = 0.23
Triglycerides	2 (3474)	0.06 (−0.01, 0.12)	0%	p = 0.07
Gestational age	3 (22911)	< −0.01 (−0.05, 0.05)	0%	P = 0.94

**Table 5:** Summary meta-analysis results table: odds ratios of disease incidence difference between high and low greenspace areas.

**Outcome**	**N (participants)**	**Odds ratio (95% CI)**	**Heterogeneity I**^**2**^	**P-value**
*Good self-reported health*	*10 (41873103)*	*1.12 (1.05, 1.19)*	*100%*	*p < 0.001*
*Preterm birth*	*6 (1593471)*	*0.87 (0.80, 0.94)*	*68%*	*p < 0.001*
*Type II diabetes*	*6 (463220)*	*0.72 (0.61, 0.85)*	*73%*	*p < 0.001*
*All-cause mortality*	*4 (4001035)*	*0.69 (0.55, 0.87)*	*96%*	*P = 0.002*
Hypertension	4 (11228)	0.99 (0.81, 1.20)	62%	P = 0.91
*Small for gestational age*	*4 (1576253)*	*0.81 (0.76, 0.86)*	*65%*	*p < 0.001*
*Cardiovascular mortality*	*2 (3999943)*	*0.84 (0.76, 0.93)*	*54%*	*p < 0.001*
Stroke	3 (256727)	0.82 (0.61, 1.11)	59%	P = 0.20
Dyslipidaemia	2 (5934)	0.94 (0.75, 1.17)	57%	P = 0.56
Asthma	2 (2878)	0.93 (0.57, 1.52)	68%	P = 0.78
Coronary heart disease	2 (255905)	0.92 (0.78, 1.07)	48%	P = 0.26

Zero heterogeneity was reported for 8 of the analyses, 6 reported moderate heterogeneity (30–60%) with 9 having substantial heterogeneity (>60%). This suggests substantial heterogeneity between studies for heart rate, diastolic and systolic blood pressure, self-reported health, preterm birth, diabetes, all-cause mortality, small size for gestational age, hypertension and asthma. The I^2^ score for the good self-reported health meta-analysis was 100%, indicating very high levels of inconsistency between studies. Using funnel plots, all studies were identified as visually symmetrical with a narrow spread at the top of the funnel indicating precision with results close to the pooled estimate and without bias towards smaller studies. Supplementary Fig. S1 (Appendix B) shows an example funnel plot.

To test whether significant meta-analysis results were due to inclusion of poor quality studies, sensitivity analysis was conducted where possible. Meta-analysis was repeated with only studies that scored ≥9 in either the quality appraisal checklist or risk of bias tool. This was only possible for heart rate, which showed a stronger effect size −3.46 (95% CI −4.05, −2.88) (2 studies removed), systolic blood pressure, which decreased in effect size and remained statistically non-significant −0.49 (95% CI −1.20, 0.22) (2 studies removed), and self-reported good health, which decreased in effect size and lost significance 1.06 (95% CI 0.96, 1.18) (6 studies removed). Table 6 shows the results from this sensitivity analysis. Fasting blood glucose, cholesterol, HbA1c, asthma, and triglycerides meta-analyses were not possible to include as there was only one remaining high quality study. The remaining meta-analyses consisted only of studies scoring ≥9, and so sensitivity analysis was not possible.

**Table 6:** Summary results table of sensitivity analysis meta-analysis consisting of only studies which scored ≥9 in quality checklist or risk of bias tool.

**Outcome**	**N (participants)**	**Effect MD or odds ratio (95% CI)**	**Heterogeneity I**^**2**^	**P-value**
*Heart rate*	*8 (842)*	*− 3.46 (−4.05, −2.88)*	*83%*	*P < 0.00001*
Systolic blood pressure	11 (9681)	− 0.49 (−1.20, 0.22)	79%	p = 0.17
Good self-reported health	4 (6577)	1.06 (0.96, 1.18)	88%	P = 0.26

### 3.4 Non-pooled health outcomes

Meta-analysis was not possible for a number of health outcomes including cancer, respiratory mortality, neurological outcomes, and various biomarkers, as no two studies presented results on comparable outcomes. Three studies reported on cancer outcomes and found that living in the highest quartile of greenspace was associated with a significantly reduced risk of prostate cancer (Demoury et al., 2017), OR 0.82 (95% CI 0.72, 0.92), as well as reduced incidence of overall cancer mortality HR 0.87 (95% CI 0.78, 0.97) (James et al., 2016), whilst an Australian study found a significant increased risk of skin cancer for participants living in the highest greenspace quartile OR 1.07 (95% CI 1.01, 1.14) Astell-Burt et al., 2014a, Astell-Burt et al., 2014b). One study found living in the highest quartile of greenspace to be associated with reduced incidence of respiratory mortality (James et al., 2016) HR 0.66 (95% CI 0.52, 0.84). In terms of neurological outcomes, one study found that living in a neighbourhood with a low % of greenspace was associated with deficits in motor development in children (Kabisch et al., 2016), whilst another found no association between greenspace and cognitive development (Ward et al., 2016). A number of studies investigated a variety of biomarkers including natural killer cells (Kim et al., 2015), C-reactive protein (Mao et al., 2012b), and perforin (Jia et al., 2016). Individual study results can be found in the table of study characteristics, supplementary table S1 (Appendix A).

## 4 Discussion

This systematic review and meta-analysis of 143 studies provides evidence that exposure to greenspace is associated with wide-ranging health benefits. Meta-analyses results have shown statistically significant health-denoting associations for salivary cortisol −0.06 (95% CI −0.07, −0.04), heart rate −3.47 (95% CI −4.04, −2.90), diastolic blood pressure −1.97 (95% CI −3.45, −0.49), HDL cholesterol −0.03 (95% CI −0.05, <-0.01), and significant improvements in the HF power 91.87 (95% CI 50.92, 132.82) and LF/(LF+HF) −0.06 (95% CI −0.08, −0.03) of heart rate variability. As well as statistically significant reductions in the incidences of type II diabetes 0.72 (95% CI 0.61, 0.85), all-cause mortality 0.69 (95% CI 0.55, 0.87), cardiovascular mortality 0.84 (95% CI 0.76, 0.93), as well as pregnancy outcomes preterm birth 0.87 (95% CI 0.80, 0.94), and small size for gestational age 0.81 (95% CI 0.76, 0.86). A significant increase in incidence of reporting good health was also found 1.12 (95% CI 1.05, 1.19). Some of the meta-analyses results had high levels of heterogeneity (Table 4, Table 5), and should therefore be interpreted with caution. Included studies investigating non-pooled health outcomes also reported salutogenic associations for health outcomes such as cancer outcomes, respiratory mortality, sleep duration, various biomarkers, and neurological outcomes.

This review has comprehensively sought out empirically-reported studies investigating the association between greenspace and a wide range of health outcomes across five databases, covering a large number of relevant international journals. It has extensively analysed 143 different studies with the combined population size of > 290 million. It has also extracted information for 24 novel meta-analyses to provide evidence of health benefits. A further major strength of this review is its inclusivity; studies were not excluded based on study design or type of greenspace, and as a result a broad range of greenspace exposures and health outcomes were identified by the 143 included studies. However, the inclusivity of this study can also be viewed as a limitation due to high heterogeneity across studies, and difficulties in comparing results from small-scale intervention studies and much larger ecological cross-sectional studies or in comparing studies that used objective measurements of greenspace with those that did not.

A number of studies reported stronger associations between greenspace exposure and self-reported health, birth outcomes and morbidity for those from low socioeconomic status (SES) groups and the most deprived areas (Agay-Shay et al., 2014, Dadvand et al., 2012b, Mitchell and Popham, 2008, Roe et al., 2016). Similar stronger associations were reported for birth outcomes and self-reported health for those with <10 years in education. Increased neighbourhood greenness was also reported to decrease the effect of income deprivation on both all cause and cardiovascular mortality by one study (Mitchell and Popham, 2008). However results by SES group were only presented by a small number of studies so it was not possible to conduct a formal subgroup analysis, or to determine if this was the case for other health outcomes. Greenspaces may form part of the arsenal for combatting health inequalities, and our findings should encourage practitioners and policymakers to give due regard to how they can create, maintain and improve existing accessible greenspaces in deprived areas. Furthermore, the development of strategies and interventions for the utilisation of such greenspaces by those of low SES status who stand to benefit the most is needed.

Whilst previous systematic reviews have examined the relationship between greenspace and specific health outcomes or behaviours, this review investigated the potential impact of greenspace on a broad range of health outcomes. Our findings are consistent with previous systematic review results that suggest that greenspace is beneficial for health. Lachowycz and Jones (Lachowycz and Jones, 2011) found that 68% of papers included in their systematic review found a positive or weak association between greenspace and obesity-related health indicators, although findings were inconsistent and mixed. Thompson Coon et al. investigated the association between exercising in outdoor natural areas and health, and found physical activity in natural environments to be associated with increased energy, improved mental wellbeing and higher levels of intent in repeating the activity at a later date (Thompson Coon JB et al., 2011). However, consistent with our systematic review, poor methodological quality of the available evidence and the heterogeneity of outcome measures hamper the interpretation and extrapolation of these findings (Thompson Coon JB et al., 2011). Bowler et al. looked at studies comparing measurements of health in outdoor natural and synthetic environments such as indoor or outdoor built environments (Bowler et al., 2010). Findings suggest that a walk or run in a natural environment may convey greater health benefits than the same activity in a synthetic environment. This is consistent with the findings of Hanson and Jones, who conducted a systematic review and meta-analysis on outdoor walking groups (Hanson and Jones, 2015). Outdoor walking groups were found to significantly improve systolic and diastolic blood pressure, heart rate, body fat percentage, BMI, cholesterol, V02 max, depression and physical functioning, with no adverse side effects reported (Hanson and Jones, 2015). As with Bowler’s systematic review and our findings, the evidence suggests that walking in a greenspace or natural area may offer health benefits above walking in an urban environment or on a treadmill (Bowler et al., 2010). Putting aside the health benefits of physical activity, which have been widely documented (Bize et al., 2007, Janssen and LeBlanc, 2010, Lawlor and Hopker, 2001, Penedo and Dahn, 2005, Warburton et al., 2006), the associations between greenspace and health found in this study suggests that “green exercise” may have additional health benefits. In combination with the findings of our systematic review, it can be seen that there is a convincing body of evidence to suggest that greenspace is beneficial for health, and also that greenspace may be currently undervalued as a resource for health. Studies consistently reported that there are several substantial gaps in knowledge remaining in this field, most commonly the mechanisms underlying the relationship between greenspace and health.

A high proportion of studies included in meta-analyses investigated Shinrin-yoku or forest-based interventions. Although 27 studies investigated the association between forest-based environments and health, only 5 looked at levels of street trees and tree canopy, with mixed results. It remains to be seen if the health benefits associated with forest bathing can be replicated in an urban environment by increasing street greenery and urban greenspace. Research in this field may inform national guidelines on the recommended number of trees necessary in urban and deprived areas to convey health benefits to the local populations.

A strength of this review is that all papers underwent rigorous critical appraisal using one of two carefully chosen tools; the Lachowycz and Jones checklist (Lachowycz and Jones, 2011) for observational studies and the Hanson and Jones and Ogilvie et al. risk of bias tool (Hanson and Jones, 2015, Ogilvie et al., 2007) for intervention studies. Both tools were tailored for the purposes of this review and every study underwent quality appraisal by two reviewers, with a high level of inter-rater agreement. However, 58.3% of the observational studies and 77% of the interventional studies scored ≥9 out of 11 in their respective quality appraisal tools. This limited heterogeneity in study quality may suggest that the tools may not have been sensitive enough to capture certain aspects of quality of the studies reviewed and differentiate between studies. Sensitivity analysis was conducted using only high quality studies (studies scoring ≥9). This cut-off point was chosen priori to balance the need to retain some studies with a need to understand how sensitive the results were to the inclusion of weaker studies. A limitation of this cut off point is that it implied that all quality appraisal criteria were of equal value, which may not be the case. Results remained consistent for heart rate and systolic blood pressure, however self-reported good health had a reduced effect size and lost statistical significance, with the drop in statistical significance being possibly explained by the lower power of this sub-analysis. Furthermore, the self-reported good health meta-analysis had an I^2^ of 100%, indicating a high risk of statistical heterogeneity. This result should therefore be interpreted cautiously.

A limitation of this review is that the search was restricted to manuscripts published in the English language. Furthermore, several health outcomes were only investigated in one or two studies, limiting comparability of results, for example, for respiratory mortality and various cancers. There were many differences between study populations; for example the largest and smallest study populations were >63 million (Wheeler et al., 2015) and 9 participants (Ochiai et al., 2015) respectively. The exclusion of mental health and communicable disease outcomes, whilst done pragmatically, is also a limitation of this review.

One key area for further research is how health professionals and policymakers might encourage patients to increase their exposure or even time spent in green spaces, and in particular to target those from lower SES areas. A number of included studies in this review reported a stronger relationship between greenspace and health outcomes for participants who were from low SES neighbourhoods, had lowest education levels, or those who were from areas with the lowest surrounding neighbourhood greenness. However, results were often not presented according to SES, meaning that formal subgroup analysis by SES level was not possible. Therefore it is not known if this may be the case for other health outcomes. Evidence has shown increased odds of higher psychosocial distress in residents of low SES areas (Kessler, 1982). Our meta-analysis results suggest that greenspace exposure may reduce salivary cortisol, a physiological marker of stress. Further studies investigating greenspace and heath but with a focus on SES groups and subsequent health inequalities are required to fill this gap in the literature.

From the quality appraisal, it was evident that there were two criteria recurrently missing from both observational and intervention studies. For the 103 studies assessed using the observational study quality checklist (Lachowycz and Jones, 2011) (Table 2), these were “*5. Did the green space measure include information on type of greenspace?”* and “*6. Use of greenspace was measured and included in the analysis”*. For the 40 intervention studies assessed using the risk of bias tool (Hanson and Jones, 2015, Ogilvie et al., 2007) ( Table 3), these were “*5. Did the authors show that there was no evidence of a concurrent intervention which could have influenced the results?”* and “*6. Were the study samples shown to be representative of the study population?*” Future research should take this into consideration, with observational studies aiming to include data on type of greenspace under investigation and the participants’ use of greenspace. Intervention studies should also aim to report on whether a concurrent intervention is in place, as well as commenting on the representativeness of the population.

Although this systematic review has uncovered a large body of research on the relationship between greenspace and health, there is a paucity of literature on the mechanisms underlying this relationship. Currently there are several suggested hypotheses. Greenspaces offer opportunities for physical activity, social cohesion, and stress reduction (Hartig et al., 2014), which each carry their own numerous health benefits. Exposure to the diverse variety of bacteria present in natural areas may convey immunoregulatory benefits and reduce inflammation (Rook, 2013). Much of the literature on forest bathing suggests that phytoncides (volatile organic compounds with antibacterial properties) released by trees may explain the salutogenic properties of shinrin yoku (Li et al., 2009, Tsunetsugu et al., 2010). Further research should build on the findings of this systematic review by hypothesising and testing the potential mechanisms underlying the relationship between greenspace and health. The associations between greenspace and mental health outcomes and communicable diseases, both outcomes that were not considered here, should also be explored further.

## 5 Conclusions

This review suggests that greenspace exposure is associated with wide ranging health benefits, with meta-analyses results showing statistically significant associations with reduced diastolic blood pressure, heart rate, salivary cortisol, incidence of type II diabetes and stroke, all-cause and cardiovascular mortality, as well as health-denoting associations with pregnancy outcomes, HRV, and HDL cholesterol, and self-reported health. However some meta-analyses results are limited by poor study quality and high levels of heterogeneity and should therefore be interpreted with caution. Increased greenspace exposure was also associated with non-pooled outcomes including neurological outcomes, respiratory mortality, and increased sleep duration. The findings of this systematic review suggest that the creation, regeneration and maintenance of accessible greenspaces and street greenery may form part of a multi-faceted approach to improve a wide range of health outcomes.
