# Supplementary material for: The health benefits of the great outdoors: A systematic review and meta-analysis of greenspace exposure and health outcomes
Source: Environ Res. 2018 Oct;166:628–37. doi: 10.1016/j.envres.2018.06.030 (PMC6562165; doi:10.1016/j.envres.2018.06.030)
Supplement: Supplementary file 1 — Supplementary material [file mmc1.docx]

| **Lead author, year, location** | **Study aim** | **Study design** | **Participant characteristics (n)** | **Health outcomes measured** | **Health outcome assessment tool or dataset** | **Greenspace measurement dataset or study setting** | **Treatment for confounding** | **Arrows showing direction of association, 95% CI of significant results where stated** |
| --- | --- | --- | --- | --- | --- | --- | --- | --- |
| Agay-Shay 2014, Israel  (Agay-Shay et al. 2014) | To evaluate the associations between proximity to green spaces and surrounding greenness and pregnancy outcomes | Cohort study | 39,132 singleton live births from a registry birth cohort in Tel Aviv during 2000-2006  (n = 39,132) | Birth weight (Including low and very low), gestational age and preterm deliveries/very preterm deliveries | National birth registry, Department of Mother and Child Health, Public Health Service of Israel | Residential surrounding greenness, Normalised Difference Vegetation Index, (NDVI), Landsat Enhanced Thematic Mapper + | Adjusted logistic regression models for infant’s gender, infant’s religion (Jewish/non-Jewish), maternal age, maternal marital status, maternal origin, year of birth and season of conception. Gestational age also adjusted for in birth weight analyses | Birth weight ↑  250m buffer NDVI: 19.2g (95% CI 13.3, 25.1)  Proximity to major green spaces (5000m^2^):  18.1g (95% CI 8.7, 27.6)  Low birth weight ↓  250m buffer NDVI: OR 0.84 (95% CI 0.78,0.90)  Proximity to major green spaces (5000m^2^):  OR 0.89 (95% CI 0.8, 0.99)  Very low birth weight ↘  Preterm delivery ↗  Very preterm delivery ↘  *Stronger association for low SES participants* |
| Agyemang 2007, The Netherlands  (Agyemang et al. 2007) | To investigate associations between neighbourhood-level environmental stressors (crime, housing density, nuisance from alcohol and drug misuse, quality of green space and social participation), and blood pressure (BP) and hypertension among different ethnic groups. | Cross-sectional study | Individual data from the Amsterdam Health Survey 2004, sample consisted of 517 Dutch, 404 Turkish, 365 Moroccans living in 15 neighbourhoods in Amsterdam.  Sample taken from representative population  (n = 1,286) | Blood pressure, hypertension | Primary measurement | Self-reported neighbourhood stressors from the Living in Amsterdam Survey 2003, Amsterdam Living and Security Survey 2004, The Social State of Amsterdam City Survey 2004 | Adjusted for potential confounding factors; age, sex, education level and BMI | Systolic blood pressure ↓ -4.92 (95% CI -9.21,-0.64) *Moroccan ethnic group only, Dutch and Turkish* ↘  Diastolic blood pressure ↘  Hypertension ↓ 0.61 (95% CI 0.36, 0.99) *Moroccan ethnic group only, Dutch and Turkish* ↘ |
| Andrusaityte 2016, Lithuania  (Andrusaityte et al. 2016) | To investigate the associations between surrounding greenness levels and asthma among children, and to explore a possible change of this association by the distance of the residence to a city park | Nested case-control study | 4-6 year old children of the KANC newborns cohort study  (n = 1,489) | Doctor-diagnosed asthma | International Study of Asthma and Allergies in Childhood 9ISAAC) questionnaire | Residential surrounding greenness, Normalised Difference Vegetation Index, (NDVI) as well as distance to the nearest city park | ORs adjusted for individual-level mother’s age at childbirth, maternal education, parental asthma, maternal smoking during pregnancy, breastfeeding, antibiotic use during the first year of life, keeping a cat during the past 12 months, living in a flat and yearly mean of ambient PM2.5 and NO_2_ | Asthma ↑  IQR increase in NDVI-100: 1.43 (1.10, 1.85) |
| Arbillaga-Etxarri 2016, Spain  (Arbillaga-Etxarri et al. 2016) | To validate the trail’s design by assessing the physiological response to unsupervised walking trails of 1) different intensities in COPD patients, and 2) same intensity from different public spaces in healthy adults | Case control study | 10 stable COPD patients (9 men, average age 67 ±9 years) and 10 healthy patients (5 men, average age 31 ±4 years)  (n = 20) | VO_2_, VCO_2_, respiratory exchange ratio (RER), min ventilation (VE), heart rate (HR), energy expenditure volume (MET-min), walking time, walking speed, steps, time for breaks, final dyspnea, final leg fatigue | Primary measurement and bespoke questionnaire | Park walk vs boulevard walk vs beach | Matching of participants | VO2, VCO2, respiratory exchange ratio (RER), min ventilation (VE), heart rate (HR), energy expenditure volume (MET-min), walking time, walking speed, steps, time for breaks, final dyspnea, final leg fatigue ↔ |
| Astell-Burt 2014a, Australia  (Astell-Burt et al. 2014a) | Associations between incidence of melanoma or non-melanoma skin cancer and neighbourhood greenspace in the 45 and Up Study | Cross-sectional study | 45 And Up Study, residents of New South Wales of 45 years and older. Randomly sampled from Australian universal health insurance database  (n = 267,072) | Self-reported medically diagnosed skin cancer (melanoma and non-melanoma) | 45 and Up Study bespoke questionnaire | Percentage greenspace within 1km buffer of home, Australian Bureau of Statistics (ABS) Meshblock 2006 classification | Models adjusted for measures of susceptibility (skin colour and tanning), socioeconomic variables, demographic and cultural characteristics (e.g. ancestry and country of birth) | Skin cancer ↑  When compared with <20% neighbourhood greenspace, odds of having non-melanoma skin cancer were significantly higher: 21-40% OR 1.05 (95% CI 1.03, 1.08), 41-60% OR 1.13 (95% CI 1.04,1.14), 61-80% OR 1.13 (95% CI1.06, 1.20), >80% OR 1.07 (95% CI 1.01, 1.14) |
| Astell-Burt 2013b, Australia  (Astell-Burt et al. 2013) | To investigate whether neighbourhood greenspace was associated with a healthier duration of sleep (to the nearest hour) in the 45 and Up Study | Cross-sectional study | 45 And Up Study, residents of New South Wales of 45 years and older. Randomly sampled from Australian universal health insurance database. Particpants completed baseline questionnaire between 2006 and 2009  (n = 259,319) | Sleep duration | 45 and Up Study bespoke questionnaire | Percentage greenspace within a 1km buffer around the census collection district (CCD), Australian Bureau of Statistics (ABS) Meshblock 2006 classification | Models adjusted for psychological distress, physical activity, and a range of demographic and socioeconomic characteristics | Sleep duration ↑  Risk of short sleep: >80% greenspace RR 0.86 (95%CI 0.81, 0.92) for 6-7 hours sleep and RR 0.68 (95% CI 0.57, 0.80) |
| Astell-Burt 2014b, Australia  (Astell-Burt et al. 2014b) | Investigate association between neighbourhood greenspace and the risk of T2DM in a large group of adult Australians | Cross-sectional study | 45 And Up Study, residents of New South Wales of 45 years and older. Randomly sampled from Australian universal health insurance database  (n = 267,072) | Medically diagnosed T2DM | 45 and Up Study bespoke questionnaire | Percentage greenspace within 1km buffer of home, Australian Bureau of Statistics (ABS) Meshblock 2006 classification | Odds ratios controlled for measures of demographic, cultural health diet, active lifestyles, socioeconomic status, and neighbourhood circumstances | Risk of type II diabetes ↓  41-60% GS: OR: 0.87, (95% CI 0.83, 0.92)  61-80% GS: OR 0.90, (95% CI 0.83, 0.97)  >80% GS: OR 0.90 (95% CI 0.82, 0.99) |
| Beil 2013, Finland  (Beil and Hanes 2013) | Investigate the effect of 4 urban environments on physiological and psychological stress measures | Pre-post study | Recruited from local community, average age 42.3 years (range 20-61 years), homogenous ‘non-hispanic white’ racial/ethnic background. 8 male, 7 female  (n = 15) | Salivary cortisol and alpha-amylase, self-reported measures of stress | Primary measurement | ‘Very natural’, ‘mostly natural’, ‘mostly built’ and ‘very built’ | Not specified | Salivary amylase ↓  Salivary cortisol ↘ |
| Besenyi 2014, USA  (Besenyi et al. 2014) | To examine the spatial relationship between park availability and prevalence of chronic health conditions (CHCs) across adult age groups | Cross-sectional study | Participants who responded to a questionnaire as part of an initial cluster random sample of residential addresses. Mean age 51.7, 38.8% male, 61.2% female  (n = 583) | Chronic health conditions (CHCs): Presence of heart problems (heart disease/BP/MI), cancer, diabetes, osteoporosis, depression/MH, asthma/allergies, disability, other | Bespoke questionnaire | Park availability within half mile of home, Kansas City Missouri (KCMO) Planning Department | All analyses controlled for gender, race/ethnicity, BMI, and household income | Chronic health conditions ↓  40-59 age group without a park within one half mile from home, likelihood to have 2 or more CHCs: OR 2.28 (1.05, 4.94) |
| Bijnens 2015, Belgium  (Bijnens et al. 2015) | To investigate the association between placental telomere length in twins and residential traffic exposure as well as semi-natural, forested, agricultural, residential and industrial areas within a 5000m radius from the residential address | Prospective study | Twins of Caucasian origin born between 1975 and 1982 who participated in a prenatal programming study selected from the East Flanders Prospective Twin Survey (EFPTS). Mean maternal age 27.5 years (range 19-40)  (n = 211) | Placental telomere length | Primary measure | Semi-natural, forested, agricultural areas in a 5000m buffer, Corine landcover 2000 | Covariates were selected a priori including newborn’s sex, gestational age, birth weight, birth year (linear and quadratic), zygosity and chorionicity, maternal age, SES indicators, (maternal education and neighbourhood household income) and smoking during pregnancy | Placental telomere length ↑  An IQR increase (22%) in maternal residential surrounding greenness (5km buffer) associated with an increase of 3.62% (95% CI 0.20, 7.15%) |
| Bixby 2015, England  (Bixby et al. 2015) | To assess whether local associations between greenspace and physiological and psychological health are transferable to a larger scale | Ecological cross-sectional study | Populations of the 50 largest cities in England  (n = not specified) | Risk of death from all causes, cardiovascular disease, lung cancer and suicide between 2002 and 2009 | Individual-level mortality records, UK Small Area Health Statistics Unit | Proportion of city area covered by green land, Land Cover Map 2007 | Adjusted for age, income deprivation and air pollution | All cause mortality ↘  Cardiovascular disease mortality ↘  Lung cancer ↗  Suicide mortality ↗ |
| Bodicoat 2014, UK  (Bodicoat et al. 2014) | To investigate the relationship between neighbourhood greenspace and type 2 diabetes | Cross-sectional study | 6,200 from general population, 4,276 from high-risk population. Mean age 59 years (range: 20-75 years). 47% female, 21% non-white ethnicity  (n = 10,476) | Screen-detected type 2 diabetes | Primary measurement | Percentage neighbourhood greenspace, Land Cover Map 2007 | Adjusted for ethnicity, age, sex, area social deprivation score and urban/rural status for increasing quartiles of neighbourhood greenspace, as well as BMI, physical activity, fasting glucose, 2h glucose and cholesterol for highest vs lowest quartile | Type II diabetes ↓  ORs for screen detected type 2 diabetes were 0.97 (0.80, 1.17), 0.78 (0.62, 0.98) and 0.67 (0.49, 0.93) for increasing quartiles of greenspace compared to the least green quartile after adjusting for confounders. |
| Botticello 2015, USA  (Botticello et al. 2015) | To assess the association between characteristics of the built environment and differences in perceived health among persons with spinal cord injury (SCI) using objective measures of the local community derived from GIS data | Secondary analysis of cross-sectional survey data | Spinal Cord Injury Model Systems (SCIMS) database participants, mean age 44.5 (±16.5) years, 80.5% male  (n = 503) | Perceived health | Bespoke questionnaire | Percentage neighbourhood open space, dataset not specified | ORs adjusted for demographic, impairment and community socioeconomic differences | Perceived health ↑ |
| Brown 2016, USA  (Brown et al. 2016) | To examine the association between objective measures of block-level greenness (vegetative presence) and chronic medical conditions, including cardiometabolic conditions | Retrospective cohort study | Medicare beneficiaries, 76.33 (±7.5) years, 58.33% female  (n = 249,405) | Number of chronic conditions (out of 27) | U.S. Centres for Medicare and Medicaid Services (CMS)’ Master Beneficiary Summary File | Mean Normalised Difference Vegetation Index (NDVI) for all Miami-Dade County Census blocks | Not specified | Increase in mean NDVI:  Total no. of chronic conditions ↓  Diabetes ↓  Hypertension ↓  Hyperlipidaemia ↓ |
| Burkart 2016, Portugal  (Burkart et al. 2016) | To investigate the influence of urban vegetation and water bodies on heat-related excess mortality in the elderly >65 years old in Lisbon | Ecological study | Inhabitants of civil parishes in the Lisbon Metropolitan Area from 1998 to 2008  (n = not specified) | Heat-related excess mortality in the elderly | National mortality records | Amount and spatial distribution of urban green quantified using Normalised Difference Vegetation Index (NDVI) | Models adjusted for time trend, average daily mean PM_10_ and O_3_ concentrations, percentage of the parish >65 years, building density, % college graduates and proportion of inhabitants receiving social benefits | With increasing NDVI quartiles:  Heat-related excess mortality in the elderly ↓ |
| Calogiuri 2016, Norway  (Calogiuri et al. 2016) | To investigate the impact of a green exercise intervention on psychological and physiological indicators of stress in municipality employees | RCT | Municipality employees, 49 (±8) years, 50% female  (n = 14) | Potential for restoration, affective state, blood pressure, cortisol awakening response and cortisol serum levels | Primary measurement | Green/nature area vs indoor exercise setting | Not specified | Cortisol awakening response (improved)  Diastolic BP ↓  BP ↔  Serum cortisol ↔ |
| Casey 2016, USA  (Casey et al. 2016) | To evaluate associations between prenatal residential greenness and birth outcomes across a range of community types | Cross-sectional study | Mothers from two hospitals who delivered between 2006 and 2013  (n = 20,569 delivery events and 20,598 neonates) | Term birth weight, small for gestational age birth, preterm birth and low 5 min Apgar score | Hospital records | Residential surrounding greenness, Normalised Difference Vegetation Index (NDVI) | Adjusted models controlled for neonate sex, year and season of birth, maternal age at delivery, maternal race/ethnicity, primary care status, smoking status during pregnancy, pre-pregnancy BMI, parity, receipt of Medical Assistance, number of antibiotic orders during pregnancy, distance to nearest major road, drinking water source within 20km of the home, exposure to swine operations, block group walkability and CSD quartiles | Higher greenness in cities:  Preterm birth ↓ OR 0.78 (95% CI 0.61, 0.99)  Small for gestational age birth ↓ OR 0.73 (95% CI 0.58, 0.97)  Birth weight ↔  Apgar score ↔ |
| Chum 2015, Canada  (Chum and O’Campo 2015) | To combine multiple neighbourhood influences in an integrated approach to understand the association between the built and social environment and and cardiovascular diseases (CVDs) | Cross-sectional study | Cross-sectional survey across 87 census tracts in Toronto. Mean age 44 years, 53% female  (n = 2,411) | Cardiovascular disease risk | Bespoke questionnaire | Percentage of local area used for parks, CanMap geo-database | Model 3 adjusted for individual level socio-demographic risk factors and health behaviours, model 4 is further adjusted for BMI and physical activity | Cardiovascular disease ↗ |
| Coutts 2010, USA  (Coutts et al. 2010) | To examine the relationships neyween the presence and accessibility of greenspace and county-level mortality in the state of Florida | Cross-sectional ecological study | Data on all-cause mortality and mortality from major cardiovascular diseases in 2007 were obtained from the Florida Department of Health’s Community Health Assessment Resource Tool Set (CHARTS).  (n = 167,708 deaths from all-causes, 54,542 deaths from cardiovascular diseases) | All-cause and cardiovascular mortality | State mortality database | Greenness in census tracts, 2009 Public land file | Controlled for the proportion of the population in each county that are overweight or obese, the proportion who smoke, the proportion of people who report being moderately physically active, the % of the population 65 and older, and % of the population with a bachelor’s degree or higher. Also controlled for ethnicity | All-cause mortality ↓  Cardiovascular mortality ↓ |
| Coutts 2015, USA  (Coutts and Horner 2015) | To determine if green space proximity to one’s residential location at time of death was predictive of all-cause premature mortality | Cross-sectional study | Death certificate records obtained from the Florida State Department of Health, Bureau of Epidemiology for the years 2000-2012  (n = 2,216,641) | Premature mortality from all causes | State mortality databases | Distance from residential address to nearest greenspace, amount of greenspace within a set of defined distances from each residence; 2009 public land file | Four separate models for males and four separate models for females, controlling for education, race, Hispanic ethnicity, and marital status | Years of potential life lost ↓ with decreasing distance to nearest greenspace for both males and females |
| Cusack 2017, USA  (Cusack et al. 2017) | To examine residential greenness and birth outcomes in Texas with large regional variation in greenness levels and a diverse population | Population-based cohort study | All births in Texas from 2000 to 2009  (n = 3,413,787) | Birth weight, odds of preterm birth, odds of being small for gestational age | Texas Vital Statistics program | Estimates of residential greenspace derived from MODIS satellite NDVI imagery | Adjusted for maternal and parental (if available) covariates age, smoking, education, race/ethnicity; pregnancy-related variables included method of delivery, parity, prenatal care, gestational age, baby’s sex, month and year of birth, as well as neighbourhood variables, NO_A_ air pollution concentrations and population density | Birth weight, odds of preterm birth, odds of being small for gestational age ↔  *Associations became non-significant in fully adjusted models* |
| Dadvand 2012a, Spain  (Dadvand et al. 2012a) | To investigate the association between surrounding greenness and birth weight, head circumference and gestational age at delivery | Cohort study | Singleton live births from INfancia y Medio Ambiente (INMA Project); four Spanish cohorts between 2003-8. Pregnant women ≥16 years old, recruited in first trimester  (n = 2,393) | Birth weight, gestational age and head circumference | Primary measurement | Residential surrounding greenness, Normalised Difference Vegetation Index (NDVI) | All analyses were adjusted for maternal age (continuous), ethnicity (white/other), socioeconomic status, education level, smoking, alcohol consumption, parity, infant sex (male/female), and season of conception. Birth weight analyses also adjusted for gestational age at delivery, maternal pre-gestational BMI, weight gain during pregnancy and paternal BMI. Analyses of head circumference further adjusted for gestational age at delivery, maternal height and paternal BMI | Birth weight ↑ 44.2g (95% CI 20.2, 68.2)  Head circumference ↑ 1.7mm (95% CI 0.5, 2.9)  Gestational age ↘ |
| Dadvand 2012b, Spain  (Dadvand et al. 2012b) | To investigate the effects of surrounding greenness and proximity to major green spaces on birth weight and gestational age at delivery and to describe the effect of socioeconomic position (SEP) on these relationships | Cohort study | Cohort of births in a Barcelona hospital between 2001-5  (n = 8,246) | Birth weight, gestational age at delivery and the effect of socioeconomic position on these relationships | Primary measurement | Residential surrounding greenness, Normalised Difference Vegetation Index, (NDVI) | Birth weight analysis: Adjusted for gestational age at delivery, neighbourhood SEP, degree of urbanisation, distance of residential place to major roads, maternal weight, age, ethnicity, academic level, occupation, smoking, alcohol consumption, parity, history of obs/gynae pathologies, use of assisted reproductive technologies  Gestational age analysis: Adjusted for neighbourhood SEP, degree of urbanisation, distance of residential place to major roads, maternal age, ethnicity, academic level, occupation, smoking, alcohol consumption, parity, history of preterm birth, history of obs/gynae pathologies, use of assisted reproductive technologies and sex of infant | Birth weight ↑  *Beneficial association only amongst the lowest education level group who had higher surrounding NDVI (Regression coefficient: 436.3 (95% CI 43.1, 829.5)) or lived close to a major green space (Regression coefficient: 189.8 (95% CI 23.9, 355.7))*  Gestational age ↔ |
| Dadvand 2014, Spain  (Dadvand et al. 2014) | To evaluate health benefits and risks associated with different types of greenness in children, in terms of sedentary behaviour (excessive screen time), obesity, current asthma and allergic rhinoconjunctivitis | Cross-sectional study | Population-based sample of schoolchildren (9-12 years old) in Spain in 2006  (n = 3,178) | Sedentary behaviour, obesity, current asthma and allergic rhinoconjuncitivitis | Bespoke questionnaire | Residential surrounding greenness, Normalised Difference Vegetation Index, (NDVI)  Residential proximity to greenspaces, Urban Atlas map (2007) | All analyses adjusted for indicators of individual SES, parental education, parental school (public/private), area-level SES at census tract level. Respiratory and allergic outcomes further adjusted for child’s sex and age, exposure to tobacco smoke at home, having older siblings, parental history of asthma. Analyses of overweight/obesity adjusted for sport activity at school or sport facilities and having siblings. Sedentary behaviour analyses controlled for child’s sex and age and having siblings | Asthma ↗  Allergic rhinoconjunctivitis ↗ |
| Dadvand 2015, Spain  (Dadvand et al. 2015) | To assess the association between exposure to greenspace and measures of cognitive development in primary schoolchildren | Cohort study | Conducted as part of the brain development and air pollution ultrafine particles in schoolchildren (BREATHE) project. Mean age 8.5 years at baseline, 50% female  (n = 2,593) | Developmental trajectory of working memory, superior working memory, and inattentiveness | Primary measurement | Residential and school surrounding greenness, Normalised Difference Vegetation Index (NDVI) | Model adjusted for age, sex and SES indicators at individual and area levels | Cognitive development ↑  Pre-post results for greenspace within and surrounding school grounds show significant improvements in working memory, superior working memory and a significant reduction in inattentiveness |
| Dadvand 2016, Spain  (Dadvand et al. 2016) | To assess the association between greenness exposure and subjective general health (SGH), and to evaluate mental health status, social support and physical activity as mediators of this association | Cross-sectional study | Population-based randomised sample of adults residing in Barcelona  (n = 3,461) | SGH, mental health | SGH: bespoke questionnaire  Mental health: GHQ-12 | Residential surrounding greenness as well as objective and subjective (perceived) proximity to greenspace;  Normalised Difference Vegetation Index (NDVI), Parks and Garden Map of Barcelona, bespoke questionnaire | Randomised sample  Models further adjusted a priori for age, sex and indicators of SES at both individual and area levels | SGH ↑  100m buffer OR 1.17 (95% CI 1.05, 1.31) 250m buffer OR 1.18 (95% CI 1.06, 1.32) 500m buffer OR 1.16 (95% CI 1.05, 1.29)  Subjective proximity to greenspace OR 1.36 (95% CI 1.11, 1.67) |
| Dalton 2016, UK  (Dalton et al. 2016) | To investigate the association between neighbourhood greenspace and the occurrence of incident diabetes over time | Prospective cohort study | EPIC Norfolk cohort study  (n = 23,865) | Incident type 2 diabetes | Self-report of doctor diagnosed diabetes/questionnaire/ self-report of diabetes-specific medication | Percentage neighbourhood greenspace, Land Cover Map 2007 | Analysis adjusted for socio-economic status at both the individual and neighbourhood level | Diabetes ↓  HR 0.81 (95% CI 0.65, 0.99) for individuals living in the greenest quartiles |
| de Jong 2012, Sweden  (de Jong et al. 2012) | To assess how perceived green neighbourhood qualities were associated with three self-reported indicators of well-being (neighbourhood satisfaction, physical activity and general health) | Cross-sectional study | Questionnaire sent to 52,142 participants randomly selected from the population registry. 55% female  (n = 24,847) | Three self-reported indicators of well-being: neighbourhood satisfaction, physical activity and general health | Public health survey data | Bespoke objective measurement of greenspace quality  Availability of green qualities within 300m of residential address, CORINE land use data | Analyses were adjusted for possible confounding by sex, age, highest level of education, economic difficulties, country of origin and type of residence. Stratified analysis on neighbourhood satisfaction for type of residence | Self-reported health ↑ |
| Demoury 2016, Canada  (Demoury et al. 2017) | To assess whether living in the proximity of greener areas was related to prostate cancer risk | Population-based case-control study | Men younger than 76 years in the Montreal area  (n = 1933 cases, 1994 controls) | Newly diagnosed with primary PCa | The Prostate Cancer & Environment Study (PROtEuS) | Residential greenspace at 2 time points, Landsat TM5 Normalised Difference Vegetation Index (NDVI) | Model 1: adjusted for age  Model 2: adjusted for age, ancestry, first-degree family history of PCa, education, reported family income, marital status, smoking, alcohol consumption, dietary habits and a history of diabetes  Model 3: Further adjusted for neighbourhood material and social deprivation | Prostate cancer ↓  300m buffer, an IQR increase of 0.11 in recruitment OR 0.82 (95% CI 0.74, 0.92)  10 years previous: OR 0.86 (95% CI 0.74, 1.00)  Remained significant for all buffer sizes at recruitment time point |
| de Vries 2003, The Netherlands  (De Vries et al. 2003) | To investigate the association between neighbourhood greenspace and self-reported health | Multilevel analysis (cross-sectional) | Health interview survey among random samples of practice populations of 103 general practices in the Netherlands  (n = 10,179) | Number of symptoms experienced in the last 14 days, perceived general health, Dutch general health questionnaire (GHQ) | The Dutch National Survey of Morbidity and Interventions in General Practice | Percentage green and blue space in living environment; National Land Cover Classification, presence of a garden (yes/no) | The scores on all variables at the (semi-)interval level had been centred (but not standardised) | Self-reported health ↑ |
| Donovan 2011, USA  (Donovan et al. 2011) | To investigate whether tree canopy cover is associated with reduced risk of poor birth outcomes in Portland, Oregon | Cohort study | All singleton live births in Portland, Oregon during 2006-7, where the mothers’ address was a single family home. Mean age 30.3 years  (n = 5,696) | Preterm birth, gestational age of less than 37 weeks, small for gestational age, birth weight below the 10th percentile for gestational age and gender | Birth certificate data, Portland, Oregon | Percentage tree canopy in 50, 100, and 200m buffers surrounding residential address; Metro land cover classification 2007 | To ensure that all confounders were included, any covariate with significant variation in canopy cover within 50m of a house that was not selected for retention during thr backward selection process was re-introduced to the final model. If any re-introduced variable caused a 10% or greater change in any coefficients of interest, it was retained in the final model. None of the covariates evaluated met this threshold | Small for gestational age births ↓  10% increase in tree canopy cover within 50m of a house reduced the number of small for gestational age births by 1.42 per 1000 births (95% CI -0.11, -2.72) |
| Droomers 2016, The Netherlands  (Droomers et al. 2016) | To investigate the impact of real-life changes in the quality and quantity of green space in severely deprived neighbourhood on physical activity and perceived general health | Quasi-experimental study | Dutch National Health Interview Survey from 2004 to 2011  (n = 48,132) | Perceived general health | Dutch National Health Interview Survey from 2004 to 2011 | Local greenspaces that underwent improvement interventions | All analyses adjusted for age, sex, household composition, ethnicity, education and standardised disposable household income; additionally adjusted for overall intensity of District Approach | Perceived general health ↔ |
| Dunstan 2013, Wales  (Dunstan et al. 2013) | To determine the association between self-reported general health and an objectively assessed measure of the residential environment. (Using the Residential Environment Assessment Tool, REAT) | Cohort study | Caerphilly Prospective Study, taken from individual census records, 47.78% male  (n = 31,442) | Self-reported general health | UK census data | Residential environment assessment tool (REAT) | Models fitted adding individual-level covariates: age, gender, marital status, housing tenure and employment status | Self-reported health ↘ |
| Fjortoft 2004, Norway  (Fjørtoft 2004) | To investigate the relationship between children’s motor development and playing in a natural environment | Quasi-experimental study (randomised) | Experimental and control groups selected from voluntary kindergartens with the same original playground opportunities in the same geographic area. Age range 5-7 years old  (n = not specified) | Motor fitness, balance, co-ordination | Primary measurement | Forest playground vs traditional outdoor playground | Not stated | Motor development ↑ *in children* |
| Fuertes 2014, Germany  (Fuertes et al. 2014) | To examine whether residential greenness is associated with childhood doctor diagnosed allergic rhinitis, eyes and nose symptoms | Cohort study | GINIplus and LISAplus birth cohorts  (n = 5,803) | Childhood doctor-diagnosed allergic rhinitis, eyes and nose symptoms and aeroallergen sensitisation. Also air pollution data (stratified analysis) | Bespoke questionnaire | Mean residential greenness in a 500m buffer around the 10 year home address; Normalised Difference Vegetation Index, (NDVI) | Models were adjusted for age, sex, parental history of atopy, older siblings, maternal smoking during pregnancy, tobacco smoke exposure in the home (birth-4 years), cohort and parental education | Eye and nose symptoms ↓  GINI/LISA South (urban) OR 1.15 (95% CI 1.01, 1.31)  GINI/LISA North (rural) OR 0.71 (95% CI 0.56, 0.89)  Allergic rhinitis ↓  GINI/LISA North (rural) OR 0.75 (95% CI 0.60, 0.93) |
| Gong 2014, UK  (Gong et al. 2014) | To explore the role of neighbourhood greenspace in determining levels of participation in physical activity among elderly men with different levels of lower extremity function | Prospective study | Caerphilly Prospective Study  (n = 1,010) | Lower extremity "physical function"; i.e. physical activity, Psychological health general stress | Bespoke questionnaire and General Health Questionnaire (GHQ-30) | Quantity and variation of neighbourhood greenspace; Normalised Difference Vegetation Index (NDVI) | Model 2 adjusted for lower extremity physical function, psychological distress, general health, car ownership, age group, marital status, social class and education level | Lower extremity physical function ↑  OR 1.92 (95% CI 1.12, 3.28) |
| Grazuleviciene 2014a, Lithuania  (Grazuleviciene et al. 2014a) | To investigate the effect of proximity to city parks on blood pressure categories during the first trimester of pregnancy | Cross sectional study | Pregnant women recruited to the European Commission’s FP6 HiWATE project between 2007-9. 20-45 years old  (n = 3,416) | Blood pressure in first trimester of pregnancy | Primary measurement | Distance to nearest park; unspecified land cover dataset | Models adjusted for age, education, socioeconomic position, passive smoking, BMI, chronic disease, parity and stress | Blood pressure ↓  >1000m green space distance odds ratio for increased blood pressure:  OR 1.74 (95%CI 1.14,2.66) |
| Grazuleviciene 2015b, Lithuania  (Grazuleviciene et al. 2015b) | To investigate the effect of walking in a city park vs. an urban environment on coronary artery disease (CAD) patients haemodynamic parameters | Intervention study | Male and female Kaunas city residents, 62.3 ± 12.6 years of age with CAD  (n = 20) | Haemodynamic parameters of CAD patients, including SBP/DBP, HR, exercise duration and HR recovery | Questionnaire, Primary measurement | Pine park vs urban busy street | Randomisation of participants | Heart rate ↓  Diastolic blood pressure ↓  Heart rate recovery ↓  Exercise duration ↑ |
| Grazuleviciene 2015a, Lithuania  (Grazuleviciene et al. 2015a) | To investigate whether surrounding greenness levels and/or distance to city parks affect birth outcomes | Cohort study | Kaunas birth cohort, participants recruited between 2007-9 in the early stages of pregnancy. Age range 20 to 45 years old  (n = 3,292) | Gestational age, preterm birth, birth weight, low birth weight, term low birth weight, and small for gestational age | Birth certificate data, Kaunas, Lithuania | Residential surrounding greenness, distance to nearest park; Normalised Difference Vegetation Index, (NDVI) | Low birth weight models adjusted for maternal marital status, education, smoking, blood pressure, BMI, parity, chronic diseases, previous preterm birth, paternal smoking and infant sex  Term low birth weight adjusted for maternal marital status, education, smoking, alcohol consumption, BMI, blood pressure, parity, paternal smoking and infant sex  Preterm birth adjusted for maternal marital status, education, smoking, renal diseases, stress, previous preterm birth, parity and paternal smoking  Small for gestational age adjusted for maternal age, marital status, education, social status, smoking, BMI, parity and previous preterm birth  Birth weight adjusted for maternal height, smoking, marital status, BMI, diabetes and chronic health diseases, parity  Gestational age adjusted for maternal marital status, education, smoking, renal diseases, stress, parity, previous preterm birth and paternal smoking | For subjects with low surrounding greenness and >1000m to the nearest park:  Low birth weight↑ OR 2.23(95%CI 1.20,4.15)  Term low birth weight ↑ OR 2.97 (95% CI 1.04, 8.45)  Preterm birth ↑ OR 1.77 (95% CI 1.10, 2.81)  Lower gestational age↑  Beneficial park effect on foetal growth in environment with least surrounding greenness ↗ |
| Grazuleviciene 2016, Lithuania  (Grazuleviciene et al. 2016) | To examine the effects of restorative walking in a park vs in an urban environment on coronary artery disease (CAD) patients stress parameters and cardiac function | RCT | Male and female Kaunas city residents with CAD, mean age 62.3 ±12.6 years  (n = 20) | Heart rate, blood pressure, stress levels, mood | Primary measurement, Positive and Negative Effect Schedule (PANAS) mood score evaluation | Park vs urban environment | Participants randomly assigned to study arm | Salivary cortisol ↓  Blood pressure ↓ |
| Grigsby-Toussaint 2015, USA  (Grigsby-Toussaint et al. 2015) | To determine whether exposure to attributes of the natural environment (e.g. greenspace) attenuates the likelihood of reporting insufficient sleep among US adults. | Cross-sectional study | 2020 Behavioural Risk Factor Surveillance System (BRFSS), a yearly, randomised telephone survey of behavioural risk factors among US adults ≥18 years of age. 64.13% female. Average age 56.6 years.  (n = 255,171) | Self-reported sleep insufficiency | 2010 Behavioural Risk Surveillance System (BRFSS) survey | County-level greenspace; Normalised Difference Vegetation Index, (NDVI) | Adjusted for age, gender, marital status, race, education, employment status, number of children, physical activity, smoking, income level, asthma, general health status, emotional support, disability, BMI category and heavy alcohol use | Sleep quality ↑  Individuals reporting 7-13 days or 21-29 days of insufficient sleep.  7-13 days OR 0.995 (95% 0.988, 1.002)  21-29 days OR 0.991 (95% CI 0.986, 0.9996)  Lower odds of exposure to natural amenities were observed for individuals reporting 21-29 days of insufficient sleep  OR 0.843 (95% CI 0.747, 0.951) |
| Gutierrez-Zornoza 2014, Spain  (Gutiérrez-Zornoza et al. 2014) | To examine (a) whether distance from home to school is a determinant of active commuting to school (ACS), (b) the relationship between distance from home to heavily used facilities (school, green spaces and sports facilities) and the weight status and cardiometabolic risk categories and (c) whether ACS has a positive impact on schoolchildren’s health. | Cross-sectional study | Cross-sectional study of the final measurements taken in a cluster randomized trial to evaluate the effectiveness of leisure-time physical activity on preventing childhood obesity (the MOVI programme). Schoolchildren aged 10-12 years old. 49.37% male, average age 11 years.  (n = 956) | BMI and fat mass, blood pressure, fasting plasma lipid profile, insulin, fitness, physical activity and active commuting to school (ACS) | Primary measurement | Distance from home to greenspace; National Plan for Aerial Orthophotography 2007 | Model 1 controlled for age, fat mass percentage, and fitness according to age  Model 2 controlled for controlled for age, commuting, fitness according to gender  Model 3 adjusted for age and cardiovascular fitness; for the MetS index, adjusted for age, cardiovascular fitness and fat mass; for cardiovascular fitness adjusted for age and fat mass | Cardiometabolic risk ↔ |
| Hartig 2003, USA  (Hartig et al. 2003) | To compare “restoration” in natural and urban field settings. | RCT | A group of normotensive students mean age 20.8 years (SD 3.7), 50% female and 97% non-smokers.  (n = 112) | Systolic and diastolic blood pressure, emotional states | Primary measurement, Zuckerman’s Inventory of Personal Reactions (ZIPERS) | Green vs urban environment | Participants randomly assigned to study arm | Systolic blood pressure ↘  Diastolic blood pressure ↓ |
| Hoehner 2013, USA  (Hoehner et al. 2013) | To examine the associations of built environment features around the home and workplace with cardiorespiratory fitness (CRF) based on a treadmill test and BMI. | Cross-sectional study | The Cooper Centre Longitudinal Study. 70.9% male.  (n = 8,857) | Cardiorespiratory fitness | Primary measurement | Neighbourhood greenspace in buffers surrounding residential address. Dataset unspecified. | Adjusted for age, sex, education, race, marital status, presence of children in the home, cigarette smoking, BMI, and all other built environment variables for the respective location of interest as well as weekly MET-minutes of physical activity | Cardiorespiratory fitness ↗ |
| Hu 2008, USA  (Hu et al. 2008) | To examine if there is association of stroke with air pollution, income and greenness in northwest Florida | Ecological geographical study | Stroke death count data at the census tract level was obtained  (n = not specified) | Stroke mortality rates | State mortality records (Florida CHARTS) | Self-reported frequency of visits to greenspace, residential neighbourhood and work buffer; Landsat 7 Enhanced Thematic Mapper Plus (ETM+) | Calculated standardized mortality rates | Stroke mortality ↓  95% Credible set (-0.289, -0.031) |
| Hystad 2014, USA  (Hystad et al. 2014) | To investigate associations between residential greenness and birth outcomes and evaluate the influence of spatially correlated built environment factors on these associations | Cohort study | All births between 1999-2002 in the metropolitan area of Vancouver  (n = 64,705) | Birth weight, preterm deliveries, gestational age | National birth registry | Residential surrounding greenness; Normalised Difference Vegetation Index (NDVI) | Analyses adjusted for month and year of birth, infant sex, first nations status, parity, maternal age, maternal smoking during pregnancy as well as maternal education and income quintiles | Birth weight ↑  An interquartile increase in greenness (0.1 in residential NDVI) associated with higher term birth weight: 20.6g (95% CI 16.5, 24.7)  Small for gestational age ↓OR 0.97 (0.94, 1.00)  Very preterm birth ↓ OR 0.91 (0.77, 1.07)  Moderately preterm birth ↓ OR 0.95 90.91, 0.99) |
| James 2016, USA  (James et al. 2016) | To examine the prospective association between residential greenness and mortality | Cohort study | U.S.-based Nurses Health Study (NHS), female registered nurses from 11 states in 1976  (n = 121,701) | Mortality rate and cause-specific mortality | National Death Index | Residential surrounding greenness; Normalised Difference Vegetation Index (NDVI) | Examined the following covariated as potential confounders, effect modifiers or mediators: fixed ethnicity/race, smoking status, fixed individual-level SES, area-level SES, weight status, region, urbanicity, whether a participant had changed addresses during follow-up, physical activity, air pollution, social engagement and mental health | Mortality ↓  Highest greenness quintile (Q5) in 250m buffer:  HR 0.88 (95% CI 0.82, 0.94)  Cancer ↓ Q5 HR 0.87 (95% CI 0.78, 0.97)  Respiratory ↓ Q5 HR 0.66 (95% CI 0.52, 0.84)  Stroke ↓ Q2 HR 0.76 (0.59, 0.97) |
| Jia 2016, China  (Jia et al. 2016) | To determine the health benefits of forest bathing trips on elderly patiends with chronic obstructive pulmonary disease (COPD) | RCT | COPD patients from Hangzhou  (n = 20) | Flow cytometry, ELISA and profile of mood states (POMS) | Primary measurement | Forest vs city settings | Not specified | Perforin ↓  Granzyme B expression ↓  Pro-inflammatory cytokines ↓  Stress hormones ↓ |
| Jonker 2014, The Netherlands  (Jonker et al. 2014) | To investigate the impact of three different measures of urban green on small-area life expectancy (LE) and healthy life expectancy (HLE) in The Netherlands | Cohort study | Standard 5 year abridged table data for the estimation of male and female LE and HLE for neighbourhoods in all 22 metropolitan agglomerations in neighbourhoods in The Netherlands in the 2006-2009 period were obtained  (n = minimum required population size of 1,750 person years, exact population not specified) | Small-area life expectancy (LE) and healthy life-expectancy (HLE) | Life and healthy life expectancy estimates, Statistics Netherlands | % greenspace in neighbourhood; Dutch Land Use Database 2008 (BBG), average distance (km) to nearest public green; Statistics Netherlands, self-reported measure of greenspace quality; bespoke questionnaire | Standardized coefficients used | Life expectancy ↑  An increase in 1 SD in % urban greenspace is associated with a 0.1 year higher LE.  An increase in 1 SD of quality of greenspace is associated with approximately 0.3-year higher LE and HLE.  Average distance to public green is unrelated to population health |
| Kabisch 2016, Germany  (Kabisch et al. 2016) | To analyse the potential intra-urban relationships between childrens’ health determinants, in particular deficits in viso-motoric development, and outcomes and natural areas in Berlin | Ecological study | Population of Berlin  (n = 3,562,166) | Deficits in viso-motoric development in children | Berlin’s Senate Department for Health and Social Issues | % natural area, per capita natural area, availability of natural area; Local land use data from Berlin’s Senate Department of Urban Development and the Environment | Not specified | Low % natural areas ↑ deficits in viso-motoric development in children |
| Kardan 2015, Canada  (Kardan et al. 2015) | To examine the association between tree canopy density beside the streets and in other areas such as parks and domestic gardens with an individual’s health. The health variables focused on are 1) overall health perception, 2) presence of cardio-metabolic conditions, 3) mental health problems | Cohort study | Subset of the Ontario Health Study. 59% female, mean age 43.8 years (range 18-99)  (n = 31,109) | Self-reports of general health perception, cardio-metabolic conditions and mental illnesses | Ontario Health Study questionnaire | Toronto Street Tree General Data and GIS Forest and Land Cover | None specified | Self-reported health ↑  Cardiometabolic conditions ↓ |
| Kihal-Talantikite 2013, France  (Kihal-Talantikite et al. 2013) | To investigate the relationship between green spaces and the spatial distribution of infant mortality taking account neighbourhood deprivation levels | Ecological study | Prevalence of infant death in the Lyon metropolitan area over the study period 2000-2009  (n = 1,340,155 population of Lyon metropolitan area) | Neonatal mortality | Equit’Area project municipality mortality records, National Institute for Statistics and Economic Studies | Spatial land cover datasets for Lyon Metropolitan area | Stage 1: Unadjusted  Stage 2: Adjusted for greenness level or socioeconomic neighbourhood (deprivation index)  Stage 3: Adjusted for greenness level and deprivation index at the neighbourhood level including the interaction between the two variables | Neonatal mortality ↓ |
| Kim 2015, South Korea  (Kim et al. 2015) | To assess the feasibility of forest therapy as an adjuvant to enhance natural cytotoxicity. | Feasibility study | Volunteer women aged 25-60 years with stage III breast cancer. All subjects exposed to daily forest therapy for 14 days. Mean age 56 years.  (n = 11) | Natural killer cell population, perforin and granzyme B levels | Primary measurement | Forest | Matching of participants | Natural killer cell population ↑  MD 125.3 (95% CI 43.1, 207.4)  Level of perforin ↗ MD 128.1 (-28.4, 284.5)  Level of granzyme B ↗ MD 6.7 (-2.8, 16.3) |
| Kim 2016, South Korea  (Kim et al. 2016) | To investigate the association between parks and green areas and hyperlipidaemia in adults | Cross-sectional study | Adults participating in the 2009 Korean Community Health Survey (KCHS)  (n = 212,584) | Hyperlipidaemia | Bespoke questionnaire | Parks and green areas per capita in 2009 using data from the Korean Statistical Information Service | Models adjusted for age, sex, marital status, education, monthly income, jobs, smoking status, alcohol drinking, a history of diabetes mellitus, BMI, self-reporting stress and moderate physical activity | Hyperlipidaemia ↓  Lowest greenspace quartile - diagnosed hyperlipidaemia: OR 1.23 (95% CI 1.17, 1.29)  Lowest greenspace quartile - treatment of hyperlipidaemia: OR 1.45 (95% CI 1.35, 1.56) |
| Lachowycz 2014, England  (Lachowycz and Jones 2014) | To examine the relationship between greenspace access, walking and mortality | Cross-sectional study | Individual level data sourced from the Active People Survey (APS). 60% female, mean age 55.0 years  (n = 165,424) | Premature mortality from circulatory disease | Standardised mortality ratios | Access to greenspace; Generalized Land Use Data 2005 dataset (GLUD) | Model 1: Unadjusted  Model 2: Adjusted for individual level covariates: age, gender, ethnicity, social class, car ownership, month of data collection  Model 3: Further adjusted for MSOA-level environmental variables: index of multiple deprivation, urban-rural classification, population density | Cardiovascular mortality ↓  *Tests for mediation found no evidence to suggest that recreational walking explained the relationship greenspace and mortality* |
| Larson 2016, USA  (Larson et al. 2016) | To evaluate the relationship between urban park quantity, quality and accessibility and aggregate self-reported wellbeing scores | Ecological study | 2014 data from 44 U.S. cities  (n = 44 cities ranging in size from New York, NY 8.175,136 to Wichita KS 382,373, exact population not specified) | Physical wellbeing | Gallup-Healthways Well-being Index (WBI) | Trust of Public Land’s (TPL) Park Score Index | Controlled for a range of potential geographical and socioeconomic correlates | Physical wellbeing ↑ with park quantity  ↗ with park quality and park accessibility |
| Laurent 2013, USA  (Laurent et al. 2013) | To study the relationship between greenspace exposure and 3 pregnancy outcomes; birth weight in term born infants, preterm deliveries and preeclampsia | Cohort study | Neonatal records from 1997-2006 were extracted from a perinatal research database of four hospitals in California  (n = 81,186) | Birth weight, preterm deliveries and preeclampsia | Hospital database | Residential surrounding greenness; Normalised Difference Vegetation Index (NDVI) | Models adjusted for maternal age, poverty, length of gestation, maternal race/ethnicity, insurance status, parity, infant’s gender (birth weight analysis only), pyelonephritis (preterm birth analysis only) and diabetes (preeclampsia and birth weight analyses only) | Birth weight ↑  Increase in birth weight with a 1 IQR increase in greenspace in 50m buffer:  6.22g (95% CI 3.22, 9.22)  Preterm birth ↓OR 0.984 (95% CI 0.961, 1.007)  Preeclampsia ↔ |
| Lee 2011, Japan  (Lee et al. 2011) | To provide scientific evidence supporting the efficacy of forest bathing as a natural therapy by investigating its physiological benefits using biological indicators in outdoor settings | RCT | Young male Japanese adults recruited from local universities. Mean age 21.2 years  (n = 12) | Heart rate variability, LF/HF ratio in R-R interval variability (parasympathetic and sympathetic nervous system activity), cortisol levels, pulse rate, systolic blood pressure, diastolic blood pressure | Primary measurement  Self-reported psychological measures | Forest | Matching of participants | Parasympathetic activity ↑  Sympathetic activity ↓  Heart rate↓  Salivary cortisol ↓ |
| Lee 2014a, South Korea  (Lee et al. 2014a) | Investigating the health benefits of forest walking on cardiovascular reactivity | RCT | Young Japanese adult males. Mean age 21.2 years  (n = 48) | Blood pressure, heart rate, heart rate variability | Primary measurement | Forest vs urban | Matching of participants | Parasympathetic activity ↑  Sympathetic activity ↓  Heart rate ↓ |
| Lee 2014b, South Korea  (Lee and Lee 2014b) | To investigate the acute effects of forest walking on arterial stiffness and pulmonary function in Korean elderly women | RCT | Recruited by advertisement at a senior welfare centre. Participants were all female. Average age of city walking group (n=19) 71.1 years, average age for forest walking group (n=43) 70.2 years  (n = 62) | Blood pressure, arterial stiffness (CAV1), pulmonary function (FEV1, FEV) | Primary measurement | Forest/city walking intervention | Matching of participants | Blood pressure ↓  CAVI ↓  FEV1 ↑  FEV6 ↑ |
| Li 2008a, Japan  (Li et al. 2008a) | To investigate the effect of a forest bathing trip on human NK activity in female subjects | Intervention study | Healthy nurses aged 25-43 years, selected with informed consent  (n = 13) | Blood and urine sampled for: Nk activity, numbers of NK and T cells, granulysin, perforin, granzymes A/B-expressing lymphotcytes, estradiol and progesterone concentration in serum, Adrenaline and noradrenaline concentration in urine. Phytoncides were also measured in the forest | Primary measurement | Forest | Matching of participants | Natural killer cell population and activity ↑  Perforin ↑  Granulysin ↑  Granzymes A/B expressing lymphotcytes ↑ |
| Li 2008b, Japan  (Li et al. 2008b) | To investigate the effect of forest bathing on NK activity compared with a trip to a city, and to measure how long the effect on NK activity lasts | Intervention study | Twelve healthy male subjects aged 35-56 years  (n = 12) | Blood and urine sampled for: Natural killer cell (NK) activity, numbers of NK and T cells, granulysin, perforin, granzymes A/B-expressing lymphotcytes, adrenaline concentration in urine. Phytoncides were also measured in the forest | Primary measurement | Forest | Matching of participants | Natural killer cell population and activity ↑  Level of perforin ↑  Granulysin ↑  Granzymes A/B expressing lymphotcytes ↑  Adrenaline concentration in urine ↓ |
| Li 2009, Japan  (Li et al. 2009) | To investigate the effects of a day trip to a forest park on human NK activity in forest parks | Intervention study | Healthy male subjects aged between 35-53 years  (n = 12) | Blood and urine sampled for: Natural killer cell (Nk) activity, numbers of NK and T cells, granulysin, cortisol (blood), perforin, granzymes A/B-expressing lymphotcytes, adrenaline concentration in urine. Phytoncides were also measured in the forest | Primary measurement | Forest | Matching of participants | Natural killer cell activity ↑  CD16+ and CD56+ natural killer cell population ↑  Perforin ↑  Granulysin ↑  Granzyme A/B expressing NK cells ↑  Blood cortisol ↑  Urinary adrenaline ↑  CD4+ cells ↓ |
| Li 2011, Japan  (Li et al. 2011) | To investigate the effects of walking under forest environments on cardiovascular and metabolic parameters | Intervention study | Healthy male subjects, mean age 57.4 years (range 36-77 years)  (n = 16) | Blood pressure, urinary noradrenaline, dopamine, serum adiponectin, dehydroepiandosterone sulfate (DHEA-S) levels, serum N-terminal pro B-type natriuretic peptide (NT-proBNP) and urinary dopamine | Primary measurement | Forest | Matching of participants | Serum adiponectin ↑  DHEA-S level ↑  Blood pressure ↓  Noradrenaline ↓  Dopamine NT-pro-BNP↓  Urinary dopamine ↓ |
| Li 2016, Japan  (Li et al. 2016) | To investigate the effects of forest bathing on cardiovascular and metabolic parameters | Intervention study | Middle-aged male subjects with high-normal blood pressure or hypertension who were not taking antihypertensive drugs, 51.2 ±8.8 years (range 40-69 years)  (n = 19) | Blood pressure, heart rate, blood analysis (serum triglycerides, total cholesterol, LDL, HDL, remnant-like particles, adiponectin, blood glucose, insulin level, DHEA-S, hs-CRP) urinary adrenaline, noradrenaline and dopamine, POMS score | Primary measurement | Forest vs urban environment | Matching of participants | Heart rate ↓  Urinary noradrenaline ↓  Adiponectin ↑  Blood pressure, urinary adrenaline, urinary dopamine, other metabolic parameters ↔ |
| Lovasi 2008, USA  (Lovasi et al. 2008) | To describe the direction and magnitude of any association between street trees and childhood asthma | Ecological cross-sectional study | Asthma prevalence for 4-5 year old children in 1999 and asthma hospitalisations among children <15 years in 1997 as assessed by NYC Dept of Health  (n = not specified) | Asthma prevalence among children aged 4-5 years old and asthma hospitalisations among children less than 15 years old | School asthma screening, hospitalisation for asthma | 1995 New York street tree census | Models controlled for population density, demographic and socioeconomic characteristics (percent poverty, percent African American, and percent Latino), and proximity to pollution sources | Asthma prevalence ↓  A 1 SD (343 trees/km2) increase in street tree density associated with a 24% lower asthma prevalence: RR 0.74 (95% CI 0.62, 0.87)  Asthma hospitalisation ↘ |
| Lovasi 2013, USA  (Lovasi et al. 2013) | To investigate the association of tree canopy cover with subsequent development of childhood asthma, wheeze, rhinitis, and allergic sensitization | Cohort study | The CCCEH birth cohort in NYC. Pregnant women recruited through prenatal clinics. Dominican or African-American children born in 1998-2006 and living in economically disadvantaged areas of NYC  (n = 549) | Childhood asthma, wheeze, rhinitis, and allegic sensitisation | Brief Respiratory Questionnaire (BRQ), International Study of Asthma and Allergies in Childhood (ISAAC) questionnaire | 2010 New York Tree Canopy Data (Mac Faden et al. 2012); surrounding prenatal address | Covariates included sex, age at time of outcome measurement, ethnicity, maternal asthma, previous birth, other previous pregnancy, Medicaid enrolment, tobacco smoke in the home, active maternal smoking, and the following characteristics of 0.25km buffers: population density, percent poverty, percent park land, and estimated traffic volume | Asthma ↑  Significant positive association of tree canopy coverage with diagnosed asthma at 7 years of age consistent with a 17% increase in the prevalence of asthma with each SD increase in tree canopy coverage.  RR: 1.17 (95%CI 1.02, 1.33)  Allergic sensitisation ↑  IgE antibody response to the tree pollen mix ↑ RR 1.43 (95% CI 1.19, 1.72)  IgE antibody response to any of the 9 allergens ↑ RR 1.20 (95% CI 1.05, 1.37) |
| Maas 2006, The Netherlands  (Maas et al. 2006) | To investigate the strength of the relationship between the amount of green space in people’s living environment and their perceived general health | Cross-sectional study | Representative of the Dutch population in terms of age, gender and health insurance type  (n = 250,782) | Perceived general health | The second Dutch national survey of general practice (DNSGP-2) | % neighbourhood greenspace; National Land Cover Database (LGN4) | Controlled for urbanity, sociodemographic and socioeconomic characteristics | Self-reported health ↑ |
| Maas 2008, The Netherlands  (Maas et al. 2008) | To investigate whether physical activity mediates the relationship between neighbourhood greenspace and self-perceived health | Multilevel analysis, cross-sectional study | A subset of the second Dutch national survey of general practice (DNSGP-2). Representative of the Dutch population in terms of age, gender and health insurance type, 54.4% female  (n = 4,899) | Perceived general health | The second Dutch national survey of general practice (DNSGP-2) | % neighbourhood greenspace; National Land Cover Database (LGN4) | Controlled for urbanity, sociodemographic and socioeconomic characteristics | Self-reported health ↑ |
| Maas 2009a, The Netherlands  (Maas et al. 2009a) | To explore whether social contacts are an underlying mechanism behind the relationship between green space and health | Multi-level analysis, cross sectional study | The second Dutch national survey of general practice (DNSGP-2). Representative of the Dutch population in terms of age, gender and health insurance type, 54.9% female  (n = 10,089) | Self-reported health indicators: perceived general health, number of health complaints, self-rated propensity to psychiatric morbidity | The second Dutch national survey of general practice (DNSGP-2) | % neighbourhood greenspace; National Land Cover Database (LGN4) | Controlled for age, gender, household size, level of education, income and urbanicity | Self-reported health ↑ |
| Maas 2009b, The Netherlands  (Maas et al. 2009b) | To investigate whether physician-assessed morbidity is also related to green space in people’s living environment | Cross-sectional study | The second Dutch national survey of general practice (DNSGP-2). Representative of the Dutch population in terms of age, gender and health insurance type  (n = 345,143) | Morbidity data on physical and mental health | National mortality records | % neighbourhood greenspace; National Land Cover Database (LGN4) | Controlled for urbanity, demographic and socioeconomic characteristics | Morbidity ↓  Annual prevalence rates of 24 disease clusters for people who have 10% more green space than average, for 1km radius:  High BP ↓ OR 0.99 (95% CI 0.98, 1.00)  Cardiac disease ↓ OR 0.98 (95% CI 0.97, 0.99)  CHD ↓ OR 0.97 (95% CI 0.95, 0.99) p<0.01  Stroke, brain haemorrhage ↓ OR 0.98 (95% CI 0.95, 1.00)  Neck and back complaints ↓ OR 0.98 (95% CI 0.97, 0.99) p<0.01  Severe back complaints ↓ OR 0.98 (95% CI 0.97, 0.99) p<0.01  Severe neck and shoulder complaints ↓ OR 0.98 (95% CI 0.97, 0.99) p<0.01  Severe elbow, wrist and hand complaints ↓ OR 0.97 (95% CI 0.96, 0.98) p<0.01  Upper respiratory tract infection ↓ OR 0.97 (95% CI 0.96, 0.98) p<0.01  Bronchitis/pneumonia ↓ OR 0.97 (95% CI 0.97, 1.00)  Asthma, COPD ↓ OR 0.97 (95% CI 0.96, 0.98) p<0.01  Migraine/severe headache ↓ OR 0.98 (95% CI 0.97, 0.99) p<0.01  Vertigo ↓ OR 0.97 (95% CI 0.95, 0.99) p<0.01  Severe intestinal complaints ↓ OR 0.98 (95% CI 0.96, 1.00)  Infectious disease of the intestinal canal ↓ OR 0.97 (95% CI 0.95, 0.99) p<0.01  MUPS ↓ OR 0.97 (95% CI 0.96, 0.98) p<0.01  Chronic eczema ↓ OR 0.99 (95% CI 0.97, 1.00)  Acute urinary tract infection ↓ OR 0.97 (95% CI 0.96, 0.98) p<0.01  Diabetes mellitus ↓ OR 0.98 (95% CI 0.97, 0.99) p<0.01 |
| Mao 2012a, China  (Mao et al. 2012a) | To provide scientific evidence supporting the efficacy of forest bathing as a natural therapy for human hypertension. | Intervention study | Hypertension patients, randomly divided into 2 groups of 12.  (n = 24) | BP, cardiovascular disease-related pathological factors including endothelin-1, homocysteine, renin, angiotensin, angiotensin II, angiotensin II type 1 recentor, angiotensin II type 2 receptor, inflammatory cytokines interleukin-6 and TNF alpha. Mood states (POMS), airquality. | Primary measurement  POMS, air quality | Forest | Randomisation | Blood pressure ↓  Bioindicators ↓ |
| Mao 2012b, China  (Mao et al. 2012b) | To investigate the effects of short-term forest bathing on human health. | Intervention study | Twenty healthy male university students, randomly divided into 2 groups of 10.  (n = 20) | BMI, SBP, DBP HR, IL-6, TNF-alpha, T-SOD, MDA, ET-1, Cortisol, testosterone, T-cell, B-cell, Thylymphocyte, tslymphocyte, NK cell, CD4/CD8, Platelet activation (CD42a, CD14) | Primary measurement | Forest | Randomisation | TNF-alpha ↓  IL6 ↓  C-reactive protein ↓  High-sensitivity C-reactive protein ↓  MDA ↓  ET-1  Serum cortisol ↓  Leukocytes ↑  T-, T-helper cells, NK lymphocytes, T suppressor cells, testosterone levels ↗ |
| Markevych 2014, Germany  (Markevych et al. 2014) | To assess whether surrounding residential greenness is associated with blood pressure in 10 year old German children | Cross-sectional analysis | Based on two birth cohorts, GINIplus and LISAplus, recruited healthy full term neonates  (n = 2,078) | Blood pressure | Primary measurement | Residential surrounding greenness; Normalised Difference Vegetation Index (NDVI) | Models adjusted for study (GINIplus/LISAplus), sex, parental education, parental hypertension, child’s age (years), season of blood pressure measurements, BMI of each child at at 10 years old | Blood pressure ↓ |
| Markevych 2016, Germany  (Markevych et al. 2016) | To investigate the association between residential greenness and blood lipids in children | Longitudinal analysis | Based on two birth cohorts, GINIplus and LISAplus, recruited healthy full term neonates, 10 and 15 year follow ups  (n = 1,552) | Blood lipids | Primary measurement | Residential surrounding greenness; Normalised Difference Vegetation Index (NDVI) | All models adjusted for exact age at time of blood lipid measurement, sex, study (GINIplus/LISAplus), study areaparental education, fasting status and BMI; models additionally adjusted for weekly physical activity, puberty category, and area-level SES | Blood lipids ↔ |
| Matsunaga 2011, Japan  (Matsunaga et al. 2011) | To investigate the association between a hospital rooftop garden and physiological relaxation (HRV) on elderly people requiring care | Cross-sectional, within subject study | Elderly women requiring help walking, without dementia or pacemakers, mean age 81.7 years  (n = 30) | Heart rate variability (HRV) | Primary measurement | Hospital garden | Matching of participants | Heart rate variability ↓ |
| McCracken 2016, UK  (McCracken et al. 2016) | To examine the health-related quality of life of children in relation to quality and use of greenspace | Cross-sectional study | Children at participating schools aged 8-11, mean age 9.7 ±9 years, 55.6% female  (n = 276) | Health-related quality of life, self-reported | Kid-KINDL questionnaire | Residential greenspace: Central Scotland Green Network  Use of urban greenspace: bespoke questionnaire | Additional demographic information collected included: page, gender, number of siblings, type of home, and presence of a garden | Health related quality of life ↑ |
| Mitchell 2007, England  (Mitchell and Popham 2007) | To determine the association between the percentage of greenspace in an area and the standardised rate of self-reported “not good” health, and to explore whether this association holds for areas exhibiting different combinations of urbanity and income deprivation. | Cross-sectional, ecological study | Respondents to the 2001 census who were asked whether their health was “good”, “fairly good” or “not good”.  (All residents in England as at the 2001 census, number not stated) | “Not good" health status | England 2001 census | Generalized Land Use Data 2005 dataset (GLUD) | Each model controlled for urban higher income, urban lower income, suburban higher income, suburban lower income, rural higher income and rural lower income (unless model was stratifying by characteristic) | Self-reported health ↗ |
| Mitchell 2008, England  (Mitchell and Popham 2008) | To investigate whether the magnitude of income-related health inequality varies by exposure to green space | Cross-sectional, ecological study | Anonymised individual mortality records. Populations older than retirement age were excluded as inequalities in mortality tend to be at a maximum in the working age population  (n = 40,813,236, with 366,348 deaths) | All-cause mortality, cause specific mortality (circulatory disease, lung cancer, and intentional self-harm) | Individual-level mortality records | Generalized Land Use Data 2005 dataset (GLUD) | All models adjusted for age group, sex, deprivation in education, skills and training, deprivation in living environment, population density and urban or rural classification | All-cause mortality ↓  Incidence rate ratio for all-cause mortality for the most income deprived quartile compared with the least deprived was 1.93 (95% CI 1.86, 2.01) in the least green areas, whereas it was 1.43 (95% CI 1.34, 1.53) in the most green areas  Cardiovascular mortality ↓  IRR 2.19 (95% CI 2.04, 2.34) least green  IRR 1.54 (95% CI 1.38, 1.73) most green  Intentional self harm ↔ |
| Mitchell 2010, UK  (Mitchell et al. 2011) | To investigate whether associations between greenspace exposure and health vary according to the origins of the green space indicator and, by proxy, the type of green spaces captured by the indicator | Ecological study | 286 small areas in four British cities (York, Exeter, Edinburgh and Glasgow). Each “small area” was a Census Area Statistic (CAS) ward  (n = 1,625,495) | Mortality and self-reported morbidity | Mortality records and census data | Coordination of information on the Environment (CORINE), British Ordnance Survey’s master map (OSMM), Generalized Land Use Data 2005 dataset (GLUD) | All models controlled for age and sex of the exposed populations | Self-reported health ↑  Mortality ↓ |
| Morita 2011, Japan  (Morita et al. 2011) | To study the non-temporary effects of successive walks in forested areas (shinrin-yoku) on hypertension prevalence and blood pressure levels | Cohort study | Results from the baseline survey of the Japan Multi-Institutional Collaborative Cohort (J-MICC) study, mean age 52.1 years, 68% male  (n = 4,666) | Blood pressure, hypertension | Primary measurement, bespoke questionnaire | Self-reported frequency of forest walking | Adjusted for age, BMI, smoking status, alcohol consumption and habitual exercise | Blood pressure ↔ |
| Nakau 2013, Japan  (Nakau et al. 2013) | To examine the effect of spiritual care of cancer patients by integrated medicine in a green environment | Pilot study – pre-post | Cancer patients, mean age 58.1 years, 18 females (mean age 56.6 years) and 4 males (mean age 65.3 years) with breast or lung cancer  (n = 22) | QOL questionnaire, spirituality, fatigue (cancer fatigue scale), psychological state and Natural Killer (NK) cell activity | Primary measurement, bespoke questionnaire | Forest | Matching of participants | Natural killer cell activity ↑  Cancer-associated fatigue ↓  Self-reported health ↑ |
| Ngom 2016, Canada  (Ngom et al. 2016) | To determine the role of proximity to specific types of greenspaces as well as their spatial location in the relationship with the most morbid cardiovascular diseases (CVD) and diabetes | Cross-sectional study | Sample of data from the Quebec Integrated Chronic Disease Surveillance System (QICDSS)  (n = 3,920,000) | Diabetes, ischaemic heart disease, cerebrovascular diseases, heart failure | Quebec Integrated Chronic Disease Surveillance System (QICDSS) | Nearest distance to several types of greenspace and the presence of vegetation in open areas using CanMap | Controlled for several social and environmental factors | Cerebrovascular prevalence ↓  Highest distance to greenspace with sports facilities: PRR 1.11 (95% CI 1.01, 1.22)  Diabetes ↓  Highest distance to greenspace with sports facilities: PRR 1.09 (95% CI 1.03, 1.13)  Heart failure ↔  Ischaemic heart disease ↔ |
| Ochiai 2015, Japan  (Ochiai et al. 2015) | To assess the physiological and psychological effects of forest therapy on middle aged males with high-normal blood pressure. | Pre-post study | Japanese males (mean age 56 years, range 40-72 years) with high-normal blood pressure  (n = 9) | Blood pressure, urinary adrenaline and serum cortisol (not salivary) | Primary measurement | Forest | Matching of participants | Blood pressure ↓  Urinary adrenaline ↓  Serum cortisol ↓ |
| Ohtsuka 1998, Japan  (Ohtsuka et al. 1998) | To investigate the effect of shinrin-yoku on blood glucose in diabetic patients | Pre-post study | 58 female and 29 male non-insulin dependent diabetic patients volunteered for the study, mean age 61 years  (n = 87) | Blood glucose (non-fasting) and HbA1c | Primary measurement | Forest | Not specified | Non-fasting blood glucose ↔  HbA1c ↔ |
| Padilla 2016, France  (Padilla et al. 2016) | To identify and describe how socioeconomic, health accessibility and exposure factors accumulate and interact in small areas in a French urban context, to assess environmental health inequalities related to infant and neonatal mortality | Ecological study | Population of Nice metropolitan area, France  (n = approximately 537,769) | Infant and neonatal mortality rate | Death certificate records | Proportion of geographic area occupied by greenspaces, Coordination of Information on the Environment (CORINE) | Not specified | Infant and neonatal mortality ↔ |
| Paquet 2014, Australia  (Paquet et al. 2014) | To investigate whether residential environment characteristics related to food, walkability and public open spaces were associated with incidence of four cardio-metabolic risk factors in a biomedical cohort | Cohort study | North West Adelaide Health Study (NWAHS), a longitudinal biomedical cohort. 52.4% female, mean age 51.5 years  (n = 3,145) | Pre-diabetes/diabetes, hypertension, dislipidaemia, abdominal obesity | Primary measurement | Road network distance to public open space (POS); defined by Normalised Difference Vegetation Index (NDVI) | Analyses accounted for spatial clustering, gender, age, household income, education, duration of follow up and area-level socio-economic deprivation | Diabetes ↔  Prediabetes ↔  Hypertension ↔  Dyslipidaemia ↔ |
| Park 2007, Japan  (Park et al. 2007) | To examine the physiological effects of shinrin yoku | RCT | Healthy male students, mean age 22.8 years  (n = 12) | Salivary cortisol and cerebral activity, sensory evaluation | Primary measurement, bespoke self-reported sensory evaluation | Forest | Matching of participants | Cerebral activity ↓  Salivary cortisol ↓ |
| Park 2009, Japan  (Park et al. 2009) | To examine the physiological effects of forest recreation on autonomic nervous activity. | RCT | Male university students, mean age 21.8 years.  (n = 12) | BP, HR, HRV | Primary measurement | Forest | Matching of participants | Blood pressure ↓  Heart rate ↓  Heart rate variability ↓ |
| Park 2010, Japan  (Park et al. 2010) | To review previous research on Shinrin yoku and present new results to clarify physiological effects. | Intervention study | 12 healthy male university students in 24 areas between 2005-6, 280 students in total. Mean age 21.7 years.  (n = 280) | Salivary cortisol, BP, HR, HRV | Primary measurement | Forest | Matching of participants | Blood pressure ↓  Heart rate ↓  Heart rate variability ↓  Salivary cortisol↓  Sympathetic nervous activity ↓  Parasympathetic nervous activity ↑ |
| Pasanen 2014, Finland  (Pasanen et al. 2014) | To investigate the relationship between perceived health and physical activity indoors, outdoors in built environments and outdoors in nature | Longitudinal survey | National survey data from Finland. 55.6% female, mean age 45.2 years (range 15-74 years)  (n = 2,070) | Perceived general health, emotional well-being and sleep quality | Bespoke questionnaire | Outdoor Recreation Demand Inventory (LVVI2), Finnish Forest Research Institute | Adjusted for covariates | Self-reported health ↑  Sleep quality ↗ |
| Pereira 2012, Australia  (Pereira et al. 2012) | To investigate the effect of neighbourhood greenness in relation to coronary heart disease risk | Cross-sectional study | Residents of the Perth metropolitan area. 59% female  (n = 11,404) | Coronary heart disease, stroke | Health and Wellbeing Survey, hospital records; Western Australian Department of Health | Residential surrounding greenness; Normalised Difference Vegetation Index (NDVI) | Adjusted for age, sex, possession of healthcare card, education, household income, non-gestational diabetes, BMI, hypertension, high cholesterol, daily fruit and vegetable intake, risky drinking behaviour, smoking and a proxy for air quality | Hospitalisation for heart disease or stroke ↓  OR 0.63 (95%CI 0.43, 0.92) among neighbourhoods with highly variable greenness (highest tertile) compared to those in predominantly green or predominantly non-green neighbourhoods |
| Picavet 2016, The Netherlands  (Picavet et al. 2016) | To explore the cross-sectional and longitudinal associations between greenspace and physical activity and several health indicators | Cross-sectional and longitudinal study | Doetinchem Cohort Study, adults aged 20-59  (n = 4,005) | Health-related quality of life, chronic diseases, blood pressure | Health-related quality of life measured by the RAND36 (similar to SF-36), chronic diseases self-reported | Percentage greenspace in the living environment and change in percentage green, National Land Cover Classification Database | All analyses adjusted for differences by age, sex and socioeconomic status | Systolic blood pressure ↓  1km radius green 0.40 (95% CI 0.15, 0.66)  Agricultural green 0.25 (95% CI 0.08, 0.43)  Urban green -0.40 (95% CI -0.74, -0.06)  Hypertension, diabetes, CVD, asthma complaints, COPD complaints ↔ |
| Piccolo 2015, USA  (Piccolo et al. 2015) | To identify and estimate the contribution of specific aspects of neighbourhoods that may be associated with racial/ethnic disparities in T2DM | Cohort study | A community-based random-sample survey, the Boston Area Community Health (BACH) survey III, from 3 racial/ethnic groups (black, Hispanic, and white). Mean age 55.89 years, 55.43% female  (n = 2,764) | Prevalent T2DM; fasting glucose <125mg/dL, HbA1c ≥ 6.5%, or self-report of a T2DM diagnosis | Primary measurement, Boston Area Community Health III Survey | Percentage recreational open space per census tract; Massachusetts Office of Geographic Information 2013 | Adjusted for demographic and socioeconomic variables | Type II diabetes ↔ |
| Pietila 2015, Finland  (Pietilä et al. 2015) | To examine how the presence of and access to green spaces is related to the level of physical activity and self-rated health | Cross-sectional study | Finnish Outdoor Recreation Demand Inventory (LVVI) survey data. Age range 15-74, 55.4% female  (n = 3,108) | Self-reported health | Bespoke questionnaire | Self-reported quality and availability of greenspace; Finnish National Outdoor Recreation Demand Inventory (LVVI) | Adjusted for age, gender, education and experience of an exceptional or difficult situation in life prior to the survey | Self-reported health ↔ |
| Putrik 2015, The Netherlands  (Putrik et al. 2015) | To explore associations between certain features of neighbourhood environment and self-rated health and depressive symptoms in Maastrict | Cross-sectional study | Survey data. Mean age 55 years, 52% female  (n = 9,879) | Self-rated health and presence of depressive symptoms | Bespoke survey | Neighbourhood environment characteristics; bespoke survey | Models adjusted for individual age, gender, education and income group | Self-reported health ↗ |
| Qin 2013, China  (Qin et al. 2013) | To investigate the influence of urban greenspaces on physiological status | Cross-sectional observational study | Visitors to the Shanghai Botanical Garden  (n = 64) | Heart rate variability; electroencephalogram (EEG), electrocardiogram (ECG) | Primary measurement of park visitors | Shanghai botanical gardens | None specified | Heart rate variability ↓ |
| Reklaitiene 2014, Lithuania  (Reklaitiene et al. 2014) | To assess the relationship between greenspace proximity, use of green space and depressive symptoms and perceived general health amongst a random sample | Cross-sectional study | Population-based sample in Kaunas, Lithuania. Age range 45-72, 54.7% female  (n = 6,944) | Health behaviours, depressive symptoms and poor and very poor perceived general health | Bespoke questionnaire, depressive systems assessed using CES-D10 scale | Distance to city park and park use; unspecified dataset | Analyses adjusted for age, marital status, education, smoking, use of alcohol and BMI | Self-reported health ↑  *Women only, non-significant for men* |
| Requia 2016, Brazil  (Requia et al. 2016) | To quantify the distance-decay cardiorespiratory diseases risk related to 28 neighbourhood aspects in the Federal District, Brazil | Cross-sectional study | Hospital admissions for cardiorespiratory disease in Brazil  (n = not specified) | Cardiorespiratory diseases risk | Brazilian National Health Database | Natural environment land use, Sedhab (2012) database | Not specified | Cardiorespiratory diseases risk ↓  1km^2^ increase in green areas intra urban was associated with reduced risk of hospital admission |
| Richardson 2010a, New Zealand  (Richardson et al. 2010a) | To investigate whether there is a socioeconomic gradient in green space exposure and whether green space exposure is associated with cause-specific mortality (CVD and lung cancer) | Ecological study | Anonymised individual-level mortality data for every registered death between 1996 and 2005 from the New Zealand Ministry of Health. Limited to adults under 65  (n = 1,546,405) | Risk of mortality from cardiovascular disease and from lung cancer | Individual-level mortality records, New Zealand Ministry of Health 2001 | Census area unit greenspace coverage; Department of Conservation (DOC) Conservation Boundaries data set (2003), Land Information New Zealand’s (LINZ) Core Records System (2004), Ministry for the Environment Land Cover Database 2 (LCDB2 2001) | Controlled for census level data on income, employment, communication, support, transport, qualifications, living space, home ownership, smoking, air pollution, and population density as a measure of urbanity | Cardiovascular mortality and respiratory mortality ↗ |
| Richardson 2010b, UK  (Richardson and Mitchell 2010b) | To examine the relationship between urban greenspace and health and to investigate gender differences in this relationship | Ecological cross-sectional study | Individual-level mortality records were obtained and matched to CAS wards with an estimate of green space coverage  (n = 28,600,000) | Cardiovascular disease mortality, respiratory disease mortality, self-reported limiting long-term illness | Individual-level mortality records, Office of National Statistics (England and Wales), General Register Office for Scotland, Northern Ireland Statistics and Research Agency (NISRA) | Generalised Land Use Database (GLUD) and Coordination of Information on the Environment (CORINE) | All models adjusted for age-group, income deprivation quartile, air pollution and country | Cardiovascular mortality ↓ *men only*  IRR 0.95 (95% CI 0.91, 0.98) greenest wards  Respiratory mortality ↓ *men only*  IRR 0.89 (95% CI 0.83, 0.96) greenest wards |
| Richardson 2011, USA  (Richardson et al. 2012) | To investigate whether a relationship between green space coverage and selected mortality rates exists at the city level in the USA | Ecological cross-sectional study | Populations of the 49 largest US cities  (n = 43,000,000) | City-level standardised rates of mortality from heart disease, diabetes, lung cancer, motor vehicle fatalities and all-causes | City-level standardised rates of mortality from various causes, 2004 | City-level greenspace coverage; National Land Cover Database (NCD, 2001) | Adjusted for socioeconomic characteristics, household income, ethnicity, air pollution, percentage of households without a car and sprawl index | Mortality from cardiovascular disease ↘, diabetes↘, lung cancer↘, automobile accidents ↘  All-cause mortality ↑ highest greenness level  Men 132.90 (95% CI 18.33, 247.46)  Women 94.21 (95% CI 21.76, 166.66) |
| Richardson 2013, New Zealand  (Richardson et al. 2013) | To investigate whether urban greenspace is related to individual-level health outcomes, and if physical activity is a mediating factor | Cross-sectional study | Respondents to the New Zealand Health Survey 2006/7, 56.5% female  (n = 8,157) | Cardiovascular disease, poor general health, poor mental health, overweight status | 2006/7 New Zealand Health Survey (NZHS) | Neighbourhood level greenspace availability; Department of Conservation’s Conservation Area Boundaries (2003), Land Information New Zealand’s Core Record System (2004) and the Ministry for Environment’s Land Cover Database (2001) | Adjusted for individual level covariates including sex, age group, smoking behaviour, and an index of individual socioeconomic deprivation | Cardiovascular disease risk ↘  Self-reported health ↘ |
| Roe 2013, UK  (Roe et al. 2013) | To investigate the relationship between greenspace and stress (perceived stress and salivary cortisol) in deprived urban communities | Cross-sectional study | Men and women aged 33-55 years of age from socio-economically deprived areas of Dundee. Mean age 44.75 years, 50% male  (n = 106) | Salivary cortisol levels and perceived stress | Primary measurement, perceived stress scale (PSS)  psychological stress measures | Percentage neighbourhood greenspace; Census Area Statistics Ward (CAS), Centre for Research on Environment Society and Health (CRESH) | Adjusted for access to a garden | Salivary cortisol ↓*women only* |
| Roe 2016, UK  (Roe et al. 2016) | To explore the relationship between general health and a range of individual, social and physical environmental predictors in deprived neighbourhoods | Multi-case study | Participants from 6 ethnic groups in 6 case-study locations (London: Hackney and Islington, West Midlands: Coventry and Wolverhampton, and Greater Manchester: Rochdale and Oldham)  (n = 523) | Self-reported general health | Ethnic Focus survey | Perceptions of local greenspace and self-reported use of local greenspace | Not specified | Self-reported general health ↑ *for poorest health group only* |
| Ruokolainen 2015, Estonia and Finland  (Ruokolainen et al. 2015) | To test the diversity hypothesis by analysing the relationship between land use around the home and atopic sensitisation in children | Cohort study | Four cohorts of children and adolescents 0.5-20 years  (n = 1,044) | Serum IgE specific to inhallant allergens, proteobacteria on the skin of healthy individuals. Prevalence of atopic sensitisation in children and adolescents aged 0.5-20 years | Primary measurement, DIABIMMUNE, LUKAS, KARA datasets | Percentage neighbourhood greenspace; Coordination of Information on the Environment (CORINE) | Adjusted for potential confounding factors | Atopic sensitisation ↓ *children* |
| Sbihi 2015, Canada  (Sbihi et al. 2015) | To investigate the effect of early-life exposure to surrounding residential greenness on asthma incidence | Cohort study | All 1999-2002 single births in the metropolitan area of Vancouver, British Columbia  (n = <65,000 children) | Asthma diagnosis | Physician billing and hospital discharge records | Residential surrounding greenness; Normalised Difference Vegetation Index (NDVI) | Sex and age matched to 5 randomly chosen controls; models adjusted for covariates including month/year of birth, sex, first nation status, as well as maternal parity, age, smoking during pregnancy and initiation of breastfeeding and assigned socioeconomic indicators | Asthma ↓ |
| Skarkova 2015, Czech Republic  (Skarková et al. 2015) | To assess the impact of the environment on asthma prevalence | Cross-sectional study | Representative sample of children aged 5, 9, 13 and 17 from the Czech Republic  (n = 13,456) | Asthma prevalence | National Institute of Public Health (NIPH) questionnaire survey | Land cover data; Fundamental Base of Geographic Data (ZABAGED) administered by the Czech Office for Surveying, Mapping and Cadastre | Adjusted regression coefficients calculated | Asthma prevalence:  ↓ with presence of natural forests  ↑ with agricultural land use |
| Song 2013, Japan  (Song et al. 2013) | To investigate the physiological and psychological effects of walking in urban parks in winter on young males | Controlled trial | Japanese male university students, mean age 22.5 years  (n = 13) | Heart rate, Heart rate variability (HRV), mood states | Primary measurement, Profile of Mood State (POMS) questionnaire, State-Trait Anxiety Inventory (STAI) | Walking intervention; urban park/city area | Matching of participants | Heart rate ↓  Heart rate variability ↓ |
| Song 2015a, Japan  (Song et al. 2015a) | To investigate the effect of forest walking on autonomic nervous system activity in middle aged hypertensive individuals | Intervention study | Japanese men, mean age 58.0 years  (n = 20) | Heart rate, Heart rate variability (HRV), relaxation questionnaire | Primary measurement, Profile of Mood State (POMS) questionnaire, modified semantic differential method (SD) | Walking intervention; forest/urban setting | Matching of participants | Heart rate ↓  Heart rate variability ↓ |
| Song 2015b, Japan  (Song et al. 2015b) | To clarify the physiological and psychological effects of walking in urban green areas | Intervention study | Japanese males, mean age 22.3 ±1.2 years  (n = 23) | Heart rate, heart rate variability, Profile of Mood States (POMS), State-Trait Anxiety Inventory | Primary measurement, POMS questionnaire, State-Trait Anxiety Inventory | Urban park vs city area | Matching of participants | Heart rate ↓  Sympathetic nervous activity ↓  Parasympathetic nervous activity ↑ |
| Stigsdotter 2010, Denmark  (Stigsdotter et al. 2010) | To investigate the associations between greenspace and health, health-related quality of life and stress | Cross-sectional study | 2005 Danish Health Interview Survey, age range 16-64 years  (n = 21,832) | Health-related quality of life and stress | Danish Institute of Public Health 2005 health interview survey, Short form health survey (SF-36) | Self-reported proximity to a greenspace; Danish Institute of Public Health 2005 health interview survey | Analyses adjusted for gender, age, cohabitation status, combined school and vocational education, accommodation type, size of municipality and ethnic background | Self-reported health ↑  >1km from a greenspace/natural area  OR 1.42 (95% CI 1.17, 1.73) |
| Sugaya 2011, Japan  (Sugaya et al. 2011) | To compare oxidative damage levels after urban walking and forest walking | Intervention study | Female patients with rheumatoid arthritis aged 48-62 years old  (n = 12) | serum hydroperoxide, MMP-3, uringary 8-OHdG, and salivary IgA | Primary measurement | Forest vs urban environment | Matching of participants | Urine 8-0 HdG levels, MMP-3, salivary IgA ↑  Serum hydroperoxide ↓ |
| Sugiyama 2008, Australia  (Sugiyama et al. 2008) | To examine associations of perceived neighbourhood greenness with perceived physical and mental health, and to investigate whether walking and social factors account for these relationships | Cross-sectional study | Observational epidemiological study, 63% female, mean age 45 years  (n = 1,895) | Physical and mental health scores | Short-form health survey (SF-12), physical component scores (PCS), mental component scores (MCS) | Perceived neighbourhood greenness; Neighbourhood Environment Walkability Scale | Model adjusted for age, education, work status, household income, marital status, and a further model also adjusted for walking for recreation, social coherence score and local social interaction | Self-reported health ↗ |
| Sugiyama 2009, UK  (Sugiyama et al. 2009) | To examine what aspects of neighbourhood open space are associated with walking for recreation and for transport by older people | Cross-sectional study | 60.8% female, mean age 75.0 years, 10% non-white  (n = 284) | Self-reported quality of life (QOL) and "health status" | Behavioural Risk Factor Surveillance Scheme (BRFSS), Satisfaction With Life Scale (SWLS) | Bespoke scale measuring quality of neighbourhood open space (NOS) | All models adjusted for participants’ age, functional capability and their level of educational attainment | Self-reported health ↗ |
| Sulander 2016, Finland  (Sulander et al. 2016) | To investigate the relationship between urban greenspace visits and mortality among adults | Cross-sectional study | 939 women; mean age 82 years, 456 men; mean age 81 years  (n = 1,395) | Mortality | National Population Information System survey | Frequency of visiting urban green areas, bespoke questionnaire | Model 1: Adjusted for sociodemographics and education  Model 2: Adjusted for sociodemographics and self-reported diseases  Model 3: Adjusted for sociodemographics, self-reported diseases and functional capacity | Mortality ↓  Visit a green area few times a year or less: HR 2.2 (95% CI 1.2, 4.1) *Model 3, but significant for all models* |
| Sung 2012, Japan  (Sung et al. 2012) | To investigate the effects of forest therapy on blood pressure, salivary cortisol and quality of life in patients with hypertension | Controlled trial | Enrolled for the study after referral from local health centres  (n = 56) | Blood pressure, salivary cortisol and quality of life | Primary measurement, bespoke questionnaire | Forest and control setting | Not specified | Quality of life ↑  Salivary cortisol ↓ |
| Takano 2002, Japan  (Takano et al. 2002) | To investigate the association between greenery filled public areas in close proximity to residences and the longevity of senior citizens in a densely populated, developed megacity | Cohort study | Representative sample of residents born in 1903, 1908, 1913 and 1918  (n = 3,144) | Longevity | Official 5 year survival rates | Self-reported neighbourhood greenspace and frequency of use; bespoke questionnaire | Controlled for age, sex, marital status and socioeconomic status | Longevity ↑  OR 1.13 (95% CI 1.03, 1.24) |
| Tamosiunas 2014, Lithuania  (Tamosiunas et al. 2014) | To explore the associations of the distance and use of urban greenspaces with the prevalence of cardiovascular diseases and its risk factors. To evaluate the impact of accessibility and use of greenspaces on the incidence of CVD | Cohort study | Kaunas cohort study. Mean age 60.4 years, 57% female  (n = 5,112) | Blood pressure, cognitive function, serum lipids, fasting glucose, self-reported health, symptoms of depression, coronary heart disease measured by a history of myocardial infarction or ischaemic changes in ECG. Angina, diabetes and stroke diagnosis recall | Primary measurement, 10-item Centre for Epidemiologic Studies Depression Scale (CES-D 10), medical records, bespoke questionnaire | Distance to nearest greenspace; unspecified special land cover dataset for Kaunas city | Adjusted for age | Total CVD ↓ *men only*  3^rd^ tertile compares to 1^st^:  HR 1.36 (95% CI 1.03, 1.80)  Non-fatal CVD ↓ *women only*  2^nd^ and 3^rd^ tertile compared to 1^st^:  HR 2.78 (95% CI 1.16, 6.70) |
| Toda 2013, Japan  (Toda et al. 2013) | To investigate the effect of walking through woodland on salivary endocrinological stress makers, cortisol and chromogranin A | Pre post study | Healthy males, mean age 67.6 years  (n = 20) | Salivary cortisol and chromogranin A, visual analogue scales of perceived stress | Primary measurement, self-reported stress visual analogue scale | Forest vs office | Matching of participants | Systolic blood pressure ↓  Salivary cortisol ↓  Heart rate ↑ |
| Triguero-Mas 2015, Spain  (Triguero-Mas et al. 2015) | To investigate the association between natural outdoor environments (separately blue and green spaces) and health (general and mental) and its possible mediators and modifiers | Cross-sectional study | Catalonia Health Survey. 50.06% female, mean age 48 years  (n = 8,793) | Self-perceived general health | Short form health survey (SF-36), General health questionnaire (GHQ-12), questions from ESCA questionnaire | Access to natural outdoor environments and surrounding greenness; Normalised Difference Vegetation Index (NDVI) | Adjusted for gender, age, education completed, birth place, type of health insurance, marital status and indicators of household and neighbourhood socioeconomic status | Self-reported health ↑  Surrounding greenness within 300m, OR for less than good self-perceived general health:  OR 0.90 (95% CI 0.83, 0.98) |
| Tsunetsugu 2007, Japan  (Tsunetsugu et al. 2007) | To investigate the physiological effects of shinrin-yoku on blood pressure, pulse rate, HRV, salivary cortisol and immunoglobin A | RCT | Male university students, mean age 22 years (range 21-23)  (n = 12) | Blood pressure, heart rate, heart rate variability (LF&HF), salivary cortisol and mental health | Primary measurement, bespoke 13-point scale | Forest vs city | Matching of participants | Blood pressure ↓  Heart rate ↓  Heart rate variability ↓  Salivary cortisol ↓ |
| Tsunetsugu 2013, Japan  (Tsunetsugu et al. 2013) | To investigate the physiological and psychological effects of viewing urban forest landscapes on 48 young male urban residents | Controlled trial | 12 university students who participated in each of the four experimental areas. Mean age 21.1 years  (n = 12) | Blood pressure, heart rate, heart rate variability | Primary measurement | Forest vs urban site | Matching of participants | Diastolic blood pressure ↓  Heart rate ↓  Heart rate variability ↓ |
| Tyrvainen 2014, Finland  (Tyrväinen et al. 2014) | To investigate the psychological and physiological effects of short-term visits to urban nature environments | RCT | Healthy non-smoking adults, mean age 47.64 years (range 30-61 years), 87.17% female  (n = 77) | Salivary cortisol concentration as well as psychological symptoms | Primary measurement, Focus of Attention Scale (TFOAS), Restoration Outcome Scale (ROS), Perceived Restorativeness Scale (PRS), Positive and Negative Affect Scale (PANAS), Creativity Scale, Subjective Vitality Scale | Forest vs urban park vs city centre | Matching of participants | Salivary cortisol ↔ |
| Ulmer 2016, USA  (Ulmer et al. 2016) | To enhance the understanding of the health-promoting potential of trees in an urbanised region of the US | Cross-sectional study | California Health Interview Survey (CHIS); 58% female, mean age 46 years  (n = 7,910) | Diabetes, blood pressure, asthma, general health status | California Health Interview Survey (CHIS) | LiDAR tree canopy cover data | All demographic, socio-economic and built environment variables were included in every model as covariates, and all models included adjustment for the DAC-provided raked sample weights | Poor general health ↓ OR 0.871 (0.799, 0.949)  Blood pressure, asthma, diabetes ↘ |
| Ulrich 1984, USA  (Ulrich 1984) | To investigate the influence of a hospital window view on patients’ emotional state and recovery | Retrospective cohort study | Cholecystectomy patients assigned to rooms on the second and third floors of a hospital between 1972 and 1981, during months when the trees had foliage. Age range 20-69. 65% female  (n = 46) | Number of days hospitalisation, number and strength of analgesics each day, number and strength of doses for anxiety, including tranquilisers and barbiturates, each day, minor complications such as persistent headache and nausea requiring medication-symptoms which are considered to result frequently from conversion reactions, and all nurses' notes relating to a patients condition or course of recovery | Hospital records on stay duration and medication type/frequency, nurses' notes | View from hospital window | Participants matched for age, sex, smoking status, obesity, history of hospitalisation, year of surgery and floor level | Post-operative recovery time ↓ |
| van Dillen 2012, The Netherlands  (van Dillen et al. 2012) | To investigate the link between the objectively assessed quantity and quality of (1) green areas and (2) streetscape greenery on the one hand and three self-reported health indicators on the other | Cross-sectional study | Questionnaires sent to a random sample of 100 households in each neighbourhood, non-western ethnic minorities heavily under-represented  (n = 1,641) | Health questionnaire: general health, acute health complaints, general mental health status | Bespoke questionnaire | Objectively measured quantity of green area; Pikora et al and Hillsdon et al tools | Adjusted for gender, age, education level and income | Self-reported health ↑ |
| Van Herzele 2012, Belgium  (Van Herzele and de Vries 2012) | To investigate the relationship between local greenness and health and wellbeing of inhabitants by looking at possible mediators :PA, stress, ability to concentrate, social cohesion and neighbourhood satisfaction | Cross-sectional study | Two neighbourhoods, Dierentuin (More green, n=97, 53.6% female, mean age 43.2 years) and Sint-Jacobs (Less green, n=93, 54.8% female, mean age 43.6 years)  (n = 190) | Self-reported general health, bodily functioning and general wellbeing | Bespoke questionnaire | Van Herzele and Wiedemann green space monitoring tool | Adjusted for gender, age, education, income, smoking, alcohol consumption and having a pessimistic personality | Self-reported health ↗ |
| Villeneuve 2012, Canada  (Villeneuve et al. 2012) | To investigate the relationship between urban greenspace and mortality in Ontario, Canada | Cohort study | Randomly selected from 10 urban areas in Ontario, >35 years, 50.8% male  (n = 574,840) | Canadian mortality database | Canadian mortality database, 2001 Canadian Community Health Survey | % neighbourhood greenspace; Normalised Difference Vegetation Index (NDVI) | Adjusted for smoking, physical activity and BMI category | Non-accidental mortality ↓  A 1IQR increase in greenspace in a 500m buffer associated with reduced non-accidental mortality:  RR 0.95 (95% CI 0.94, 0.96)  Respiratory disease mortality ↓  RR 0.91 (95% CI 0.89, 0.93) |
| Vogt 2015, Germany  (Vogt et al. 2015) | To examine the associations between proximity to two features of the residential environment and three aspects of health aging | Cross-sectional study | KORA (Cooperative Health Research in the Region of Augsburg)-Age study participants aged 65 or over  (n = 1,711) | Health-related quality of life | KORA-Age study survey | Distance to public green space, Augsburg city records | Model 2 controlled for age, sex and per capita income  Model 3 also controlled for regional deprivation | Health-related quality of life ↔ |
| Wang 2016, China  (Wang et al. 2016) | To examine the impact of both indoor and outdoor spatial factors on lung cancer | Hospital-based case-control study | 62% female  (n = 472) | Lung cancer morbidity | Bespoke questionnaire | Distance to parks, internal greenspace, dataset not specified | Participants matched for age and gender  Adjusted for other demographic and lifestyle factors | Lung cancer morbidity  ↓ with internal greenspace  ↑ with distance to parks |
| Ward 2016, New Zealand  (Ward et al. 2016) | To investigate the relationship between children’s time spent in greenspace with various physiological and psychological variables | Cross-sectional study | 59% female, mean age 12.66 years  (n = 108) | Cognitive development: visual memory, verbal memory, processing speed, psychomotor speed, reaction time, cognitive flexibility and executive function | Computerised neurocognitive testing conducted using CNS Vital Signs | Locational data from GPS, greenspace exposure calculated using Personal Activity Location Measurement System (PALMS) | All models included sex, age and school as covariates | Cognitive development ↔ |
| Ward Thompson 2012, Scotland  (Thompson et al. 2012) | To investigate the relationship between greenspace in urban deprived areas and stress (salivary cortisol and self-report) and general wellbeing | Exploratory study | People not-in-work were recruited through community centres and training opportunity centres in Dundee. Mean age 43.4 years (age range 33-57 years), 52% female  (n = 25) | Salivary cortisol, self-reported measures of stress and well-being | Primary measurement | Percentage neighbourhood greenspace in Census Area Statistics Ward; data from Centre for Research on Environment Society and Health (CRESH) | Not specified | Salivary cortisol ↓ |
| Ward Thompson 2016, Scotland  (Ward Thompson et al. 2016) | To investigate the nature of access to greenspace necessary before any health benefit is found | Cross-sectional survey | 54.7% female, mean age 44 ±17.1 years  (n = 406) | General health | Bespoke single-item assessment | Self-reported access to greenspace and objective measure using Ordnance Survey MasterMap | Not specified | General health ↑ |
| Weimann 2015, Sweden  (Weimann et al. 2015) | To investigate the effects of changing exposure to neighbourhood greenness on general and mental health | Longitudinal survey | Prognostic group for good general health at baseline: 48% male. Age range 18-80  (n = 8,891) | Self-reported general health | Bespoke questionnaire | Perceived neighbourhood greenness, public health survey | Adjusted for covariates associated with general or mental health | Self-reported health ↑  Evidence of beneficial effect of increased greenness indicated among subjects with lowest prognostic of good general health:  OR 1.24 (95% CI 1.01, 1.52) |
| Weltin 2012, USA  (Weltin and Lavin 2012) | To investigate whether a community garden could provide improved diabetes control | A mixed-convergent parallel designed intervention study | Members of a Midwest community of immigrants from the Marshall Islands. Mean age 51 years (range 33-81 years), 52.9% male  (n = 17) | HgA1c levels | Primary measurement | Community garden | Matching of participants | HbA1c ↓ |
| Wheeler 2012, UK  (Wheeler et al. 2012) | To investigate whether rates of good health improve with proximity to the coast and percentage green space | Cross-sectional ecological study | 2001 census data for England  (n = 48,200,000) | Self-reported "good" health | 2001 census data for England | % land area classified as greenspace in Lower-layer Super Output Areas (LSOA), Generalised Land Use Database | Not specified | Self-reported health ↑  Quintile 3: Rural: 0.31 (95% CI 0.04, 0.57)  Quintile 4: Urban: 0.23 (95% CI 0.13, 0.33)  Town/fringe: 0.49 (95% CI 0.19, 0.79)  Quintile 5: Rural 0.36 (95% CI 0.26, 0.47)  Town/fringe: 0.69 (95% CI 0.39, 0.99)  Rural: 0.59 (95% CI 0.30, 0.88) |
| Wheeler 2015, UK  (Wheeler et al. 2015) | To investigate the relationship between different types and qualities of natural environments on health and well-being | Ecological study | 2011 UK census data  (n = 63,260,000) | Age/sex standardised prevalence of both good and bad health | 2011 census data for Great Britain | Greenspace per Lower-layer Super Output Areas (LSOA) for England and Wales, and Data Zones (DZs) for Scotland; UK Land Cover Map 2007 | Regression analyses adjusted for income, education and employment scores and models also adjusted for urban/rural classification | Self-reported health ↑  Significant positive associations observed between good health prevalence and the density of several greenspace types: ‘broadleaf woodland’, ‘arable and horticulture’ and ‘improved grassland’ as well as ‘saltwater’ and ‘coastal’ after adjusting for confounders.  Broadleaf woodland: 0.32 (95% CI 0.029, 0.035)  Arable and horticulture: 0.004 (95% CI 0.002, 0.005)  Improved grassland: 0.016 (95% CI 0.014, 0.018) |
| Wilker 2014, USA  (Wilker et al. 2014) | To investigate the association between greenspace and post-stroke mortality | Hospital-based cohort study | Patients ≥21 years admitted to Beth Israel Deaconess Medical Centre (BIDMC) between 1999-2008 with acute ischaemic stroke. Mean age and gender by GS quartile: Q1: 73 years, 46% male; Q2: 75 years, 41% male; Q3: 76 years, 47% male; Q4: 77 years, 46% male  (n = 1,645) | History of acute ischaemic stroke | Hospital admission records; Beth Israel Deaconess Medical Centre (BIDMC) | Residential greenspace; Normalised Difference Vegetation Index (NDVI) | Model 1: adjusted for age, sex  Model 2: adjusted for age, sex, race. Hispanic, smoking status, history of coronary artery disease, history of stroke, atrial fibrillation, heart failure, diabetes, dyslipidaemia, hypertension, education and household income  Model 3: adjusted for model 2 covariates and the log of distance to a road with >10,000 cars/day | Mortality after ischaemic stroke ↓  Quartile 3: HR 0.79 (95% CI 0.65, 0.96)  Quartile 4: HR 0.80 (95% CI 0.65, 0.99)  (fully adjusted models) |
| Wolfe 2014, The Netherlands  (Wolfe et al. 2014) | To investigate changes in self-rated health of chronically ill people in relation to greenspace in their living environment at baseline | Prospective study | Health data from the national panel of people with chronic illness or disability (NPCD). ≥15 years and with a medically diagnosed somatic chronic disease on average 9.7 years prior to inclusion  (n = 1,112) | Self-rated health | Bespoke questionnaire including 5-item General Health Perception Scale of the RAND-36 | Perceived neighbourhood greenness and urbanity; National Land Cover Classification Database 2003/2004 and Statistics Netherlands 2004 | Controlled for ‘other correlates of health’ | Self-reported health ↗ |
| Wu 2015, England  (Wu et al. 2015) | To investigate the impact of the community environment on cognition in later life | Cross-sectional study | The MRC Cognitive Function and Ageing Study (CFAS). 60.7% female, mean age 81.7 years  (n = 2,424) | Cognitive impairment and dementia | Bespoke questionnaire including self-reported past medical history | Land use per Lower-layer Super Output Areas (LSOA); Generalised Land Use Dataset (GLUD) 2001 | Adjusted for age, gender, education, social class and number of chronic illnesses with a further adjustment for area deprivation | Dementia ↑  Quartile 4 (Highest % natural environment) OR 2.23 (95% CI 1.17, 4.24)  Cognitive impairment ↑  Quartile 4 OR (95% CI 1.00, 1.98) |
| Yamaguchi 2006, Japan  (Yamaguchi et al. 2006) | To investigate the effects of exercise in forest and urban environments on sympathetic nervous activity of normal young adults | Intervention study | Healthy male university students, mean age 22.2 years  (n = 15) | Salivary amylase activity, sympathetic nervous activity (heart rate variability) | Primary measurement | Forest vs urban environment | Matching of participants | Heart rate variability ↓ |
| Young 2016, USA  (Young et al. 2016) | To determine the risk of gestational diabetes (GDM) and preeclampsia associated with various community response | Ecological study | Los Angeles and Orange Counties birth records  (n = 6,567,580 women, 362,525 pregnancies) | Gestational diabetes and preeclampsia | California Birth Certificate database | Ratio and km of park area in each zipcode; land use data from Southern California Association of Government | Adjusted model accounted for maternal age, prepregnancy BMI, race, ethnicity and median household income | Gestational diabetes and preeclampsia ↔ |

1. ↑= significant increase; ↓= significant decrease; ↗= non-significant increase; ↘= non-significant decrease; ↔= non-significant, direction not reported.
2. Significant increase in a measurement may or may not be an improvement

References:

Agay-Shay K, Peled A, Crespo AV, Peretz C, Amitai Y, Linn S, et al. 2014. Green spaces and adverse pregnancy outcomes. J Occup Env Med 71:562-569.

Agyemang C, Van Hooijdonk C, Wendel-Vos W, Ujcic-Voortman JK, Lindeman E, Stronks K, et al. 2007. Ethnic differences in the effect of environmental stressors on blood pressure and hypertension in the netherlands. BMC Public Health 7:118.

Andrusaityte S, Grazuleviciene R, Kudzyte J, Bernotiene A, Dedele A, Nieuwenhuijsen MJ. 2016. Associations between neighbourhood greenness and asthma in preschool children in kaunas, lithuania: A case–control study. BMJ Open 6:e010341.

Arbillaga-Etxarri A, Torrent-Pallicer J, Gimeno-Santos E, Barberan-Garcia A, Delgado A, Balcells E, et al. 2016. Validation of walking trails for the urban training tm of chronic obstructive pulmonary disease patients. PloS One 11:e0146705.

Astell-Burt T, Feng X, Kolt GS. 2013. Does access to neighbourhood green space promote a healthy duration of sleep? Novel findings from a cross-sectional study of 259 319 australians. BMJ Open 3.

Astell-Burt T, Feng X, Kolt GS. 2014a. Neighbourhood green space and the odds of having skin cancer: Multilevel evidence of survey data from 267 072 australians. J Epidemiol Community Health 68:370-374.

Astell-Burt T, Feng X, Kolt GS. 2014b. Is neighborhood green space associated with a lower risk of type 2 diabetes evidence from 267,072 australians. Diabetes Care 37:197-201.

Beil K, Hanes D. 2013. The influence of urban natural and built environments on physiological and psychological measures of stress- a pilot study. Int J Environ Res Public Health 10:1250-1267.

Besenyi GM, Kaczynski AT, Stanis SAW, Bergstrom RD, Lightner JS, Hipp JA. 2014. Planning for health: A community-based spatial analysis of park availability and chronic disease across the lifespan. Health Place 27:102-105.

Bijnens E, Zeegers MP, Gielen M, Kicinski M, Hageman GJ, Pachen D, et al. 2015. Lower placental telomere length may be attributed to maternal residential traffic exposure; a twin study. Environ Int 79:1-7.

Bixby H, Hodgson S, Fortunato L, Hansell A, Fecht D. 2015. Associations between green space and health in english cities: An ecological, cross-sectional study. PloS One 10:e0119495.

Bodicoat DH, O'Donovan G, Dalton AM, Gray LJ, Yates T, Edwardson C, et al. 2014. The association between neighbourhood greenspace and type 2 diabetes in a large cross-sectional study. BMJ Open 4:e006076.

Botticello AL, Rohrbach T, Cobbold N. 2015. Differences in the community built environment influence poor perceived health among persons with spinal cord injury. Arch Phys Med Rehabil 96:1583-1590.

Brown SC, Lombard J, Wang K, Byrne MM, Toro M, Plater-Zyberk E, et al. 2016. Neighborhood greenness and chronic health conditions in medicare beneficiaries. Am J Prev Med 51:78-89.

Burkart K, Meier F, Schneider A, Breitner S, Canario P, Alcoforado MJ, et al. 2016. Modification of heat-related mortality in an elderly urban population by vegetation (urban green) and proximity to water (urban blue): Evidence from lisbon, portugal. Environ Health Perspect 124:927-934.

Calogiuri G, Evensen K, Weydahl A, Andersson K, Patil G, Ihlebæk C, et al. 2016. Green exercise as a workplace intervention to reduce job stress. Results from a pilot study. Work 53:99-111.

Casey JA, James P, Rudolph KE, Wu CD, Schwartz BS. 2016. Greenness and birth outcomes in a range of pennsylvania communities. Int J Environ Res Public Health 13 e311.

Chum A, O’Campo P. 2015. Cross-sectional associations between residential environmental exposures and cardiovascular diseases. BMC Public Health 15:438.

Coutts C, Horner M, Chapin T. 2010. Using geographical information system to model the effects of green space accessibility on mortality in florida. Geocarto Int 25:471-484.

Coutts CJ, Horner MW. 2015. Nature and death: An individual level analysis of the relationship between biophilic environments and premature mortality in florida. Spatial Analysis in Health Geography 295.

Cusack L, Larkin A, Carozza S, Hystad P. 2017. Associations between residential greenness and birth outcomes across texas. Environ Res 152:88-95.

Dadvand P, Sunyer J, BasagaÃ±a X, Ballester F, Lertxundi A, FernÃ¡ndez-Somoano A, et al. 2012a. Surrounding greenness and pregnancy outcomes in four spanish birth cohorts. Environ Health Perspect 120:1481-1487.

Dadvand P, de Nazelle A, Figueras F, Basagana X, Su J, Amoly E, et al. 2012b. Green space, health inequality and pregnancy. Environ Int 40:110-115.

Dadvand P, Villanueva CM, Font-Ribera L, Martinez D, Basagaña X, Belmonte J, et al. 2014. Risks and benefits of green spaces for children: A cross-sectional study of associations with sedentary behavior, obesity, asthma, and allergy. Environ Health Perspect 122:1329-1335.

Dadvand P, Nieuwenhuijsen MJ, Esnaola M, Forns J, Basagaña X, Alvarez-Pedrerol M, et al. 2015. Green spaces and cognitive development in primary schoolchildren. Proc Natl Acad Sci USA 112:7937-7942.

Dadvand P, Bartoll X, Basagaña X, Dalmau-Bueno A, Martinez D, Ambros A, et al. 2016. Green spaces and general health: Roles of mental health status, social support, and physical activity. Environ Int 91:161-167.

Dalton AM, Jones AP, Sharp SJ, Cooper AJ, Griffin S, Wareham NJ. 2016. Residential neighbourhood greenspace is associated with reduced risk of incident diabetes in older people: A prospective cohort study. BMC Public Health 16:1171.

de Jong K, Albin M, Skarback E, Grahn P, Bjork J. 2012. Perceived green qualities were associated with neighborhood satisfaction, physical activity, and general health: Results from a cross-sectional study in suburban and rural scania, southern sweden. Health Place 18:1374-1380.

De Vries S, Verheij RA, Groenewegen PP, Spreeuwenberg P. 2003. Natural environments—healthy environments? An exploratory analysis of the relationship between greenspace and health. Environ Plan A 35:1717-1731.

Demoury C, Thierry B, Richard H, Sigler B, Kestens Y, Parent M-E. 2017. Residential greenness and risk of prostate cancer: A case-control study in montreal, canada. Environ Int 98:129-136.

Donovan GH, Michael YL, Butry DT, Sullivan AD, Chase JM. 2011. Urban trees and the risk of poor birth outcomes. Health Place 17:390-393.

Droomers M, Jongeneel-Grimen B, Kramer D, de Vries S, Kremers S, Bruggink JW, et al. 2016. The impact of intervening in green space in dutch deprived neighbourhoods on physical activity and general health: Results from the quasi-experimental urban40 study. J Epidemiol Community Health 70:147-154.

Dunstan F, Fone DL, Glickman M, Palmer S. 2013. Objectively measured residential environment and self-reported health: A multilevel analysis of uk census data. PloS One 8:e69045.

Fjørtoft I. 2004. Landscape as playscape: The effects of natural environments on children's play and motor development. Child Youth Environ 14:21-44.

Fuertes E, Markevych I, von Berg A, Bauer C-P, Berdel D, Koletzko S, et al. 2014. Greenness and allergies: Evidence of differential associations in two areas in germany. J Epidemiol Community Health 68:787-790.

Gong Y, Gallacher J, Palmer S, Fone D. 2014. Neighbourhood green space, physical function and participation in physical activities among elderly men: The caerphilly prospective study. Int J Behav Nutr Phys Act 11:40.

Grazuleviciene R, Dedele A, Danileviciute A, Vencloviene J, Grazulevicius T, Andrusaityte S, et al. 2014a. The influence of proximity to city parks on blood pressure in early pregnancy. Int J Environ Res Public Health 11:2958-2972.

Grazuleviciene R, Danileviciute A, Dedele A, Vencloviene J, Andrusaityte S, Uždanaviciute I, et al. 2015a. Surrounding greenness, proximity to city parks and pregnancy outcomes in kaunas cohort study. Int J Hyg Environ Health 218:358-365.

Grazuleviciene R, Vencloviene J, Kubilius R, Grizas V, Dedele A, Grazulevicius T, et al. 2015b. The effect of park and urban environments on coronary artery disease patients: A randomized trial. Biomed Res Int 2015.

Grazuleviciene R, Vencloviene J, Kubilius R, Grizas V, Danileviciute A, Dedele A, et al. 2016. Tracking restoration of park and urban street settings in coronary artery disease patients. Int J Environ Res Public Health 13:e550.

Grigsby-Toussaint DS, Turi KN, Krupa M, Williams NJ, Pandi-Perumal SR, Jean-Louis G. 2015. Sleep insufficiency and the natural environment: Results from the us behavioral risk factor surveillance system survey. Prev Med 78:78-84.

Gutiérrez-Zornoza M, Sánchez-López M, García-Hermoso A, González-García A, Chillón P, Martínez-Vizcaíno V. 2014. Active commuting to school, weight status, and cardiometabolic risk in children from rural areas: The cuenca study. Health Educ Behav 42:231-239.

Hartig T, Evans GW, Jamner LD, Davis DS, Gärling T. 2003. Tracking restoration in natural and urban field settings. Journal of environmental psychology 23:109-123.

Hoehner CM, Allen P, Barlow CE, Marx CM, Brownson RC, Schootman M. 2013. Understanding the independent and joint associations of the home and workplace built environments on cardiorespiratory fitness and body mass index. Am J Epidemiol 178:1094-1105.

Hu Z, Liebens J, Rao KR. 2008. Linking stroke mortality with air pollution, income, and greenness in northwest florida: An ecological geographical study. Int J Health Geogr 7:20.

Hystad P, Davies HW, Frank L, Loon JV, Gehring U, Tamburic L, et al. 2014. Residential greenness and birth outcomes: Evaluating the influence of spatially correlated built-environment factors. Environ Health Perspect 122:1095-1102.

James P, Hart JE, Banay RF, Laden F. 2016. Exposure to greenness and mortality in a nationwide prospective cohort study of women. Environ Health Perspect 124:1344-1352.

Jia BB, Yang ZX, Mao GX, Lyu YD, Wen XL, Xu WH, et al. 2016. Health effect of forest bathing trip on elderly patients with chronic obstructive pulmonary disease. Biomed Environ Sci 29:212-218.

Jonker MF, van Lenthe FJ, Donkers B, Mackenbach JP, Burdorf A. 2014. The effect of urban green on small-area (healthy) life expectancy. J Epidemiol Community Health 68:999-1002.

Kabisch N, Haase D, Annerstedt van den Bosch M. 2016. Adding natural areas to social indicators of intra-urban health inequalities among children: A case study from berlin, germany. Int J Environ Res Public Health 13:783.

Kardan O, Gozdyra P, Misic B, Moola F, Palmer LJ, Paus T, et al. 2015. Neighborhood greenspace and health in a large urban center. Sci Rep 5:11610.

Kihal-Talantikite W, Padilla CM, Lalloue B, Gelormini M, Zmirou-Navier D, Deguen S. 2013. Green space, social inequalities and neonatal mortality in france. BMC Pregnancy Childbirth 13:191.

Kim BJ, Jeong H, Park S, Lee S. 2015. Forest adjuvant anti-cancer therapy to enhance natural cytotoxicity in urban women with breast cancer: A preliminary prospective interventional study. Eur J Integr Med 7:474-478.

Kim H-J, Min J-Y, Kim H-J, Min K-B. 2016. Parks and green areas are associated with decreased risk for hyperlipidemia. Int J Environ Res Public Health 13:1205.

Lachowycz K, Jones AP. 2014. Does walking explain associations between access to greenspace and lower mortality? Soc Sci Med 107:9-17.

Larson LR, Jennings V, Cloutier SA. 2016. Public parks and wellbeing in urban areas of the united states. PLoS One 11:e0153211.

Laurent O, Wu J, Li L, Milesi C. 2013. Green spaces and pregnancy outcomes in southern california. Health Place 24:190-195.

Lee J, Park BJ, Tsunetsugu Y, Ohira T, Kagawa T, Miyazaki Y. 2011. Effect of forest bathing on physiological and psychological responses in young japanese male subjects. Public Health 125:93-100.

Lee J, Tsunetsugu Y, Takayama N, Park B-J, Li Q, Song C, et al. 2014a. Influence of forest therapy on cardiovascular relaxation in young adults. Evid Based Complement Alternat Med 2014.

Lee J-Y, Lee D-C. 2014b. Cardiac and pulmonary benefits of forest walking versus city walking in elderly women: A randomised, controlled, open-label trial. Eur J Integr Med 6:5-11.

Li Q, Morimoto K, Kobayashi M, Inagaki H, Katsumata M, Hirata Y, et al. 2008a. A forest bathing trip increases human natural killer activity and expression of anti-cancer proteins in female subjects. J Biol Regul Homeost Agents 22:45-55.

Li Q, Morimoto K, Kobayashi M, Inagaki H, Katsumata M, Hirata Y, et al. 2008b. Visiting a forest, but not a city, increases human natural killer activity and expression of anti-cancer proteins. Int J Immunopathol Pharmacol 21:117-127.

Li Q, Kobayashi M, Inagaki H, Hirata Y, Li Y, Hirata K, et al. 2009. A day trip to a forest park increases human natural killer activity and the expression of anti-cancer proteins in male subjects. J Biol Regul Homeost Agents 24:157-165.

Li Q, Otsuka T, Kobayashi M, Wakayama Y, Inagaki H, Katsumata M, et al. 2011. Acute effects of walking in forest environments on cardiovascular and metabolic parameters. Eur J Appl Physiol 111:2845-2853.

Li Q, Kobayashi M, Kumeda S, Ochiai T, Miura T, Kagawa T, et al. 2016. Effects of forest bathing on cardiovascular and metabolic parameters in middle-aged males. Evid Based Complement Alternat Med 2016.

Lovasi GS, Quinn JW, Neckerman KM, Perzanowski MS, Rundle A. 2008. Children living in areas with more street trees have lower prevalence of asthma. J Epidemiol Community Health 62:647-649.

Lovasi GS, O'Neil-Dunne JPM, Lu JWT, Sheehan D, Perzanowski MS, Macfaden SW, et al. 2013. Urban tree canopy and asthma, wheeze, rhinitis, and allergic sensitization to tree pollen in a new york city birth cohort. Environ Health Perspect 121:494-500.

Maas J, Verheij RA, Groenewegen PP, de Vries S, Spreeuwenberg P. 2006. Green space, urbanity, and health: How strong is the relation? J Epidemiol Community Health 60:587-592.

Maas J, Verheij RA, Spreeuwenberg P, Groenewegen PP. 2008. Physical activity as a possible mechanism behind the relationship between green space and health: A multilevel analysis. BMC Public Health 8:206-206.

Maas J, van Dillen SM, Verheij RA, Groenewegen PP. 2009a. Social contacts as a possible mechanism behind the relation between green space and health. Health Place 15:586-595.

Maas J, Verheij RA, de Vries S, Spreeuwenberg P, Schellevis FG, Groenewege PP. 2009b. Morbidity is related to a green living environment. J Epidemiol Community Health 63:967-973.

Mao G, Cao Y, Lan X, He Z, Chen Z, Wang Y, et al. 2012a. Therapeutic effect of forest bathing on human hypertension in the elderly. J Cardiol 60:495-502.

Mao G, Lan X, Cao Y, Chen Z, He Z, Lv Y, et al. 2012b. Effects of short-term forest bathing on human health in a broad-leaved evergreen forest in zhejiang province, china. Biomed Environ Sci 25:317-324.

Markevych I, Thiering E, Fuertes E, Sugiri D, Berdel D, Koletzko S, et al. 2014. A cross-sectional analysis of the effects of residential greenness on blood pressure in 10-year old children: Results from the giniplus and lisaplus studies. BMC Public Health 14:477.

Markevych I, Standl M, Sugiri D, Harris C, Maier W, Berdel D, et al. 2016. Residential greenness and blood lipids in children: A longitudinal analysis in giniplus and lisaplus. Environ Res 151:168-173.

Matsunaga K, Park BJ, Kobayashi H, Miyazaki Y. 2011. Physiologically relaxing effect of a hospital rooftop forest on older women requiring care. J Am Geriatr Soc 59:2162-2163.

McCracken DS, Allen DA, Gow AJ. 2016. Associations between urban greenspace and health-related quality of life in children. Prev Med Rep 3:211-221.

Mitchell R, Popham F. 2007. Greenspace, urbanity and health: Relationships in england. J Epidemiol Community Health 61:681-683.

Mitchell R, Popham F. 2008. Effect of exposure to natural environment on health inequalities: An observational population study. Lancet 372:1655-1660.

Mitchell R, Astell-Burt T, Richardson EA. 2011. A comparison of green space indicators for epidemiological research. J Epidemiol Community Health 65:853-858.

Morita E, Naito M, Hishida A, Wakai K, Mori A, Asai Y, et al. 2011. No association between the frequency of forest walking and blood pressure levels or the prevalence of hypertension in a cross-sectional study of a japanese population. Environ Health Prev Med 16:299-306.

Nakau M, Imanishi J, Imanishi J, Watanabe S, Imanishi A, Baba T, et al. 2013. Spiritual care of cancer patients by integrated medicine in urban green space: A pilot study. Explore (NY) 9:87-90.

Ngom R, Gosselin P, Blais C, Rochette L. 2016. Type and proximity of green spaces are important for preventing cardiovascular morbidity and diabetes-a cross-sectional study for quebec, canada. Int J Environ Res Public Health 13:423.

Ochiai H, Ikei H, Song C, Kobayashi M, Takamatsu A, Miura T, et al. 2015. Physiological and psychological effects of forest therapy on middle-aged males with high-normal blood pressure. Int J Environ Res Public Health 12:2532-2542.

Ohtsuka Y, Yabunaka N, Takayama S. 1998. Shinrin-yoku (forest-air bathing and walking) effectively decreases blood glucose levels in diabetic patients. Int J Biometeorol 41:125-127.

Padilla CM, Kihal-Talantikit W, Perez S, Deguen S. 2016. Use of geographic indicators of healthcare, environment and socioeconomic factors to characterize environmental health disparities. Environ Health 15:79.

Paquet C, Coffee NT, Haren MT, Howard NJ, Adams RJ, Taylor AW, et al. 2014. Food environment, walkability, and public open spaces are associated with incident development of cardio-metabolic risk factors in a biomedical cohort. Health Place 28:173-176.

Park B-J, Tsunetsugu Y, Kasetani T, Hirano H, Kagawa T, Sato M, et al. 2007. Physiological effects of shinrin-yoku (taking in the atmosphere of the forest)-using salivary cortisol and cerebral activity as indicators. J Physiol Anthropol 26:123-128.

Park B-J, Tsunetsugu Y, Kasetani T, Morikawa T, Kagawa T, Miyazaki Y. 2009. Physiological effects of forest recreation in a young conifer forest in hinokage town, japan. Silva Fenn 43:291-301.

Park BJ, Tsunetsugu Y, Kasetani T, Kagawa T, Miyazaki Y. 2010. The physiological effects of shinrin-yoku (taking in the forest atmosphere or forest bathing): Evidence from field experiments in 24 forests across japan. Environ Health Prev Med 15:18-26.

Pasanen TP, Tyrväinen L, Korpela KM. 2014. The relationship between perceived health and physical activity indoors, outdoors in built environments, and outdoors in nature. Appl Psychol Health Well Being 6:324-346.

Pereira G, Foster S, Martin K, Christian H, Boruff BJ, Knuiman M, et al. 2012. The association between neighborhood greenness and cardiovascular disease: An observational study. BMC Public Health 12:466-466.

Picavet HSJ, Milder I, Kruize H, de Vries S, Hermans T, Wendel-Vos W. 2016. Greener living environment healthier people? Exploring green space, physical activity and health in the doetinchem cohort study. Prev Med 89:7-14.

Piccolo RS, Duncan DT, Pearce N, McKinlay JB. 2015. The role of neighborhood characteristics in racial/ethnic disparities in type 2 diabetes: Results from the boston area community health (bach) survey. Soc Sci Med 130:79-90.

Pietilä M, Neuvonen M, Borodulin K, Korpela K, Sievänen T, Tyrväinen L. 2015. Relationships between exposure to urban green spaces, physical activity and self-rated health. JORT 10:44-54.

Putrik P, de Vries N, Mujakovic S, van Amelsvoort L, Kant I, Kunst A, et al. 2015. Living environment matters: Relationships between neighborhood characteristics and health of the residents in a dutch municipality. J Community Health 40:47-56.

Qin J, Zhou X, Sun C, Leng H, Lian Z. 2013. Influence of green spaces on environmental satisfaction and physiological status of urban residents. Urban For Urban Gree 12:490-497.

Reklaitiene R, Grazuleviciene R, Dedele A, Virviciute D, Vensloviene J, Tamosiunas A, et al. 2014. The relationship of green space, depressive symptoms and perceived general health in urban population. Scand J Public Health 42:669-676.

Requia WJ, Roig HL, Adams MD, Zanobetti A, Koutrakis P. 2016. Mapping distance-decay of cardiorespiratory disease risk related to neighborhood environments. Environ Res 151:203-215.

Richardson E, Pearce J, Mitchell R, Day P, Kingham S. 2010a. The association between green space and cause-specific mortality in urban new zealand: An ecological analysis of green space utility. BMC Public Health 10:240.

Richardson EA, Mitchell R. 2010b. Gender differences in relationships between urban green space and health in the united kingdom. Soc Sci Med 71:568-575.

Richardson EA, Mitchell R, Hartig T, de Vries S, Astell-Burt T, Frumkin H. 2012. Green cities and health: A question of scale? J Epidemiol Community Health 66:160-165.

Richardson EA, Pearce J, Mitchell R, Kingham S. 2013. Role of physical activity in the relationship between urban green space and health. Public Health 127:318-324.

Roe J, Aspinall PA, Thompson CW. 2016. Understanding relationships between health, ethnicity, place and the role of urban green space in deprived urban communities. Int J Environ Res Public Health 13:e681.

Roe JJ, Ward Thompson C, Aspinall PA, Brewer MJ, Duff EI, Miller D, et al. 2013. Green space and stress: Evidence from cortisol measures in deprived urban communities. Int J Environ Res Public Health 10:4086-4103.

Ruokolainen L, Von Hertzen L, Fyhrquist N, Laatikainen T, Lehtomaki J, Auvinen P, et al. 2015. Green areas around homes reduce atopic sensitization in children. Allergy 70:195-202.

Sbihi H, Tamburic L, Koehoorn M, Brauer M. 2015. Greenness and incident childhood asthma: A 10-year follow-up in a population-based birth cohort. Am J Respir Crit Care Med 192:1131-1133.

Skarková P, Kadlubiec R, Fischer M, Kratenová J, Zapletal M, Vrubel J. 2015. Refining of asthma prevalence spatial distribution and visualization of outdoor environment factors using gis and its application for identification of mutual associations. Cent Eur J Public Health 23:258.

Song C, Joung D, Ikei H, Igarashi M, Aga M, Park BJ, et al. 2013. Physiological and psychological effects of walking on young males in urban parks in winter. J Physiol Anthropol 32:18.

Song C, Ikei H, Kobayashi M, Miura T, Taue M, Kagawa T, et al. 2015a. Effect of forest walking on autonomic nervous system activity in middle-aged hypertensive individuals: A pilot study. Int J Environ Res Public Health 12:2687-2699.

Song C, Ikei H, Igarashi M, Takagaki M, Miyazaki Y. 2015b. Physiological and psychological effects of a walk in urban parks in fall. Int J Environ Res Public Health 12:14216-14228.

Stigsdotter UK, Ekholm O, Schipperijn J, Toftager M, Kamper-Jørgensen F, Randrup TB. 2010. Health promoting outdoor environments -- associations between green space, and health, health-related quality of life and stress based on a danish national representative survey. Scand J Public Health 38:411-417.

Sugaya S, Kasetani T, Zhong Q-J, Wen-Zhi G. 2011. Studies on the amounts of serum hydroperoxide, mmp-3, urinary 8-ohdg, and salivary iga in rheumatoid arthritis patients who experienced shinrin-yoku (forest-air bathing and walking). J Chiba Med Soc 87:181-188.

Sugiyama T, Leslie E, Giles-Corti B, Owen N. 2008. Associations of neighbourhood greenness with physical and mental health: Do walking, social coherence and local social interaction explain the relationships? J Epidemiol Community Health 62:e9-e9.

Sugiyama T, Thompson CW, Alves S. 2009. Associations between neighborhood open space attributes and quality of life for older people in britain. Environ Behav 41:3-21.

Sulander T, Karvinen E, Holopainen M. 2016. Urban green space visits and mortality among older adults. Epidemiology 27:e34-e35.

Sung J, Woo J-M, Kim W, Lim S-K, Chung E-J. 2012. The effect of cognitive behavior therapy-based “forest therapy” program on blood pressure, salivary cortisol level, and quality of life in elderly hypertensive patients. Clin Exp Hypertens 34:1-7.

Takano T, Nakamura K, Watanabe M. 2002. Urban residential environments and senior citizens' longevity in megacity areas: The importance of walkable green spaces. J Epidemiol Community Health 56:913-918.

Tamosiunas A, Grazuleviciene R, Luksiene D, Dedele A, Reklaitiene R, Baceviciene M, et al. 2014. Accessibility and use of urban green spaces, and cardiovascular health: Findings from a kaunas cohort study. Environ Health 13:20.

Thompson CW, Roe J, Aspinall P, Mitchell R, Clow A, Miller D. 2012. More green space is linked to less stress in deprived communities: Evidence from salivary cortisol patterns. Landsc Urban Plan 105:221-229.

Toda M, Den R, Hasegawa-Ohira M, Morimoto K. 2013. Effects of woodland walking on salivary stress markers cortisol and chromogranin a. Complement Ther Med 21:29-34.

Triguero-Mas M, Dadvand P, Cirach M, Martinez D, Medina A, Mompart A, et al. 2015. Natural outdoor environments and mental and physical health: Relationships and mechanisms. Environ Int 77:35-41.

Tsunetsugu Y, Park B-J, Ishii H, Hirano H, Kagawa T, Miyazaki Y. 2007. Physiological effects of shinrin-yoku (taking in the atmosphere of the forest) in an old-growth broadleaf forest in yamagata prefecture, japan. J Physiol Anthropol 26:135-142.

Tsunetsugu Y, Lee J, Park B-J, Tyrväinen L, Kagawa T, Miyazaki Y. 2013. Physiological and psychological effects of viewing urban forest landscapes assessed by multiple measurements. Landscape Urban Plan 113:90-93.

Tyrväinen L, Ojala A, Korpela K, Lanki T, Tsunetsugu Y, Kagawa T. 2014. The influence of urban green environments on stress relief measures: A field experiment. J Environ Psychol 38:1-9.

Ulmer JM, Wolf KL, Backman DR, Tretheway RL, Blain CJ, O’Neil-Dunne JP, et al. 2016. Multiple health benefits of urban tree canopy: The mounting evidence for a green prescription. Health Place 42:54-62.

Ulrich R. 1984. View through a window may influence recovery. Science 224:224-225.

van Dillen SM, de Vries S, Groenewegen PP, Spreeuwenberg P. 2012. Greenspace in urban neighbourhoods and residents' health: Adding quality to quantity. J Epidemiol Community Health 66:e8.

Van Herzele A, de Vries S. 2012. Linking green space to health: A comparative study of two urban neighbourhoods in ghent, belgium. Popul Environ 34:171-193.

Villeneuve PJ, Jerrett M, J GS, Burnett RT, Chen H, Wheeler AJ, et al. 2012. A cohort study relating urban green space with mortality in ontario, canada. Environ Res 115:51-58.

Vogt S, Mielck A, Berger U, Grill E, Peters A, Döring A, et al. 2015. Neighborhood and healthy aging in a german city: Distances to green space and senior service centers and their associations with physical constitution, disability, and health-related quality of life. Eur J Ageing 12:273-283.

Wang L, Zhao X, Xu W, Tang J, Jiang X. 2016. Correlation analysis of lung cancer and urban spatial factor: Based on survey in shanghai. J Thorac Dis 8:2626-2637.

Ward JS, Duncan JS, Jarden A, Stewart T. 2016. The impact of children's exposure to greenspace on physical activity, cognitive development, emotional wellbeing, and ability to appraise risk. Health Place 40:44-50.

Ward Thompson C, Aspinall P, Roe J, Robertson L, Miller D. 2016. Mitigating stress and supporting health in deprived urban communities: The importance of green space and the social environment. Int J Environ Res Public Health 13:440.

Weimann H, Rylander L, Albin M, Skärbäck E, Grahn P, Östergren P-O, et al. 2015. Effects of changing exposure to neighbourhood greenness on general and mental health: A longitudinal study. Health Place 33:48-56.

Weltin AM, Lavin RP. 2012. The effect of a community garden on hga1c in diabetics of marshallese descent. Journal of Community Health Nursing 29:12-24.

Wheeler BW, White M, Stahl-Timmins W, Depledge MH. 2012. Does living by the coast improve health and wellbeing? Health Place 18:1198-1201.

Wheeler BW, Lovell R, Higgins SL, White MP, Alcock I, Osborne NJ, et al. 2015. Beyond greenspace: An ecological study of population general health and indicators of natural environment type and quality. Int J Health Geogr 14:1.

Wilker E, Wu CD, McNeely E, Mostofsky E, Spengler J, Wellenius G, et al. 2014. Green space and mortality following ischemic stroke. Environ Res 129:42-48.

Wolfe MK, Groenewegen PP, Rijken M, de Vries S. 2014. Green space and changes in self-rated health among people with chronic illness. Eur J Public Health 24:640-642.

Wu Y-T, Prina AM, Jones AP, Barnes LE, Matthews FE, Brayne C. 2015. Community environment, cognitive impairment and dementia in later life: Results from the cognitive function and ageing study. Age Ageing 44:1005-1011.

Yamaguchi M, Deguchi M, Miyazaki Y. 2006. The effects of exercise in forest and urban environments on sympathetic nervous activity of normal young adults. J Int Med Res 34:152-159.

Young C, Laurent O, Chung JH, Wu J. 2016. Geographic distribution of healthy resources and adverse pregnancy outcomes. Matern Child Health J 20:1673-1679.
