# Supplementary material for: The health benefits of the great outdoors: A systematic review and meta-analysis of greenspace exposure and health outcomes
Source: Environ Res. 2018 Oct;166:628–37. doi: 10.1016/j.envres.2018.06.030 (PMC6562165; doi:10.1016/j.envres.2018.06.030)
Supplement: Supplementary file 2 — Supplementary material [file mmc2.docx]

**Appendix B**

**Supplementary table S2:** Search terms for electronic databases

| **Health outcome search terms** | disease* OR lower respiratory infection* OR upper respiratory infection* OR otitis media OR food-borne trematodiases OR maternal complication* OR pregnancy complica* OR hypertensive disorder* of pregnan* OR obstructed labour OR abortion OR maternal problem* OR birth complication* OR neonatal encephalopathy OR birth asphyxia OR birth trauma OR birth sepsis OR disorder* of the newborn baby OR neonatal disorder* OR hepatitis OR cancer* OR melanoma OR non-Hodgkin lymphoma OR leuk*mia OR neoplasm* OR cardiomyopathy OR myocarditis OR atrial fibrillation OR atrial flutter* OR aortic aneurysm OR endocarditis OR cardiovascular (Expanded to include: hypertension OR blood pressure OR dyslipidaemia OR hyperlipidaemia) COPD OR pneumoconiosis OR asthma OR pulmonary sarcoidosis OR cirrhosis OR peptic ulcer* OR gastritis OR duodenitis OR appendicitis OR paralytic ileus OR intestinal obstruction* OR hernia* OR vascular disorder* OR pancreatitis OR Alzheimer's OR dementia OR Parkinson's OR epilepsy OR multiple sclerosis OR migraine* OR tension type headache* OR neurological disorder* OR schizophrenia OR development disorders* OR behavioural disorder* OR intellectual disability* OR behavioural disorder* OR glomerulonephritis OR urinary OR infertility OR h*moglobinopath* OR haemolytic an*mia* OR endocrine disorder* OR blood disorder* OR immune disorder* OR rheumatoid arthritis OR osteoarthritis OR low* back pain OR neck pain OR gout OR musculoskeletal disorder* OR congenital anomal* OR neural tube defect* OR congenital heart OR oral disorder* OR sudden infant death OR road injury OR transport injury OR drowning OR poisoning* OR exposure to mechanical forces OR adverse effect* of medical treatment OR animal contact OR unintentional injur* OR Self?harm OR interpersonal violence OR health outcome* OR health stat* OR mortalit* OR morbidit* OR chronic disease* (Expanded to include: OR red blood cell count OR white blood cell count OR serum enzyme level OR serum antibody level OR plasma protein level OR hormone level OR autoimmune) OR life expectanc* OR work* stress OR work related stress OR hypertension OR stroke* OR disability?adjusted life year* OR quality?adjusted life year* OR daly* OR qaly* OR industrial *cident* OR industrial injur* OR birth weight OR physiological effects OR motor development OR heart rate variability OR blood pressure OR physical function OR cognitive function OR thyroid OR nutritional deficiency OR metabolic disorder OR inflammat* OR degenerative disease OR ischaemic heart disease OR pulmonary disease OR digestive system disorder OR bone density OR diabet* (Expanded to include: OR blood glucose OR HbA1c OR salivary cortisol) |
| --- | --- |
| **Greenspace terms** | Green space OR greenspace* OR greenness OR greenery OR wilderness OR wild land OR natural land OR municipal land OR community land OR public land OR open land OR wild space OR municipal space OR natural space OR open space OR municipal park OR botanic park OR park access OR urban park OR city park OR park availability OR public garden OR natural (within 3 words of) neighbourhood OR natural (within 3 words of) facilities OR vegetation (within 3 words of) natural OR belt (within 3 words of) green OR trial (within 3 words of) recreation OR wild area OR trail (within 3 words of) green OR trail (within 3 words of) cycl* OR trail (within 3 words of) walk OR recreation destination OR recreation opportunities OR physical activity destination OR physical activity resource OR natural area* OR green area* OR walkability* OR built environment OR urban design OR physical activity amenities OR recreation resource OR woodland OR cycle path OR shinrin-yoku OR forest bathing |

**Supplementary figure S1:** Example funnel plot: systolic blood pressure


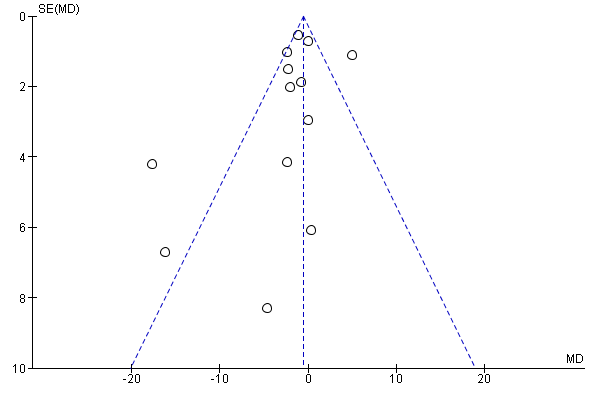


**Supplementary figures S2-S14:** Results from meta-analysis

**Figure S2:** Systolic blood pressure


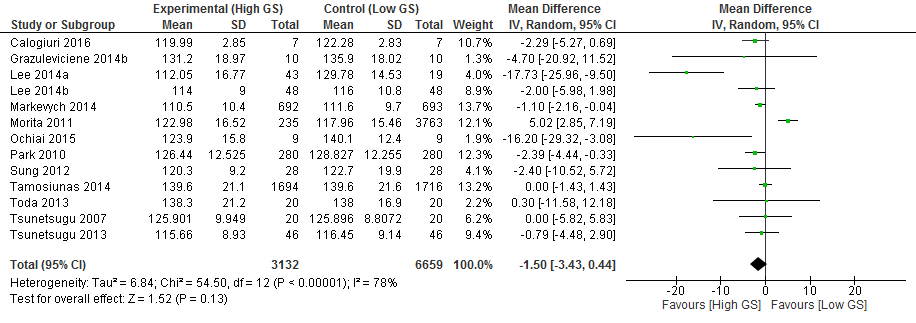


**Figure S3:** Diastolic blood pressure


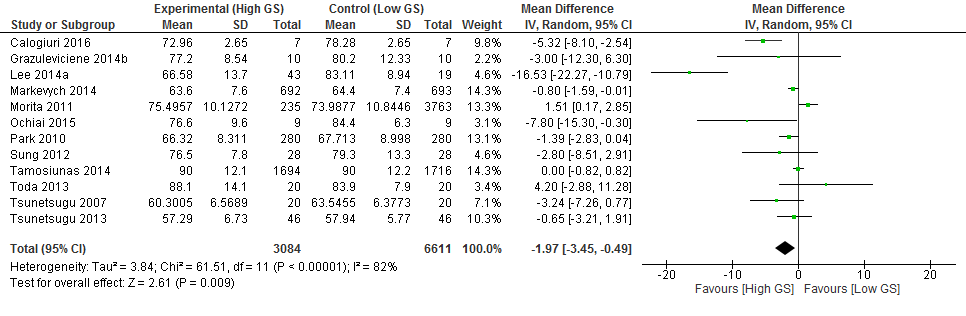


**Figure S4:** Heart rate:


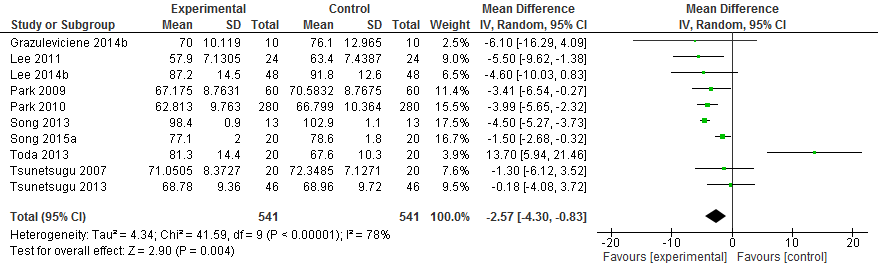


**Figure S5:** Incidence of good self-reported health


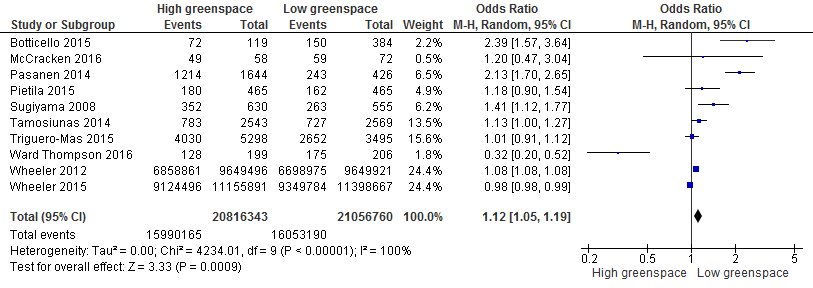


**Figure S6:** Salivary cortisol


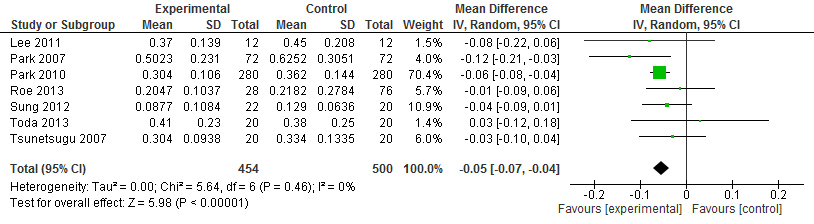


**Figure S7:** Incidence of type II diabetes


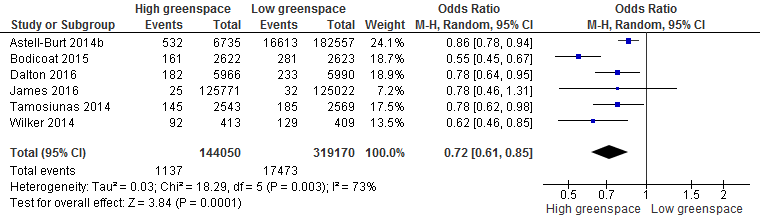


**Figure S8:** Incidence of hypertension


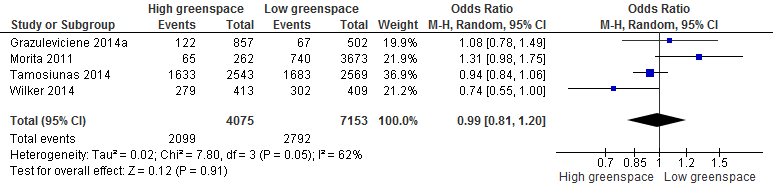


**Figure S9:** Incidence of dyslipidaemia


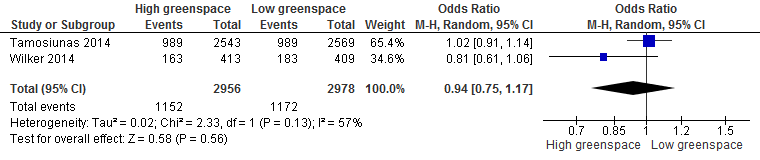


**Figure S10:** Incidence of stroke


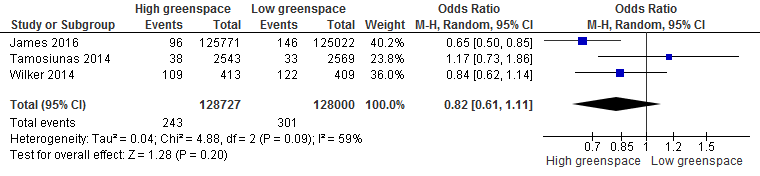


**Figure S11:** Incidence of asthma


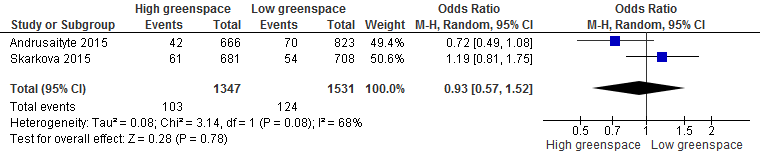


**Figure S12:** All-cause mortality


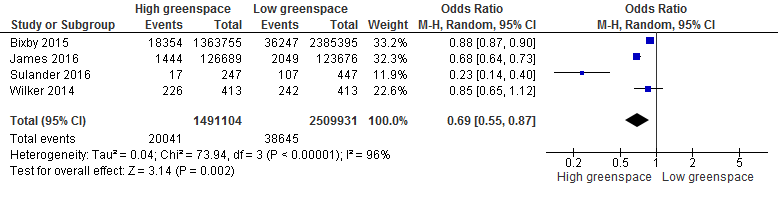


**Figure S13:** Preterm birth


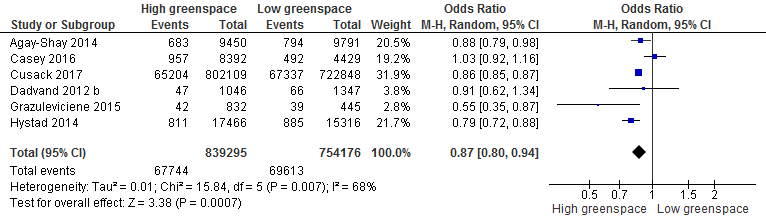


**Figure S14:** Small for gestational age


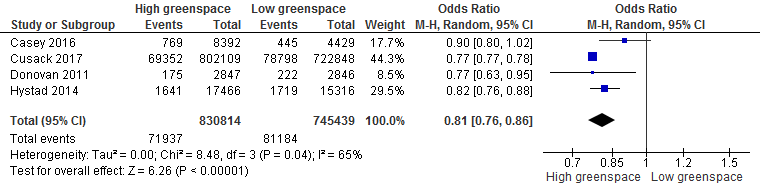


**Figure S15:** Gestational age


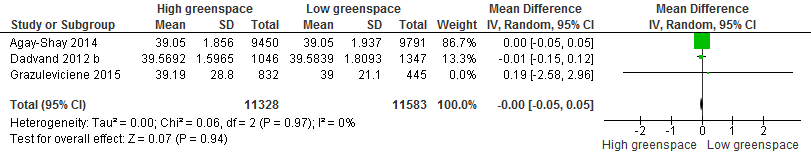


**Figure S16:** Change in HF power of heart rate variability (HRV)


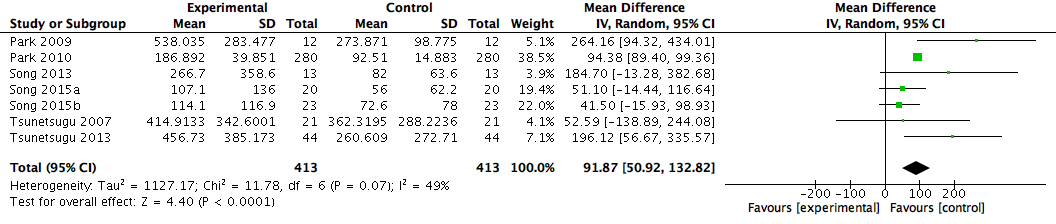


**Figure S17:** LF/(LF+HF) in HRV


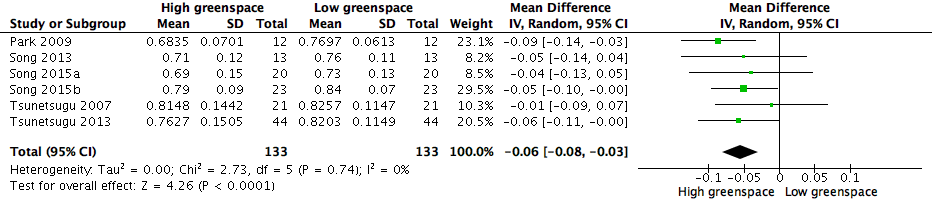


**Figure S18:** Fasting glucose


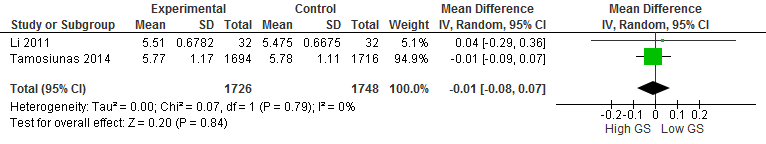


**Figure S19:** Total cholesterol


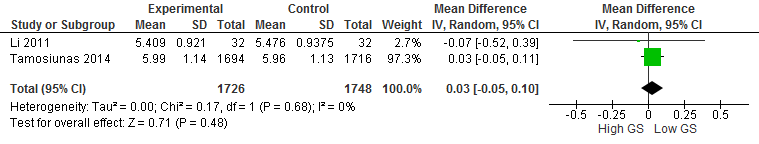


**Figure S20:** HDL cholesterol


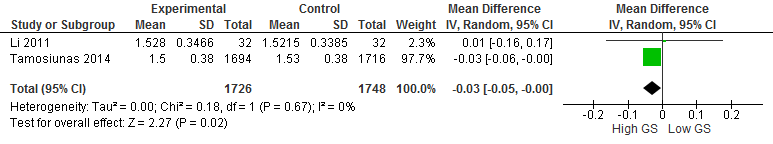


**Figure S21:** LDL cholesterol


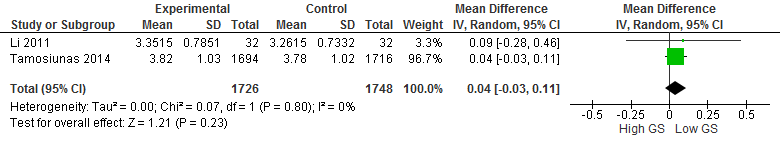


**Figure S22:** Triglycerides


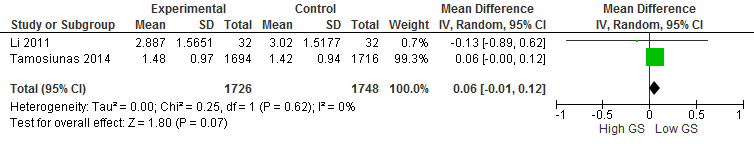


**Figure S23:** HbA1c


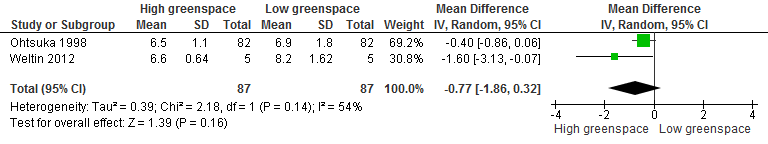


**Figure S24:** Cardiovascular mortality


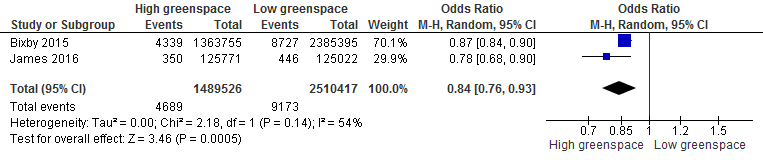


**Figure S25:** Coronary heart disease


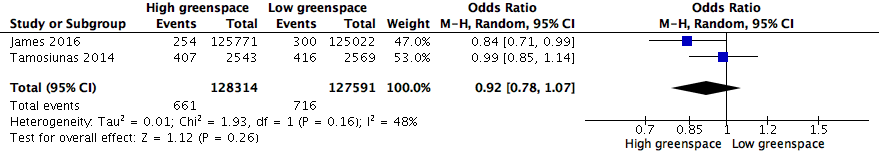


**Supplementary Table S3:** Quality appraisal results for observational studies using an adapted version of the Lachowycz and Jones (2011) quality appraisal checklist

|  | ***Adapted Lachowycz and Jones (2011) quality appraisal checklist*** | | | | | | | | | | |  |
| --- | --- | --- | --- | --- | --- | --- | --- | --- | --- | --- | --- | --- |
| **Lead author, year, *location*** | 1. **Selection bias** | 1. **Inclusion bias** | 1. **Outcome measure** | 1. **Greenspace measure - derivation** | 1. **Greenspace measure - type** | 1. **Use of greenspace** | 1. **Statistical methodology** | 1. **Effect size** | 1. **Multiplicity** | 1. **Level of analysis** | 1. **Greenspace measure** | **Total score** |
| (Agay-Shay et al. 2014) | 1 | 1 | 1 | 1 | 0 | 0 | 1 | 1 | 1 | 1 | 1 | 9 |
| (Agyemang et al. 2007) | 1 | 1 | 1 | 0 | 0 | 0 | 1 | 1 | 0 | 0 | 0 | 5 |
| (Andrusaityte et al. 2016) | 1 | 1 | 1 | 1 | 1 | 0 | 1 | 1 | 1 | 1 | 1 | 10 |
| (Astell-Burt et al. 2014a) | 1 | 1 | 0 | 1 | 1 | 0 | 1 | 1 | 1 | 1 | 1 | 9 |
| (Astell-Burt et al. 2013) | 1 | 1 | 0 | 1 | 1 | 0 | 1 | 1 | 1 | 0 | 1 | 8 |
| (Astell-Burt et al. 2014b) | 1 | 1 | 1 | 1 | 0 | N | 1 | 1 | 1 | 1 | 1 | 9 |
| (Besenyi et al. 2014) | 1 | 1 | 0 | 1 | 1 | 0 | 1 | 1 | 1 | 1 | 1 | 9 |
| (Bijnens et al. 2015) | 1 | 1 | 1 | 1 | 0 | 0 | 1 | 1 | 0 | 1 | 1 | 8 |
| (Bixby et al. 2015) | 1 | 1 | 1 | 1 | 1 | 0 | 1 | 1 | 1 | 0 | 1 | 9 |
| (Bodicoat et al. 2014) | 1 | 1 | 1 | 1 | 1 | 0 | 1 | 1 | 1 | 1 | 1 | 10 |
| (Botticello et al. 2015) | 1 | 1 | 0 | 1 | 1 | 0 | 1 | 1 | 0 | 1 | 1 | 8 |
| (Brown et al. 2016) | 1 | 1 | 1 | 1 | 0 | 0 | 1 | 1 | 1 | 0 | 1 | 8 |
| (Burkart et al. 2016) | 1 | 1 | 1 | 1 | 0 | 0 | 1 | 1 | 0 | 0 | 1 | 7 |
| (Casey et al. 2016) | 1 | 1 | 1 | 1 | 0 | 0 | 1 | 1 | 1 | 1 | 1 | 9 |
| (Chum and O’Campo 2015) | 1 | 1 | 0 | 1 | 0 | 0 | 1 | 1 | 0 | 1 | 1 | 7 |
| (Coutts et al. 2010) | 1 | 1 | 1 | 1 | 1 | 0 | 1 | 1 | 1 | 0 | 1 | 9 |
| (Coutts and Horner 2015) | 1 | 1 | 1 | 1 | 1 | 0 | 1 | 1 | 1 | 1 | 1 | 10 |
| (Cusack et al. 2017) | 1 | 1 | 1 | 1 | 0 | 0 | 1 | 1 | 1 | 1 | 1 | 9 |
| (Dadvand et al. 2012a) | 1 | 1 | 1 | 1 | 0 | 0 | 1 | 1 | 1 | 1 | 1 | 9 |
| (Dadvand et al. 2012b) | 1 | 1 | 1 | 1 | 0 | 0 | 1 | 1 | 1 | 1 | 1 | 9 |
| (Dadvand et al. 2014) | 1 | 1 | 0 | 1 | 0 | 0 | 1 | 1 | 1 | 1 | 1 | 8 |
| (Dadvand et al. 2015) | 1 | 1 | 1 | 1 | 1 | 0 | 1 | 1 | 1 | 1 | 1 | 10 |
| (Dadvand et al. 2016) | 1 | 1 | 0 | 1 | 1 | 0 | 1 | 1 | 1 | 1 | 1+0 | 9 |
| (Dalton et al. 2016) | 1 | 1 | 1 | 1 | 1 | 0 | 1 | 1 | 1 | 1 | 1 | 10 |
| (de Jong et al. 2012) | 1 | 1 | 0 | 1 | 0 | 0 | 1 | 1 | 1 | 1 | 1 | 8 |
| (Demoury et al. 2017) | 1 | 1 | 1 | 1 | 0 | 0 | 1 | 1 | 1 | 1 | 1 | 9 |
| (De Vries et al. 2003) | 1 | 1 | 0 | 1 | 1 | 0 | 1 | 1 | 1 | 1 | 1 | 9 |
| (Donovan et al. 2011) | 1 | 1 | 1 | 1 | 1 | 0 | 1 | 1 | 1 | 1 | 1 | 10 |
| (Droomers et al. 2016) | 1 | 1 | 0 | 1 | 1 | 0 | 1 | 1 | 1 | 1 | 1 | 9 |
| (Dunstan et al. 2013) | 1 | 1 | 0 | 0 | N | 0 | 1 | 1 | 0 | 1 | 1 | 6 |
| (Fuertes et al. 2014) | 1 | 1 | 1 | 1 | 0 | 0 | 1 | N | 1 | 1 | 1 | 8 |
| (Gong et al. 2014) | 1 | 1 | 0 | 1 | N | 0 | 1 | 1 | 1 | 1 | 1 | 8 |
| (Grazuleviciene et al. 2014) | 1 | 1 | 1 | 1 | 0 | 0 | 1 | 1 | 1 | 1 | 1 | 9 |
| (Grazuleviciene et al. 2015a) | 1 | 1 | 1 | 1 | 0 | 0 | 1 | 1 | 1 | 1 | 1 | 9 |
| (Grigsby-Toussaint et al. 2015) | 1 | 1 | 0 | 1 | 0 | 0 | 1 | 1 | 1 | 1 | 1 | 8 |
| (Gutiérrez-Zornoza et al. 2014) | 1 | 1 | 1 | 0 | 0 | 0 | 1 | 1 | 0 | 1 | 1 | 7 |
| (Hoehner et al. 2013) | 1 | 1 | 1 | 1 | N | 0 | 1 | 1 | 0 | 1 | 1 | 8 |
| (Hu et al. 2008) | 1 | 1 | 1 | 1 | 0 | 0 | 1 | 1 | 1 | 0 | 1 | 8 |
| (Hystad et al. 2014) | 1 | 1 | 1 | 1 | 0 | 0 | 1 | 1 | 1 | 1 | 1 | 9 |
| (James et al. 2016) | 1 | 1 | 1 | 1 | 0 | 0 | 1 | 1 | 1 | 1 | 1 | 9 |
| (Jonker et al. 2014) | 1 | 1 | 1 | 1 | 1 | 0 | 1 | 1 | 1 | 1 | 1 | 10 |
| (Kabisch et al. 2016) | 1 | 1 | 1 | 1 | 0 | 0 | 1 | 1 | 1 | 0 | 1 | 8 |
| (Kardan et al. 2015) | 1 | 1 | 0 | 1 | 1 | 0 | 1 | 1 | 1 | 0 | 1 | 8 |
| (Kihal-Talantikite et al. 2013) | 1 | 1 | 1 | 1 | 0 | 0 | 1 | N | 1 | 0 | 1 | 7 |
| (Kim et al. 2016) | 1 | 1 | 1 | 1 | 1 | 0 | 1 | 1 | 1 | 1 | 1 | 10 |
| (Lachowycz and Jones 2014) | 1 | 1 | 1 | 1 | 1 | 0 | 1 | 1 | 1 | 1 | 1 | 10 |
| (Larson et al. 2016) | 1 | 1 | 0 | 1 | 1 | 0 | 1 | 1 | 1 | 0 | 1 | 8 |
| (Laurent et al. 2013) | 1 | 1 | 1 | 1 | 0 | 0 | 1 | 1 | 1 | 1 | 1 | 9 |
| (Lovasi et al. 2008) | N | 1 | 1 | 1 | 1 | 0 | 1 | 1 | 1 | 0 | 1 | 8 |
| (Lovasi et al. 2013) | 1 | 1 | 1 | 1 | 1 | 0 | 1 | 1 | 1 | N | 1 | 9 |
| (Maas et al. 2006) | 1 | 1 | 0 | 1 | 1 | 0 | 1 | 1 | 1 | 1 | 1 | 9 |
| (Maas et al. 2008) | 1 | 1 | 0 | 1 | 1 | 0 | 1 | 1 | 1 | 1 | 1 | 9 |
| (Maas et al. 2009a) | 1 | 1 | 0 | 1 | 0 | 0 | 1 | 1 | 1 | 1 | 1 | 9 |
| (Maas et al. 2009b) | 1 | 1 | 1 | 1 | 0 | 0 | 1 | 1 | 1 | 1 | 1 | 9 |
| (Markevych et al. 2014) | 1 | 1 | 1 | 1 | 0 | 0 | 1 | 1 | 1 | 1 | 1 | 9 |
| (Markevych et al. 2016) | 1 | 1 | 1 | 1 | 1 | 0 | 1 | 1 | 1 | 1 | 1 | 10 |
| (McCracken et al. 2016) | 1 | 1 | 0 | 1 | 1 | 1 | 1 | 1 | 1 | 1 | 1+0 | 10 |
| (Mitchell and Popham 2007) | 1 | 1 | 0 | 1 | n | 0 | 1 | 1 | 1 | 0 | 1 | 7 |
| (Mitchell and Popham 2008) | 1 | 1 | 1 | 1 | 1 | 0 | 1 | 1 | 1 | 0 | 1 | 9 |
| (Mitchell et al. 2011) | 1 | 1 | 1 | 1 | 0 | 0 | 1 | 1 | 1 | 0 | 1 | 8 |
| (Morita et al. 2011) | 1 | 1 | 1 | 1 | 1 | 1 | 1 | 1 | 1 | 1 | 0 | 10 |
| (Ngom et al. 2016) | 1 | 1 | 1 | 1 | 1 | 0 | 1 | 1 | 1 | 1 | 1 | 10 |
| (Padilla et al. 2016) | 1 | 1 | 1 | 1 | 0 | 0 | 1 | 1 | 1 | 0 | 1 | 8 |
| (Paquet et al. 2014) | 1 | 1 | 1 | 1 | 1 | 0 | 1 | 1 | 0 | 1 | 1 | 9 |
| (Pasanen et al. 2014) | 1 | 1 | 0 | 1 | 0 | 0 | 1 | 1 | 1 | 1 | 1 | 8 |
| (Pereira et al. 2012) | 1 | 1 | 1 | 1 | 1 | 0 | 1 | 1 | 1 | 1 | 1 | 10 |
| (Picavet et al. 2016) | 1 | 1 | 1 | 1 | 1 | 0 | 1 | 1 | 1 | 1 | 1 | 10 |
| (Piccolo et al. 2015) | 1 | 1 | 1 | 1 | 1 | 0 | 1 | N | 0 | 1 | 1 | 8 |
| (Pietilä et al. 2015) | 1 | 1 | 0 | 1 | 1 | 0 | 1 | 1 | 1 | 1 | 1 | 9 |
| (Putrik et al. 2015) | 1 | 1 | 0 | 0 | N | N | 1 | 0 | 0 | 1 | 0 | 4 |
| (Reklaitiene et al. 2014) | 1 | 1 | 0 | 1 | 1 | 1 | 1 | 1 | 1 | 1 | 1 | 10 |
| (Requia et al. 2016) | 1 | 1 | 1 | 1 | 1 | 0 | 1 | 1 | 0 | 0 | 1 | 8 |
| (Richardson et al. 2010a) | 1 | 1 | 1 | 1 | 1 | 0 | 1 | 1 | 1 | 0 | 1 | 9 |
| (Richardson and Mitchell 2010b) | 1 | 1 | 1 | 1 | 0 | 0 | 1 | 1 | 1 | 0 | 1 | 8 |
| (Richardson et al. 2012) | 1 | 1 | 1 | 1 | 0 | 0 | 1 | 1 | 1 | 0 | 1 | 8 |
| (Richardson et al. 2013) | 1 | N | 1 | 1 | 0 | 1 | 1 | 1 | 1 | 1 | 1 | 9 |
| (Roe et al. 2013) | N | 1 | 1 | 1 | 1 | 0 | 1 | 1 | 1 | 1 | 1 | 9 |
| (Roe et al. 2016) | 1 | 1 | 0 | 1 | 0 | 1 | 1 | 1 | 0 | 1 | 0 | 7 |
| (Ruokolainen et al. 2015) | 1 | 1 | 1 | 1 | 1 | 0 | 1 | N | 1 | 1 | 1 | 9 |
| (Sbihi et al. 2015) | 1 | 1 | 1 | 1 | 0 | 0 | 1 | 1 | 1 | 1 | 1 | 9 |
| (Skarková et al. 2015) | 1 | 1 | 1 | 1 | 1 | 0 | 1 | 1 | 0 | 0 | 1 | 8 |
| (Stigsdotter et al. 2010) | 1 | 1 | 0 | 1 | 1 | 1 | 1 | 1 | 1 | 1 | 0 | 9 |
| (Sugiyama et al. 2008) | 1 | 1 | 0 | 1 | 1 | 0 | 1 | 1 | 1 | 1 | 0 | 8 |
| (Sugiyama et al. 2009) | 1 | 1 | 0 | 1 | 1 | 0 | 1 | 0 | 0 | 1 | 0 | 6 |
| (Sulander et al. 2016) | 1 | 1 | 1 | 0 | 1 | 1 | 1 | 1 | 1 | 1 | N | 9 |
| (Takano et al. 2002) | 1 | 1 | 1 | 1 | 1 | 0 | 1 | 1 | 0 | 1 | 0 | 8 |
| (Tamosiunas et al. 2014) | 1 | 1 | 1 | 1 | 0 | 1 | 1 | 1 | 1 | 1 | 1 | 10 |
| (Triguero-Mas et al. 2015) | 1 | 1 | 0 | 1 | 0 | 0 | 1 | 1 | 1 | 1 | 1 | 8 |
| (Ulmer et al. 2016) | 1 | 1 | 1 | 1 | 1 | 0 | 1 | 1 | 1 | 1 | 1 | 10 |
| (van Dillen et al. 2012) | 1 | 1 | 0 | 1 | 1 | 0 | 1 | 1 | 1 | 0 | 1 | 8 |
| (Van Herzele and de Vries 2012) | 1 | 1 | 0 | 1 | 1 | 0 | 1 | 1 | 0 | 0 | 0 | 6 |
| (Villeneuve et al. 2012) | 1 | 1 | 1 | 1 | 0 | 0 | 1 | 1 | 1 | 1 | 1 | 9 |
| (Vogt et al. 2015) | 1 | 1 | 0 | 1 | 1 | 0 | 1 | 1 | 1 | 1 | 1 | 9 |
| (Wang et al. 2016) | 1 | 1 | 1 | 1 | 1 | 0 | 1 | 1 | 0 | 1 | 1 | 9 |
| (Ward et al. 2016) | 1 | 1 | 1 | 1 | 1 | 1 | 1 | 1 | 1 | 1 | 1 | 11 |
| (Thompson et al. 2012) | 1 | 1 | 1 | 1 | 1 | 0 | 1 | 1 | 1 | 1 | 1 | 10 |
| (Ward Thompson et al. 2016) | 1 | 1 | 0 | 1 | 0 | 1 | 1 | 1 | 1 | 1 | 1 | 9 |
| (Weimann et al. 2015) | 1 | 1 | 0 | 1 | 1 | 0 | 1 | 1 | 1 | 1 | 1 | 8 |
| (Wheeler et al. 2012) | 1 | 1 | 0 | 0 | N | 0 | 1 | 1 | 0 | 0 | 1 | 5 |
| (Wheeler et al. 2015) | 1 | 1 | 0 | 1 | 1 | 0 | 1 | 1 | 1 | 0 | 1 | 8 |
| (Wilker et al. 2014) | 1 | 1 | 1 | 1 | 0 | 0 | 1 | 1 | 1 | 1 | 1 | 9 |
| (Wolfe et al. 2014) | 1 | 1 | 0 | 0 | 0 | 1 | 1 | 1 | 1 | 1 | 1 | 8 |
| (Wu et al. 2015) | 1 | 1 | 0 | 1 | 0 | 0 | 1 | 1 | 0 | 1 | 1 | 7 |
| (Young et al. 2016) | 1 | 1 | 1 | 1 | 1 | 0 | 1 | 1 | 0 | 1 | 1 | 9 |

**Supplementary Table S4:** Quality appraisal results for intervention studies using an adapted version of the Hanson and Jones and Ogilvie et al. risk of bias tool

|  | ***Adapted Hanson and Jones risk of bias checklist* results** | | | | | | | | | | | |
| --- | --- | --- | --- | --- | --- | --- | --- | --- | --- | --- | --- | --- |
| **Lead author, year, location** | 1. **Reporting: hypothesis** | 1. **Reporting: outcome(s)** | 1. **Reporting: intervention** | 1. **Randomisation** | 1. **Exposure** | 1. **Representativeness** | 1. **Comparability** | 1. **Attrition** | 1. **Outcome assessment tools** | 1. **Follow-up time scale** | 1. **Precision of the results** | **Total score (out of 11)** |
| (Arbillaga-Etxarri et al. 2016) | 1 | 1 | 1 | 1 | N | 1 | N | 1 | 1 | 1 | 1 | 9 |
| (Beil and Hanes 2013) | 1 | 1 | 1 | 1 | 1 | 1 | 1 | 1 | 1 | 1 | 1 | 11 |
| (Calogiuri et al. 2016) | 1 | 1 | 1 | 1 | N | 1 | 1 | 1 | 1 | 1 | 1 | 10 |
| (Fjørtoft 2004) | 1 | 1 | 1 | 0 | N | N | N | 1 | 1 | 1 | 1 | 7 |
| (Grazuleviciene et al. 2015b) | 1 | 1 | 1 | 1 | N | N | 1 | 1 | 1 | 1 | 1 | 9 |
| (Grazuleviciene et al. 2016) | 1 | 1 | 1 | 1 | N | N | 1 | 1 | 1 | 1 | 1 | 9 |
| (Hartig et al. 2003) | 1 | 1 | 1 | 1 | N | N | N | 1 | 1 | 1 | 1 | 8 |
| (Jia et al. 2016) | 1 | 1 | 1 | 1 | N | N | 1 | 1 | 1 | 1 | 1 | 9 |
| (Kim et al. 2015) | 1 | 1 | 1 | 1 | N | 1 | 1 | 1 | 1 | 1 | 1 | 10 |
| (Lee et al. 2011) | 1 | 1 | 1 | 1 | N | N | 1 | 1 | 1 | 1 | 1 | 9 |
| (Lee et al. 2014a) | 1 | 1 | 1 | 0 | N | 1 | 1 | 1 | 0 | 1 | 1 | 8 |
| (Lee and Lee 2014b) | 1 | 1 | 1 | 0 | N | 1 | 1 | 1 | 1 | 1 | 1 | 9 |
| (Li et al. 2008a) | 1 | 1 | 1 | 1 | N | N | 1 | 1 | 1 | 1 | 1 | 9 |
| (Li et al. 2008b) | 1 | 1 | 1 | 1 | N | N | 1 | 1 | 1 | 1 | 0 | 9 |
| (Li et al. 2009) | 1 | 1 | 1 | 1 | N | N | 1 | 1 | 1 | 1 | 0 | 8 |
| (Li et al. 2011) | 1 | 1 | 1 | 0 | N | N | 1 | 1 | 1 | 1 | 1 | 8 |
| (Li et al. 2016) | 1 | 1 | 1 | 1 | N | N | 1 | 1 | 1 | 1 | 1 | 9 |
| (Mao et al. 2012a) | 1 | 1 | 1 | 1 | N | N | 1 | 1 | 1 | 1 | 1 | 9 |
| (Mao et al. 2012b) | 1 | 1 | 1 | 1 | N | N | 1 | 1 | 1 | 1 | 1 | 8 |
| (Matsunaga et al. 2011) | 1 | 1 | 1 | 1 | N | N | 1 | 1 | 1 | 1 | 1 | 9 |
| (Nakau et al. 2013) | 1 | 1 | 1 | 1 | N | 1 | 1 | 0 | 1 | 1 | 1 | 9 |
| (Ochiai et al. 2015) | 1 | 1 | 1 | 1 | N | N | 1 | 1 | 1 | 1 | 1 | 9 |
| (Ohtsuka et al. 1998) | 1 | 1 | 1 | 1 | N | N | 1 | 1 | 1 | 1 | 1 | 9 |
| (Park et al. 2007) | 1 | 1 | 1 | 1 | N | N | 1 | 1 | 1 | 1 | 1 | 9 |
| (Park et al. 2009) | 1 | 1 | 1 | 1 | N | N | 0 | 0 | 1 | 1 | 1 | 7 |
| (Park et al. 2010) | 1 | 1 | 1 | 1 | N | N | 1 | 1 | 1 | 1 | 1 | 9 |
| (Qin et al. 2013) | 1 | 1 | 1 | 1 | N | 1 | 0 | 1 | 1 | 1 | 1 | 9 |
| (Song et al. 2013) | 1 | 1 | 1 | 1 | N | N | 1 | 1 | 1 | 1 | 1 | 9 |
| (Song et al. 2015a) | 1 | 1 | 1 | 1 | N | N | 1 | 1 | 1 | 1 | 1 | 9 |
| (Song et al. 2015b) | 1 | 1 | 1 | 1 | N | N | 1 | 1 | 1 | 1 | 1 | 9 |
| (Sugaya et al. 2011) | 1 | 1 | 1 | 1 | N | N | 1 | 1 | 1 | 1 | 1 | 9 |
| (Sung et al. 2012) | 1 | 1 | 1 | 1 | N | N | 1 | 1 | 1 | 1 | 1 | 9 |
| (Toda et al. 2013) | 1 | 1 | 1 | 1 | N | 1 | 1 | 1 | 1 | 1 | 1 | 10 |
| (Tsunetsugu et al. 2007) | 1 | 1 | 1 | 1 | N | N | 1 | 1 | 1 | 1 | 1 | 9 |
| (Tsunetsugu et al. 2013) | 1 | 1 | 1 | 1 | N | N | 1 | 1 | 1 | 1 | 1 | 9 |
| (Tyrväinen et al. 2014) | 1 | 1 | 1 | 1 | N | N | 1 | 1 | 1 | 1 | 1 | 9 |
| (Ulrich 1984) | 1 | 1 | 1 | 1 | N | 1 | 1 | 1 | 1 | 1 | 1 | 10 |
| (Weltin and Lavin 2012) | 1 | 1 | 1 | 0 | N | N | 1 | 1 | 1 | 1 | 1 | 8 |
| (Yamaguchi et al. 2006) | 1 | 1 | 1 | 1 | N | N | 1 | 0 | 1 | 1 | 1 | 8 |

**References**

Agay-Shay K, Peled A, Crespo AV, Peretz C, Amitai Y, Linn S, et al. 2014. Green spaces and adverse pregnancy outcomes. J Occup Env Med 71:562-569.

Agyemang C, Van Hooijdonk C, Wendel-Vos W, Ujcic-Voortman JK, Lindeman E, Stronks K, et al. 2007. Ethnic differences in the effect of environmental stressors on blood pressure and hypertension in the netherlands. BMC Public Health 7:118.

Andrusaityte S, Grazuleviciene R, Kudzyte J, Bernotiene A, Dedele A, Nieuwenhuijsen MJ. 2016. Associations between neighbourhood greenness and asthma in preschool children in kaunas, lithuania: A case–control study. BMJ Open 6:e010341.

Arbillaga-Etxarri A, Torrent-Pallicer J, Gimeno-Santos E, Barberan-Garcia A, Delgado A, Balcells E, et al. 2016. Validation of walking trails for the urban training tm of chronic obstructive pulmonary disease patients. PloS One 11:e0146705.

Astell-Burt T, Feng X, Kolt GS. 2013. Does access to neighbourhood green space promote a healthy duration of sleep? Novel findings from a cross-sectional study of 259 319 australians. BMJ Open 3:e003094.

Astell-Burt T, Feng X, Kolt GS. 2014a. Neighbourhood green space and the odds of having skin cancer: Multilevel evidence of survey data from 267 072 australians. J Epidemiol Community Health 68:370-374.

Astell-Burt T, Feng X, Kolt GS. 2014b. Is neighborhood green space associated with a lower risk of type 2 diabetes evidence from 267,072 australians. Diabetes Care 37:197-201.

Beil K, Hanes D. 2013. The influence of urban natural and built environments on physiological and psychological measures of stress - a pilot study. Int J Environ Res Public Health 10:1250-1267.

Besenyi GM, Kaczynski AT, Stanis SAW, Bergstrom RD, Lightner JS, Hipp JA. 2014. Planning for health: A community-based spatial analysis of park availability and chronic disease across the lifespan. Health Place 27:102-105.

Bijnens E, Zeegers MP, Gielen M, Kicinski M, Hageman GJ, Pachen D, et al. 2015. Lower placental telomere length may be attributed to maternal residential traffic exposure; a twin study. Environ Int 79:1-7.

Bixby H, Hodgson S, Fortunato L, Hansell A, Fecht D. 2015. Associations between green space and health in english cities: An ecological, cross-sectional study. PloS One 10:e0119495.

Bodicoat DH, O'Donovan G, Dalton AM, Gray LJ, Yates T, Edwardson C, et al. 2014. The association between neighbourhood greenspace and type 2 diabetes in a large cross-sectional study. BMJ Open 4:e006076.

Botticello AL, Rohrbach T, Cobbold N. 2015. Differences in the community built environment influence poor perceived health among persons with spinal cord injury. Arch Phys Med Rehabil 96:1583-1590.

Brown SC, Lombard J, Wang K, Byrne MM, Toro M, Plater-Zyberk E, et al. 2016. Neighborhood greenness and chronic health conditions in medicare beneficiaries. Am J Prev Med 51:78-89.

Burkart K, Meier F, Schneider A, Breitner S, Canario P, Alcoforado MJ, et al. 2016. Modification of heat-related mortality in an elderly urban population by vegetation (urban green) and proximity to water (urban blue): Evidence from lisbon, portugal. Environ Health Perspect 124:927-934.

Calogiuri G, Evensen K, Weydahl A, Andersson K, Patil G, Ihlebæk C, et al. 2016. Green exercise as a workplace intervention to reduce job stress. Results from a pilot study. Work 53:99-111.

Casey JA, James P, Rudolph KE, Wu CD, Schwartz BS. 2016. Greenness and birth outcomes in a range of pennsylvania communities. Int J Environ Res Public Health 13 e311.

Chum A, O’Campo P. 2015. Cross-sectional associations between residential environmental exposures and cardiovascular diseases. BMC Public Health 15:438.

Coutts C, Horner M, Chapin T. 2010. Using geographical information system to model the effects of green space accessibility on mortality in florida. Geocarto Int 25:471-484.

Coutts CJ, Horner MW. 2015. Nature and death: An individual level analysis of the relationship between biophilic environments and premature mortality in florida. Spatial Analysis in Health Geography 295.

Cusack L, Larkin A, Carozza S, Hystad P. 2017. Associations between residential greenness and birth outcomes across texas. Environ Res 152:88-95.

Dadvand P, Sunyer J, BasagaÃ±a X, Ballester F, Lertxundi A, FernÃ¡ndez-Somoano A, et al. 2012a. Surrounding greenness and pregnancy outcomes in four spanish birth cohorts. Environ Health Perspect 120:1481-1487.

Dadvand P, de Nazelle A, Figueras F, Basagana X, Su J, Amoly E, et al. 2012b. Green space, health inequality and pregnancy. Environ Int 40:110-115.

Dadvand P, Villanueva CM, Font-Ribera L, Martinez D, Basagaña X, Belmonte J, et al. 2014. Risks and benefits of green spaces for children: A cross-sectional study of associations with sedentary behavior, obesity, asthma, and allergy. Environ Health Perspect 122:1329-1335.

Dadvand P, Nieuwenhuijsen MJ, Esnaola M, Forns J, Basagaña X, Alvarez-Pedrerol M, et al. 2015. Green spaces and cognitive development in primary schoolchildren. Proc Natl Acad Sci USA 112:7937-7942.

Dadvand P, Bartoll X, Basagaña X, Dalmau-Bueno A, Martinez D, Ambros A, et al. 2016. Green spaces and general health: Roles of mental health status, social support, and physical activity. Environ Int 91:161-167.

Dalton AM, Jones AP, Sharp SJ, Cooper AJ, Griffin S, Wareham NJ. 2016. Residential neighbourhood greenspace is associated with reduced risk of incident diabetes in older people: A prospective cohort study. BMC Public Health 16:1171.

de Jong K, Albin M, Skarback E, Grahn P, Bjork J. 2012. Perceived green qualities were associated with neighborhood satisfaction, physical activity, and general health: Results from a cross-sectional study in suburban and rural scania, southern sweden. Health Place 18:1374-1380.

De Vries S, Verheij RA, Groenewegen PP, Spreeuwenberg P. 2003. Natural environments—healthy environments? An exploratory analysis of the relationship between greenspace and health. Environ Plan A 35:1717-1731.

Demoury C, Thierry B, Richard H, Sigler B, Kestens Y, Parent M-E. 2017. Residential greenness and risk of prostate cancer: A case-control study in montreal, canada. Environ Int 98:129-136.

Donovan GH, Michael YL, Butry DT, Sullivan AD, Chase JM. 2011. Urban trees and the risk of poor birth outcomes. Health Place 17:390-393.

Droomers M, Jongeneel-Grimen B, Kramer D, de Vries S, Kremers S, Bruggink JW, et al. 2016. The impact of intervening in green space in dutch deprived neighbourhoods on physical activity and general health: Results from the quasi-experimental urban40 study. J Epidemiol Community Health 70:147-154.

Dunstan F, Fone DL, Glickman M, Palmer S. 2013. Objectively measured residential environment and self-reported health: A multilevel analysis of uk census data. PloS One 8:e69045.

Fjørtoft I. 2004. Landscape as playscape: The effects of natural environments on children's play and motor development. Child Youth Environ 14:21-44.

Fuertes E, Markevych I, von Berg A, Bauer C-P, Berdel D, Koletzko S, et al. 2014. Greenness and allergies: Evidence of differential associations in two areas in germany. J Epidemiol Community Health 68:787-790.

Gong Y, Gallacher J, Palmer S, Fone D. 2014. Neighbourhood green space, physical function and participation in physical activities among elderly men: The caerphilly prospective study. Int J Behav Nutr Phys Act 11:40.

Grazuleviciene R, Dedele A, Danileviciute A, Vencloviene J, Grazulevicius T, Andrusaityte S, et al. 2014. The influence of proximity to city parks on blood pressure in early pregnancy. Int J Environ Res Public Health 11:2958-2972.

Grazuleviciene R, Danileviciute A, Dedele A, Vencloviene J, Andrusaityte S, Uždanaviciute I, et al. 2015a. Surrounding greenness, proximity to city parks and pregnancy outcomes in kaunas cohort study. Int J Hyg Environ Health 218:358-365.

Grazuleviciene R, Vencloviene J, Kubilius R, Grizas V, Dedele A, Grazulevicius T, et al. 2015b. The effect of park and urban environments on coronary artery disease patients: A randomized trial. Biomed Res Int 2015.

Grazuleviciene R, Vencloviene J, Kubilius R, Grizas V, Danileviciute A, Dedele A, et al. 2016. Tracking restoration of park and urban street settings in coronary artery disease patients. Int J Environ Res Public Health 13:e550.

Grigsby-Toussaint DS, Turi KN, Krupa M, Williams NJ, Pandi-Perumal SR, Jean-Louis G. 2015. Sleep insufficiency and the natural environment: Results from the us behavioral risk factor surveillance system survey. Prev Med 78:78-84.

Gutiérrez-Zornoza M, Sánchez-López M, García-Hermoso A, González-García A, Chillón P, Martínez-Vizcaíno V. 2014. Active commuting to school, weight status, and cardiometabolic risk in children from rural areas: The cuenca study. Health Educ Behav 42:231-239.

Hartig T, Evans GW, Jamner LD, Davis DS, Gärling T. 2003. Tracking restoration in natural and urban field settings. Journal of environmental psychology 23:109-123.

Hoehner CM, Allen P, Barlow CE, Marx CM, Brownson RC, Schootman M. 2013. Understanding the independent and joint associations of the home and workplace built environments on cardiorespiratory fitness and body mass index. Am J Epidemiol 178:1094-1105.

Hu Z, Liebens J, Rao KR. 2008. Linking stroke mortality with air pollution, income, and greenness in northwest florida: An ecological geographical study. Int J Health Geogr 7:20.

Hystad P, Davies HW, Frank L, Loon JV, Gehring U, Tamburic L, et al. 2014. Residential greenness and birth outcomes: Evaluating the influence of spatially correlated built-environment factors. Environ Health Perspect 122:1095-1102.

James P, Hart JE, Banay RF, Laden F. 2016. Exposure to greenness and mortality in a nationwide prospective cohort study of women. Environ Health Perspect 124:1344-1352.

Jia BB, Yang ZX, Mao GX, Lyu YD, Wen XL, Xu WH, et al. 2016. Health effect of forest bathing trip on elderly patients with chronic obstructive pulmonary disease. Biomed Environ Sci 29:212-218.

Jonker MF, van Lenthe FJ, Donkers B, Mackenbach JP, Burdorf A. 2014. The effect of urban green on small-area (healthy) life expectancy. J Epidemiol Community Health 68:999-1002.

Kabisch N, Haase D, Annerstedt van den Bosch M. 2016. Adding natural areas to social indicators of intra-urban health inequalities among children: A case study from berlin, germany. Int J Environ Res Public Health 13:783.

Kardan O, Gozdyra P, Misic B, Moola F, Palmer LJ, Paus T, et al. 2015. Neighborhood greenspace and health in a large urban center. Sci Rep 5:11610.

Kihal-Talantikite W, Padilla CM, Lalloue B, Gelormini M, Zmirou-Navier D, Deguen S. 2013. Green space, social inequalities and neonatal mortality in france. BMC Pregnancy Childbirth 13:191.

Kim BJ, Jeong H, Park S, Lee S. 2015. Forest adjuvant anti-cancer therapy to enhance natural cytotoxicity in urban women with breast cancer: A preliminary prospective interventional study. Eur J Integr Med 7:474-478.

Kim H-J, Min J-Y, Kim H-J, Min K-B. 2016. Parks and green areas are associated with decreased risk for hyperlipidemia. Int J Environ Res Public Health 13:1205.

Lachowycz K, Jones AP. 2014. Does walking explain associations between access to greenspace and lower mortality? Soc Sci Med 107:9-17.

Larson LR, Jennings V, Cloutier SA. 2016. Public parks and wellbeing in urban areas of the united states. PLoS One 11:e0153211.

Laurent O, Wu J, Li L, Milesi C. 2013. Green spaces and pregnancy outcomes in southern california. Health Place 24:190-195.

Lee J, Park BJ, Tsunetsugu Y, Ohira T, Kagawa T, Miyazaki Y. 2011. Effect of forest bathing on physiological and psychological responses in young japanese male subjects. Public Health 125:93-100.

Lee J, Tsunetsugu Y, Takayama N, Park B-J, Li Q, Song C, et al. 2014a. Influence of forest therapy on cardiovascular relaxation in young adults. Evid Based Complement Alternat Med 2014.

Lee J-Y, Lee D-C. 2014b. Cardiac and pulmonary benefits of forest walking versus city walking in elderly women: A randomised, controlled, open-label trial. Eur J Integr Med 6:5-11.

Li Q, Morimoto K, Kobayashi M, Inagaki H, Katsumata M, Hirata Y, et al. 2008a. A forest bathing trip increases human natural killer activity and expression of anti-cancer proteins in female subjects. J Biol Regul Homeost Agents 22:45-55.

Li Q, Morimoto K, Kobayashi M, Inagaki H, Katsumata M, Hirata Y, et al. 2008b. Visiting a forest, but not a city, increases human natural killer activity and expression of anti-cancer proteins. Int J Immunopathol Pharmacol 21:117-127.

Li Q, Kobayashi M, Inagaki H, Hirata Y, Li Y, Hirata K, et al. 2009. A day trip to a forest park increases human natural killer activity and the expression of anti-cancer proteins in male subjects. J Biol Regul Homeost Agents 24:157-165.

Li Q, Otsuka T, Kobayashi M, Wakayama Y, Inagaki H, Katsumata M, et al. 2011. Acute effects of walking in forest environments on cardiovascular and metabolic parameters. Eur J Appl Physiol 111:2845-2853.

Li Q, Kobayashi M, Kumeda S, Ochiai T, Miura T, Kagawa T, et al. 2016. Effects of forest bathing on cardiovascular and metabolic parameters in middle-aged males. Evid Based Complement Alternat Med 2016.

Lovasi GS, Quinn JW, Neckerman KM, Perzanowski MS, Rundle A. 2008. Children living in areas with more street trees have lower prevalence of asthma. J Epidemiol Community Health 62:647-649.

Lovasi GS, O'Neil-Dunne JPM, Lu JWT, Sheehan D, Perzanowski MS, Macfaden SW, et al. 2013. Urban tree canopy and asthma, wheeze, rhinitis, and allergic sensitization to tree pollen in a new york city birth cohort. Environ Health Perspect 121:494-500.

Maas J, Verheij RA, Groenewegen PP, de Vries S, Spreeuwenberg P. 2006. Green space, urbanity, and health: How strong is the relation? J Epidemiol Community Health 60:587-592.

Maas J, Verheij RA, Spreeuwenberg P, Groenewegen PP. 2008. Physical activity as a possible mechanism behind the relationship between green space and health: A multilevel analysis. BMC Public Health 8:206-206.

Maas J, van Dillen SM, Verheij RA, Groenewegen PP. 2009a. Social contacts as a possible mechanism behind the relation between green space and health. Health Place 15:586-595.

Maas J, Verheij RA, de Vries S, Spreeuwenberg P, Schellevis FG, Groenewege PP. 2009b. Morbidity is related to a green living environment. J Epidemiol Community Health 63:967-973.

Mao G, Cao Y, Lan X, He Z, Chen Z, Wang Y, et al. 2012a. Therapeutic effect of forest bathing on human hypertension in the elderly. J Cardiol 60:495-502.

Mao G, Lan X, Cao Y, Chen Z, He Z, Lv Y, et al. 2012b. Effects of short-term forest bathing on human health in a broad-leaved evergreen forest in zhejiang province, china. Biomed Environ Sci 25:317-324.

Markevych I, Thiering E, Fuertes E, Sugiri D, Berdel D, Koletzko S, et al. 2014. A cross-sectional analysis of the effects of residential greenness on blood pressure in 10-year old children: Results from the giniplus and lisaplus studies. BMC Public Health 14:477.

Markevych I, Standl M, Sugiri D, Harris C, Maier W, Berdel D, et al. 2016. Residential greenness and blood lipids in children: A longitudinal analysis in giniplus and lisaplus. Environ Res 151:168-173.

Matsunaga K, Park BJ, Kobayashi H, Miyazaki Y. 2011. Physiologically relaxing effect of a hospital rooftop forest on older women requiring care. J Am Geriatr Soc 59:2162-2163.

McCracken DS, Allen DA, Gow AJ. 2016. Associations between urban greenspace and health-related quality of life in children. Prev Med Rep 3:211-221.

Mitchell R, Popham F. 2007. Greenspace, urbanity and health: Relationships in england. J Epidemiol Community Health 61:681-683.

Mitchell R, Popham F. 2008. Effect of exposure to natural environment on health inequalities: An observational population study. Lancet 372:1655-1660.

Mitchell R, Astell-Burt T, Richardson EA. 2011. A comparison of green space indicators for epidemiological research. J Epidemiol Community Health 65:853-858.

Morita E, Naito M, Hishida A, Wakai K, Mori A, Asai Y, et al. 2011. No association between the frequency of forest walking and blood pressure levels or the prevalence of hypertension in a cross-sectional study of a japanese population. Environ Health Prev Med 16:299-306.

Nakau M, Imanishi J, Imanishi J, Watanabe S, Imanishi A, Baba T, et al. 2013. Spiritual care of cancer patients by integrated medicine in urban green space: A pilot study. Explore (NY) 9:87-90.

Ngom R, Gosselin P, Blais C, Rochette L. 2016. Type and proximity of green spaces are important for preventing cardiovascular morbidity and diabetes-a cross-sectional study for quebec, canada. Int J Environ Res Public Health 13:423.

Ochiai H, Ikei H, Song C, Kobayashi M, Takamatsu A, Miura T, et al. 2015. Physiological and psychological effects of forest therapy on middle-aged males with high-normal blood pressure. Int J Environ Res Public Health 12:2532-2542.

Ohtsuka Y, Yabunaka N, Takayama S. 1998. Shinrin-yoku (forest-air bathing and walking) effectively decreases blood glucose levels in diabetic patients. Int J Biometeorol 41:125-127.

Padilla CM, Kihal-Talantikit W, Perez S, Deguen S. 2016. Use of geographic indicators of healthcare, environment and socioeconomic factors to characterize environmental health disparities. Environ Health 15:79.

Paquet C, Coffee NT, Haren MT, Howard NJ, Adams RJ, Taylor AW, et al. 2014. Food environment, walkability, and public open spaces are associated with incident development of cardio-metabolic risk factors in a biomedical cohort. Health Place 28:173-176.

Park B-J, Tsunetsugu Y, Kasetani T, Hirano H, Kagawa T, Sato M, et al. 2007. Physiological effects of shinrin-yoku (taking in the atmosphere of the forest)-using salivary cortisol and cerebral activity as indicators. J Physiol Anthropol 26:123-128.

Park B-J, Tsunetsugu Y, Kasetani T, Morikawa T, Kagawa T, Miyazaki Y. 2009. Physiological effects of forest recreation in a young conifer forest in hinokage town, japan. Silva Fenn 43:291-301.

Park BJ, Tsunetsugu Y, Kasetani T, Kagawa T, Miyazaki Y. 2010. The physiological effects of shinrin-yoku (taking in the forest atmosphere or forest bathing): Evidence from field experiments in 24 forests across japan. Environ Health Prev Med 15:18-26.

Pasanen TP, Tyrväinen L, Korpela KM. 2014. The relationship between perceived health and physical activity indoors, outdoors in built environments, and outdoors in nature. Appl Psychol Health Well Being 6:324-346.

Pereira G, Foster S, Martin K, Christian H, Boruff BJ, Knuiman M, et al. 2012. The association between neighborhood greenness and cardiovascular disease: An observational study. BMC Public Health 12:466-466.

Picavet HSJ, Milder I, Kruize H, de Vries S, Hermans T, Wendel-Vos W. 2016. Greener living environment healthier people? Exploring green space, physical activity and health in the doetinchem cohort study. Prev Med 89:7-14.

Piccolo RS, Duncan DT, Pearce N, McKinlay JB. 2015. The role of neighborhood characteristics in racial/ethnic disparities in type 2 diabetes: Results from the boston area community health (bach) survey. Soc Sci Med 130:79-90.

Pietilä M, Neuvonen M, Borodulin K, Korpela K, Sievänen T, Tyrväinen L. 2015. Relationships between exposure to urban green spaces, physical activity and self-rated health. JORT 10:44-54.

Putrik P, de Vries N, Mujakovic S, van Amelsvoort L, Kant I, Kunst A, et al. 2015. Living environment matters: Relationships between neighborhood characteristics and health of the residents in a dutch municipality. J Community Health 40:47-56.

Qin J, Zhou X, Sun C, Leng H, Lian Z. 2013. Influence of green spaces on environmental satisfaction and physiological status of urban residents. Urban For Urban Gree 12:490-497.

Reklaitiene R, Grazuleviciene R, Dedele A, Virviciute D, Vensloviene J, Tamosiunas A, et al. 2014. The relationship of green space, depressive symptoms and perceived general health in urban population. Scand J Public Health 42:669-676.

Requia WJ, Roig HL, Adams MD, Zanobetti A, Koutrakis P. 2016. Mapping distance-decay of cardiorespiratory disease risk related to neighborhood environments. Environ Res 151:203-215.

Richardson E, Pearce J, Mitchell R, Day P, Kingham S. 2010a. The association between green space and cause-specific mortality in urban new zealand: An ecological analysis of green space utility. BMC Public Health 10:240.

Richardson EA, Mitchell R. 2010b. Gender differences in relationships between urban green space and health in the united kingdom. Soc Sci Med 71:568-575.

Richardson EA, Mitchell R, Hartig T, de Vries S, Astell-Burt T, Frumkin H. 2012. Green cities and health: A question of scale? J Epidemiol Community Health 66:160-165.

Richardson EA, Pearce J, Mitchell R, Kingham S. 2013. Role of physical activity in the relationship between urban green space and health. 127:318-324.

Roe J, Aspinall PA, Thompson CW. 2016. Understanding relationships between health, ethnicity, place and the role of urban green space in deprived urban communities. Int J Environ Res Public Health 13:e681.

Roe JJ, Ward Thompson C, Aspinall PA, Brewer MJ, Duff EI, Miller D, et al. 2013. Green space and stress: Evidence from cortisol measures in deprived urban communities. Int J Environ Res Public Health 10:4086-4103.

Ruokolainen L, Von Hertzen L, Fyhrquist N, Laatikainen T, Lehtomaki J, Auvinen P, et al. 2015. Green areas around homes reduce atopic sensitization in children. Allergy 70:195-202.

Sbihi H, Tamburic L, Koehoorn M, Brauer M. 2015. Greenness and incident childhood asthma: A 10-year follow-up in a population-based birth cohort. Am J Respir Crit Care Med 192:1131-1133.

Skarková P, Kadlubiec R, Fischer M, Kratenová J, Zapletal M, Vrubel J. 2015. Refining of asthma prevalence spatial distribution and visualization of outdoor environment factors using gis and its application for identification of mutual associations. Cent Eur J Public Health 23:258.

Song C, Joung D, Ikei H, Igarashi M, Aga M, Park BJ, et al. 2013. Physiological and psychological effects of walking on young males in urban parks in winter. J Physiol Anthropol 32:18.

Song C, Ikei H, Kobayashi M, Miura T, Taue M, Kagawa T, et al. 2015a. Effect of forest walking on autonomic nervous system activity in middle-aged hypertensive individuals: A pilot study. Int J Environ Res Public Health 12:2687-2699.

Song C, Ikei H, Igarashi M, Takagaki M, Miyazaki Y. 2015b. Physiological and psychological effects of a walk in urban parks in fall. Int J Environ Res Public Health 12:14216-14228.

Stigsdotter UK, Ekholm O, Schipperijn J, Toftager M, Kamper-Jørgensen F, Randrup TB. 2010. Health promoting outdoor environments -- associations between green space, and health, health-related quality of life and stress based on a danish national representative survey. Scand J Public Health 38:411-417.

Sugaya S, Kasetani T, Zhong Q-J, Wen-Zhi G. 2011. Studies on the amounts of serum hydroperoxide, mmp-3, urinary 8-ohdg, and salivary iga in rheumatoid arthritis patients who experienced shinrin-yoku (forest-air bathing and walking). J Chiba Med Soc 87:181-188.

Sugiyama T, Leslie E, Giles-Corti B, Owen N. 2008. Associations of neighbourhood greenness with physical and mental health: Do walking, social coherence and local social interaction explain the relationships? J Epidemiol Community Health 62:e9-e9.

Sugiyama T, Thompson CW, Alves S. 2009. Associations between neighborhood open space attributes and quality of life for older people in britain. Environ Behav 41:3-21.

Sulander T, Karvinen E, Holopainen M. 2016. Urban green space visits and mortality among older adults. Epidemiology 27:e34-e35.

Sung J, Woo J-M, Kim W, Lim S-K, Chung E-J. 2012. The effect of cognitive behavior therapy-based “forest therapy” program on blood pressure, salivary cortisol level, and quality of life in elderly hypertensive patients. Clin Exp Hypertens 34:1-7.

Takano T, Nakamura K, Watanabe M. 2002. Urban residential environments and senior citizens' longevity in megacity areas: The importance of walkable green spaces. J Epidemiol Community Health 56:913-918.

Tamosiunas A, Grazuleviciene R, Luksiene D, Dedele A, Reklaitiene R, Baceviciene M, et al. 2014. Accessibility and use of urban green spaces, and cardiovascular health: Findings from a kaunas cohort study. Environ Health 13:20.

Thompson CW, Roe J, Aspinall P, Mitchell R, Clow A, Miller D. 2012. More green space is linked to less stress in deprived communities: Evidence from salivary cortisol patterns. Landsc Urban Plan 105:221-229.

Toda M, Den R, Hasegawa-Ohira M, Morimoto K. 2013. Effects of woodland walking on salivary stress markers cortisol and chromogranin a. Complement Ther Med 21:29-34.

Triguero-Mas M, Dadvand P, Cirach M, Martinez D, Medina A, Mompart A, et al. 2015. Natural outdoor environments and mental and physical health: Relationships and mechanisms. Environ Int 77:35-41.

Tsunetsugu Y, Park B-J, Ishii H, Hirano H, Kagawa T, Miyazaki Y. 2007. Physiological effects of shinrin-yoku (taking in the atmosphere of the forest) in an old-growth broadleaf forest in yamagata prefecture, japan. J Physiol Anthropol 26:135-142.

Tsunetsugu Y, Lee J, Park B-J, Tyrväinen L, Kagawa T, Miyazaki Y. 2013. Physiological and psychological effects of viewing urban forest landscapes assessed by multiple measurements. Landscape Urban Plan 113:90-93.

Tyrväinen L, Ojala A, Korpela K, Lanki T, Tsunetsugu Y, Kagawa T. 2014. The influence of urban green environments on stress relief measures: A field experiment. J Environ Psychol 38:1-9.

Ulmer JM, Wolf KL, Backman DR, Tretheway RL, Blain CJ, O’Neil-Dunne JP, et al. 2016. Multiple health benefits of urban tree canopy: The mounting evidence for a green prescription. Health Place 42:54-62.

Ulrich R. 1984. View through a window may influence recovery. Science 224:224-225.

van Dillen S, de Vries S, Groenewegen P, Spreeuwenberg P. 2012. Greenspace in urban neighbourhoods and residents' health: Adding quality to quantity. J Epidemiol Community Health 66:e8.

Van Herzele A, de Vries S. 2012. Linking green space to health: A comparative study of two urban neighbourhoods in ghent, belgium. Popul Environ 34:171-193.

Villeneuve PJ, Jerrett M, J GS, Burnett RT, Chen H, Wheeler AJ, et al. 2012. A cohort study relating urban green space with mortality in ontario, canada. Environ Res 115:51-58.

Vogt S, Mielck A, Berger U, Grill E, Peters A, Döring A, et al. 2015. Neighborhood and healthy aging in a german city: Distances to green space and senior service centers and their associations with physical constitution, disability, and health-related quality of life. Eur J Ageing 12:273-283.

Wang L, Zhao X, Xu W, Tang J, Jiang X. 2016. Correlation analysis of lung cancer and urban spatial factor: Based on survey in shanghai. J Thorac Dis 8:2626-2637.

Ward JS, Duncan JS, Jarden A, Stewart T. 2016. The impact of children's exposure to greenspace on physical activity, cognitive development, emotional wellbeing, and ability to appraise risk. Health Place 40:44-50.

Ward Thompson C, Aspinall P, Roe J, Robertson L, Miller D. 2016. Mitigating stress and supporting health in deprived urban communities: The importance of green space and the social environment. Int J Environ Res Public Health 13:440.

Weimann H, Rylander L, Albin M, Skärbäck E, Grahn P, Östergren P-O, et al. 2015. Effects of changing exposure to neighbourhood greenness on general and mental health: A longitudinal study. Health Place 33:48-56.

Weltin AM, Lavin RP. 2012. The effect of a community garden on hga1c in diabetics of marshallese descent. Journal of Community Health Nursing 29:12-24.

Wheeler BW, White M, Stahl-Timmins W, Depledge MH. 2012. Does living by the coast improve health and wellbeing? Health Place 18:1198-1201.

Wheeler BW, Lovell R, Higgins SL, White MP, Alcock I, Osborne NJ, et al. 2015. Beyond greenspace: An ecological study of population general health and indicators of natural environment type and quality. Int J Health Geogr 14:1.

Wilker E, Wu CD, McNeely E, Mostofsky E, Spengler J, Wellenius G, et al. 2014. Green space and mortality following ischemic stroke. Environ Res 129:42-48.

Wolfe MK, Groenewegen PP, Rijken M, de Vries S. 2014. Green space and changes in self-rated health among people with chronic illness. Eur J Public Health 24:640-642.

Wu Y-T, Prina AM, Jones AP, Barnes LE, Matthews FE, Brayne C. 2015. Community environment, cognitive impairment and dementia in later life: Results from the cognitive function and ageing study. Age Ageing 44:1005-1011.

Yamaguchi M, Deguchi M, Miyazaki Y. 2006. The effects of exercise in forest and urban environments on sympathetic nervous activity of normal young adults. J Int Med Res 34:152-159.

Young C, Laurent O, Chung JH, Wu J. 2016. Geographic distribution of healthy resources and adverse pregnancy outcomes. Matern Child Health J 20:1673-1679.
